# Establishing mouse forebrain organoids as models of intrinsic cortical network assembly

**DOI:** 10.1016/j.stemcr.2026.102832

**Published:** 2026-03-05

**Authors:** Sebastian Hernandez, Hunter E. Schweiger, Isabel Cline, Gregory A. Kaurala, Ash Robbins, Daniel Solis, Samira Vera-Choqqueccota, Jinghui Geng, Tjitse van der Molen, Francisco Reyes, Chinweike Norman Asogwa, Kateryna Voitiuk, Mattia Chini, Marco Rolandi, Sofie R. Salama, Bradley M. Colquitt, Tal Sharf, David Haussler, Mircea Teodorescu, Mohammed A. Mostajo-Radji

**Affiliations:** 1 Genomics Institute, University of California, Santa Cruz, Santa Cruz, CA 95064, USA; 2 Department of Electrical and Computer Engineering, University of California, Santa Cruz, Santa Cruz, CA 95064, USA; 3 Department of Molecular, Cellular and Developmental Biology, University of California, Santa Cruz, Santa Cruz, CA 95064, USA; 4 Department of Chemistry and Biochemistry, University of California, Santa Cruz, Santa Cruz, CA 95064, USA; 5 Department of Biomolecular Engineering, University of California, Santa Cruz, Santa Cruz, CA 95064, USA; 6 Neuroscience Research Institute, University of California, Santa Barbara, Santa Barbara, CA 93106, USA; 7 Department of Molecular, Cellular and Developmental Biology, University of California, Santa Barbara, Santa Barbara, CA 93106, USA; 8 Biotechnology Program, Berkeley City College, Berkeley, CA 94704, USA; 9 Institute of Developmental Neurophysiology, Center for Molecular Neurobiology, University Medical Center Hamburg-Eppendorf, Hamburg, Germany

**Keywords:** brain organoids, cortical development, parvalbumin, mouse organoids, multielectrode arrays

## Abstract

The mouse cortex is a canonical model for studying how functional neural networks emerge, yet it remains unclear which topological features arise from intrinsic cellular organization versus sensory input. Mouse forebrain organoids provide a powerful system to investigate these intrinsic mechanisms. We generated dorsal (DF) and ventral (VF) forebrain organoids from mouse pluripotent stem cells and tracked their development using longitudinal electrophysiology. DF organoids showed progressively stronger network-wide correlations, while VF organoids developed more refined activity patterns with enhanced small-world topology and increased modular organization. Both organoid types form small-world networks, but their topological organization differs. These differences emerge without extrinsic inputs and correlate with Pvalb^+^ interneuron enrichment in VF organoids. Our findings demonstrate how cellular composition influences neural circuit self-organization, establishing mouse forebrain organoids as a tractable platform to study cortical network architecture.

## Highlights

• Mouse forebrain organoids self-organize into small-world neural networks
• Dorsal (DF) and ventral (VF) organoids develop distinct network topologies
• Interneuron enrichment enhances clustering and modularity in VF organoids
• Cellular composition shapes circuit assembly independent of sensory input

## Graphical abstract

**Figure:**
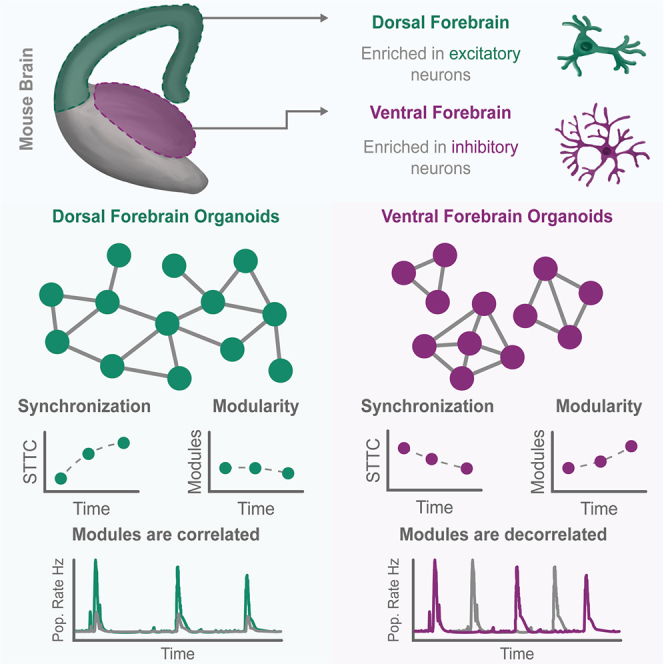

## Teaser

Mostajo-Radji and colleagues generate dorsal and ventral mouse forebrain organoids that self-organize into small-world neural networks. Dorsal organoids, dominated by excitatory neurons, show progressive synchronization, while ventral organoids, enriched in Pvalb^+^ interneurons, develop enhanced clustering and modularity. These findings reveal how cellular composition shapes cortical network topology independent of sensory experience.

## Introduction

The brain is organized as a complex network whose topology supports efficient computation. Across species, a key feature is small-world architecture, defined by high local clustering and short path lengths that enable efficient information segregation and integration with minimal wiring and metabolic costs (Achard et al., 2006; Bassett and Bullmore, 2017; Watts and Strogatz, 1998). This topology is conserved across spatial scales and species, from the *C. elegans* connectome to the brains of mice, cats, macaques, and humans (Bassett and Bullmore, 2017). In humans, the small-world organization of neural circuits changes during development and aging, and its disruption has been linked to neuropsychiatric disorders (Liao et al., 2017). Despite extensive characterization of adult brain networks, the developmental processes that generate this topology remain poorly understood.

Dissociated neuronal cultures have shown that both network topology and cellular composition shape emergent dynamics through self-organization. Effective connectivity networks progressively develop small-world architecture, modular organization, and hub neurons as cultures mature (Antonello et al., 2022; Downes et al., 2012; Schroeter et al., 2015), while excitatory-inhibitory (E-I) balance strongly influences activity patterns (Crocco et al., 2025). Yet, 2D monolayers lack spatial constraints and regional organization of intact tissue, and *in vivo*, it is difficult to separate cellular composition from sensory-driven activity. This makes it challenging to accurately model how 3D cytoarchitecture and cellular diversity alone shape network topology.

Brain organoids derived from pluripotent stem cells (PSCs) provide 3D models that recapitulate key aspects of brain development, including neuronal subtype diversity, layered structure, and functional synaptic networks (Velasco et al., 2019). Most research has focused on human organoids, which generate spontaneous electrical activity that intensifies and becomes more structured with maturation (Alam El Din et al., 2025; Mancinelli et al., 2025; Samarasinghe et al., 2021; Sharf et al., 2022; Trujillo et al., 2019; van der Molen et al., 2026). Longitudinal multielectrode array (MEA) recordings show progressive increases in firing rate, synchrony, and oscillatory complexity across months in culture, paralleling developmental transitions observed in neonatal electroencephalograms (EEGs) (Trujillo et al., 2019; Sharf et al., 2022; Mancinelli et al., 2025; Chini et al., 2022). *In vivo*, developing cortical networks initially exhibit highly synchronized bursts of activity that gradually become more independent as inhibitory circuits mature. This transition, first characterized in mice and later observed in humans, reflects conserved mechanisms through which increasing inhibitory tone refines E-I balance (Wu et al., 2024).

The developing mouse cortex provides well-characterized benchmarks for circuit assembly. Early synchronized activity becomes sparser and less correlated as inhibitory tone increases, while networks acquire hub neurons and small-world topology with dense local clustering and sparse long-range connections (Chini et al., 2022, 2024; Golshani et al., 2009; Hilgetag and Kaiser, 2004; Wu et al., 2024). These features arise before sensory experience, offering valuable reference points for organoid models. Mouse organoids are therefore ideally suited to test how regional cellular composition shapes emergent network topology.

Efforts to model mouse forebrain development with organoids date back over two decades but remain technically limited. The Sasai group first described PSC-derived mouse forebrain organoids in 2005, later refining the protocol using Glasgow Minimum Essential Medium (GMEM)-based conditions (Watanabe et al., 2005; Eiraku et al., 2008). Subsequent studies followed this approach (Elliott et al., 2023; Ferdaos et al., 2022; Mall et al., 2017; Park et al., 2024) or used reaggregated primary progenitors (Mostajo-Radji et al., 2025; Tanveer et al., 2025). Other efforts produced unguided organoids with partial forebrain identity (Lindhout et al., 2025; Sánchez et al., 2024) or achieved limited cortical induction (Medina-Cano et al., 2022). More recently, N2B27-based protocols have generated organoids with cortical projection neurons (PNs) that persist for up to 40 days (Li et al., 2020; Medina-Cano et al., 2025). Nonetheless, protocols that yield mouse forebrain organoids with sustained network activity and functional synaptic connectivity are still lacking.

Here, we address a key gap in the field by systematically investigating how regional cellular composition shapes the formation of neural networks independent of sensory input. We established a protocol that generates dorsal (DF) and ventral (VF) forebrain organoids with robust, long-lasting neural activity from mouse PSCs and characterized their network development. Despite differences in initial patterning, both organoids generate neocortical cell types but with markedly different inhibitory neuron content, providing complementary systems to investigate how E-I balance influences cortical circuit assembly. DF organoids, composed primarily of excitatory PNs, exhibit progressive synchronization, whereas VF organoids, enriched in GABAergic interneurons (INs; including Pvalb^+^ INs), show refined temporal coordination, enhanced small-world topology, and increased modular organization with stable connectivity patterns. Although both types form small-world networks, they differ in topological organization, demonstrating how cellular composition influences circuit formation. These findings establish mouse forebrain organoids as a tractable platform to study how neuronal populations shape cortical network architecture and to probe the developmental logic underlying circuit assembly and its potential disruption in disease. Importantly, this platform allows mechanistic testing not feasible in human organoids, including causal perturbations and *in vivo* validation using genetically identical controls.

## Results

### A standardized protocol for DF organoid generation

**Figure 1:**
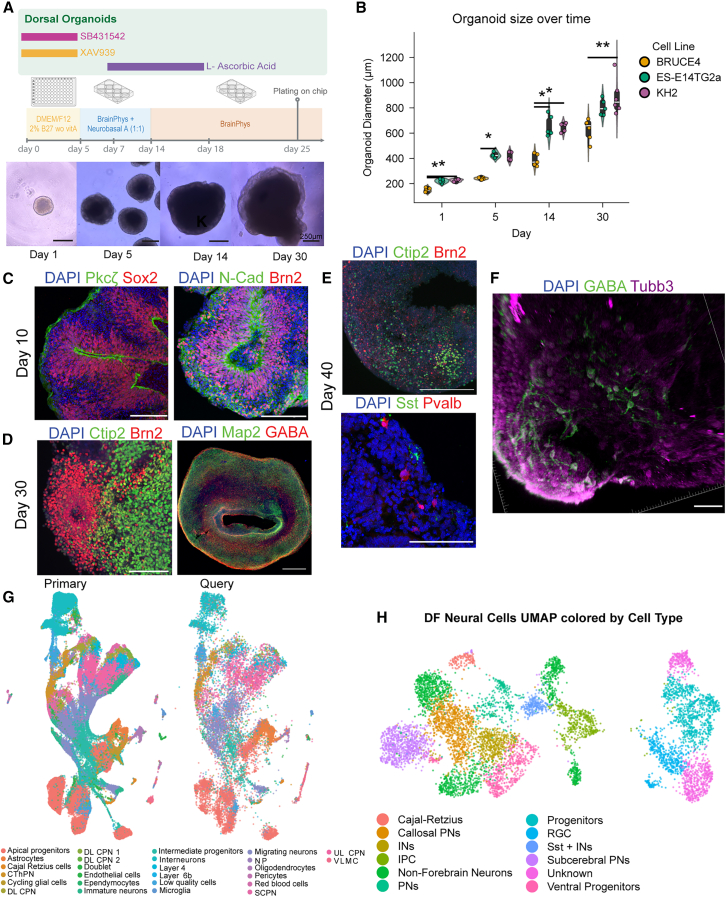
An optimized protocol for DF organoid development (A) Schematic of the protocol for DF organoid development. (B) Organoid diameter across 3 cell lines at days 1, 5, 14, and 30. ^∗^*p* < 0.05, ^∗∗^*p* < 0.003, Tukey’s HSD. *n* = 5 organoids for each cell line. (C) IHC of DF organoids at day 10 stained for: (left) Pkcζ (green) and Sox2 (red); (right) N-cadherin (green) and Brn2 (red). (D) Day 30 DF organoids stained for: (left) Ctip2 (green) and Brn2 (red); (right) Map2 (green) and GABA (red). (E) Day 40 DF organoids stained for (top) Ctip2 (green) and Brn2 (red); (bottom) Sst (green) and Pvalb (red). (F) 3D reconstruction of day 40 DF organoid stained for GABA (green) and Tubb3 (magenta). DAPI nuclear counterstain shown in blue. Scale bars, 100 μm. (G) Anchor-based label transfer mapping between primary tissue (developing mouse cerebral cortex) and organoid (DF organoids) datasets. DL CPN, deep layer callosal PN; UL CPN, upper layer callosal PN; SCPN, subcerebral PN; CThPN, corticothalamic PN; VLMC, vascular and leptomeningeal cells. *n* = 17,970 cells. (H) UMAP visualization of neural lineage cell types in DF organoids. IPC, intermediate progenitor; RGC, radial glial cell. *n* = 5,349 cells. See also Figures S1–S3; Video S1.

GMEM-based DF organoids generate active neurons, but these are sparse and unsuitable for modeling circuit-level dynamics (Eiraku et al., 2008; Park et al., 2024). To address this, we established a robust protocol for generating DF organoids by aggregating 3,000 mouse embryonic stem cells (mESCs) per well, inducing DF identity via WNT and TGFβ inhibition, and promoting neuronal differentiation through a stepwise Neurobasal-A/BrainPhys to BrainPhys media transition (Figure 1A; see “methods”). This protocol increased radial glia abundance relative to GMEM-based methods, indicating improved DF specification, while reducing off-target lineages and enhancing neuronal yield (Figure S1).

We tested the protocol in 3 genetically distinct mESC lines: BRUCE4 (C57BL/6 background), ES-E14TG2a (129/Ola background), and KH2 (C57BL/6 × 129/Sv hybrid background). DF organoids yielded consistent sizes over time, with the BRUCE4 line being slightly smaller than the other 2 lines (Figure 1B). Immunohistochemistry (IHC) revealed stage-specific marker expression in DF organoids. By day 10, Sox2^+^ progenitors, Pkcζ^+^ polarity, and N-cadherin^+^ neuroepithelium were evident (Figures 1C, S2A, and S2B). Organoids expressed the intermediate progenitor marker Tbr2, the neuronal marker Tubb3, and DF markers Tbr1 and Brn2 (Figures 1C and S2B–S2D), reflecting mid corticogenesis with deep-layer Tbr1^+^ PNs and upper-layer Brn2^+^ progenitors and PNs (Di Bella et al., 2021). Small GABA^+^ IN populations were also detected (Figure S2E). By days 30–40, organoids expressed Ctip2 and Brn2 in corticofugal and callosal PNs, respectively, and contained Gfap^+^ astrocytes and GABA^+^ INs (Figures 1D, 1E, and S2F–S2H). Consistent with prior reports, small populations of Pvalb^+^ and Sst^+^ INs appeared, suggesting that 3D environments promote their maturation (Figure 1E) (Mostajo-Radji et al., 2025; Walsh et al., 2025). 3D image reconstruction showed Tubb3^+^ neuronal projections including GABA^+^ and GABA^–^ neurons (Figure 1F; Video S1).

Video S1. 3D image reconstruction of a day 40 DF organoid, related to Figure 1 Immunostaining markers shown are GABA (green), Tubb3 (magenta), and DAPI (blue). Scale bars, 50 μm.

Single-cell RNA sequencing (scRNAseq) of DF organoids at days 16, 30, and 60 yielded 17,970 transcriptomes across the 3 cell lines (Figure S3A). UMAP visualization identified neurons, progenitors, glia, and off-target non-neural cells (Figures S3B and S3C). Cellular diversity increased over time and remained consistent across lines (Figures S3D–S3F). Anchor-based label transfer to a developing mouse cortex reference (E10.5 to P4; Di Bella et al., 2021) confirmed alignment with *in vivo* cell types (Figures 1G, S3G, and S3H). Neural subclustering revealed diverse subtypes, including *Reelin*^*+*^ Cajal-Retzius cells, *Slc17a6*^*+*^ maturing PNs, *Crim1*^*+*^ and *Lrrtm4*^*+*^ corticofugal PNs, *Gad1*^*+*^*/Gad2*^*+*^/Sst^+^ INs, *Stmn2*^*+*^/*Map2*^*+*^ non-forebrain neurons (∼25%–30% of neurons), *Mfng*^*+*^ intermediate progenitors, *Vim*^*+*^/*Hes6*^*+*^ radial glia, *Sox2*^*+*^ progenitors (Figures 1H and S3I), suggesting robust forebrain specification across diverse genetic backgrounds.

### Progressive network maturation in DF organoids

**Figure 2:**
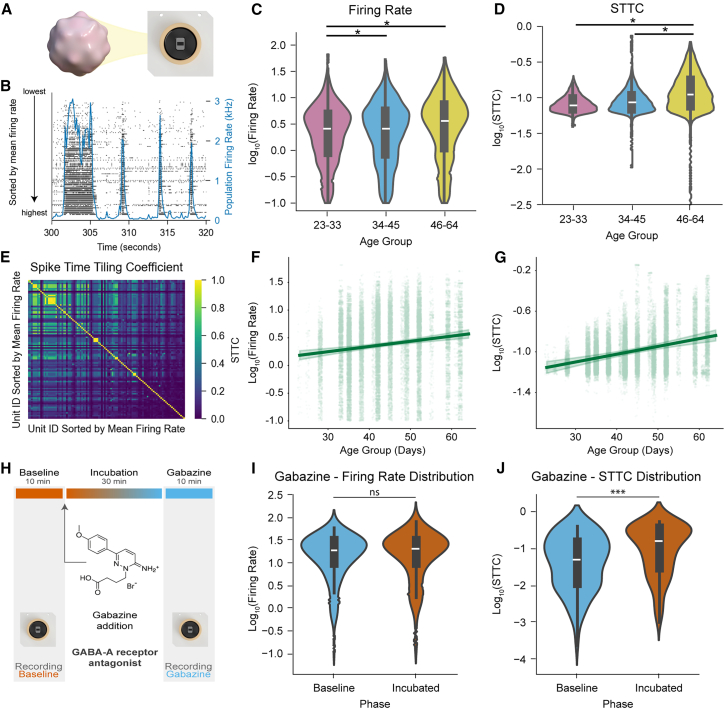
Neuronal activity and E-I balance in DF organoid networks (A) Schematic of the MEA recording setup. (B) Representative raster plot showing neuronal activity, with the population firing rate over time (blue). Units sorted by mean firing rate. (C and D) Violin plots showing log transformed mean firing rates (Hz) (C) and log-transformed mean STTC (D) over early (23–33 days), mid (34–45 days), and late (46–64 days). *n* = 16 organoids, 28,809 units. (E) STTC matrix showing pairwise spike train correlations, sorted by mean firing rate. (F and G) Linear mixed-effects model predicted line plot of the log-transformed mean firing rate distribution (F) and log-transformed STTC (G). ns, not significant, ^∗^ significant after Bonferroni correction, *p* < 0.017, mixed-effects model (D–G). Data shown as mean ± CI. (H) Schematic of the recording protocol: 10-min baseline recording, 30-min drug incubation, and 10-min post-incubation recording. (I and J) Violin plots showing (I) firing rates and (J) STTC distributions during baseline (blue) and post-Gabazine (orange). (*n* = 3 organoids, 133 units). ns, not significant, ^∗∗∗^*p* < 0.0001, mixed-effects models. See also Figures S4–S7; Tables S1–S4.

To characterize the progressive maturation of network activity patterns in DF organoids, we performed longitudinal extracellular recordings using high-density MEAs (Figure 2A). These arrays contain 26,400 recording sites and provide simultaneous readout from 1,024 channels, enabling network-level analysis with single-cell resolution.

Neural activity was analyzed across three developmental stages: early (days 23–33; 15 recordings; 3,678 putative neurons), intermediate (days 34–45; 55 recordings; 16,281 putative neurons), and late (days 46–64; 49 recordings; 10,037 putative neurons). Network function was quantified using two metrics: firing rate, reflecting single-neuron activity, and the spike-time tiling coefficient (STTC) with a 10 ms window, capturing pairwise temporal correlations independent of firing rate (Figures 2B–2D). Mean firing rates rose progressively, consistent with *in vivo* trends (Figure 2C) (Chini et al., 2022, 2024). STTC values increased in mean and variability over development, consistent with the emergence of structured, heterogeneous connectivity patterns (Figures 2D and 2E; Table S1). Both measures followed log-normal distributions, consistent with fundamental electrophysiological principles (Buzsáki and Mizuseki, 2014) (Figure S4). Maturation patterns were conserved across organoids from the 3 cell lines (Table S2). Some line-specific differences appeared at individual stages, but linear mixed-effects modeling revealed no significant divergence in developmental slopes for either firing rate or STTC (Figures 2F, 2G, and S5).

The progressive increase in synchronization contrasts with *in vivo* cortical development, where early network-wide synchrony transitions to sparse, decorrelated subnetwork activity (Chini et al., 2022, 2024; Golshani et al., 2009). This developmental decorrelation is thought to arise from the maturation and integration of inhibitory INs, which shift the excitatory-inhibitory balance toward inhibition (Chini et al., 2022; Wu et al., 2024). DF organoids contain relatively sparse IN populations, suggesting that insufficient inhibitory tone may prevent decorrelation. Similar sustained synchronization has been observed in human cortical organoids (Osaki et al., 2024; Trujillo et al., 2019; van der Molen et al., 2026), suggesting this may reflect a general constraint of organoid systems with limited IN content.

To investigate whether E-I balance influences network dynamics, we pharmacologically manipulated their synaptic transmission. Dimethyl sulfoxide (DMSO) vehicle control and 2-amino-5-phosphonovaleric acid (APV; NMDA receptor antagonist) had no significant effects on STTC (Tables S3 and S4; Figures S6A and S6B). In contrast, 2,3-dihydroxy-6-nitro-7-sulfamoyl-benzo[f]quinoxaline (NBQX; AMPA/Kainate antagonist) significantly reduced network connectivity (*p* < 0.003; Figure S6C; Table S4), consistent with the dominant role of AMPA receptors in cortical excitatory transmission. To examine inhibition, we blocked GABA_A_ receptors with Gabazine, artificially elevating the E-I ratio (Figure 2H). Gabazine increased STTC significantly (*p* < 0.001) while leaving firing rates unchanged (*p* = 0.79; Figures 2I, 2J, and S6D; Tables S3 and S4), and prolonged burst durations (Figure S7). These results indicate that inhibitory tone modulates network synchronization in DF organoids.

### Generation and characterization of VF organoids

**Figure 3:**
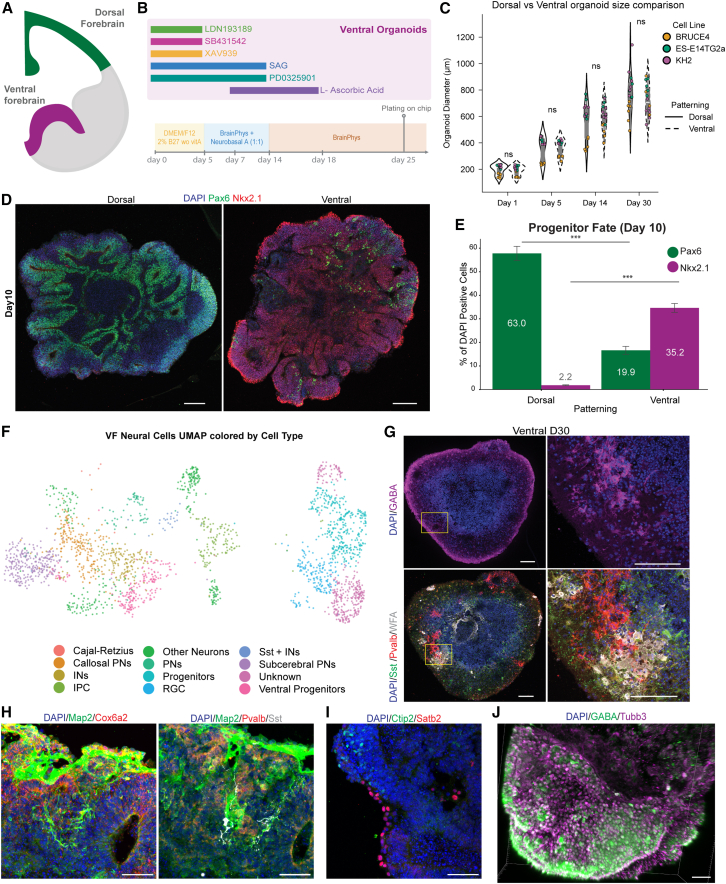
Characterization of the VF organoid development protocol (A) Schematic representation of DF (green) and VF (purple) regions. (B) Schematic of the protocol for VF organoid development. (C) Organoid diameter over time for DF and VF organoids for all 3 cell lines. Student’s *t* test, ns, not significant. (D) IHC of day 10 organoids showing DF marker Pax6 (green) and VF marker Nkx2.1 (red). (*n* = 20 organoids from 4 different batches for both DF and VF). (E) Quantification of Pax6^+^ and Nkx2.1^+^ cells across DF and VF patterned organoids. *n* = 20 organoids from 4 different batches for both DF and VF, ^∗∗∗^*p* < 0.0001; Mann-Whitney *U* test. Data are shown as mean ± SEM. (F) UMAP visualization of neural populations from scRNA-seq in VF organoids. IPC, intermediate progenitor; RGC, radial glial cell. *n* = 2,211 cells. (G) IHC of day 30 VF organoids showing: (top) GABA (magenta); (bottom) Sst (green), Pvalb (red), and WFA (gray) in the following section. (H) IHC of day 30 VF organoids showing: (left) Map2 (green), Cox6a2 (red); (right) Map2 (green), Pvalb (red), and Sst (gray) in the following section. (I) IHC of day 30 VF organoids showing Ctip2 (green) and Satb2 (red). (J) 3D reconstruction of day 40 VF organoid stained for GABA (green) and Tubb3 (magenta). DAPI nuclear counterstain is shown in blue. Scale bars, 50 μm. See also Figures S8–S10; Video S2; Table S5.

To investigate the role of INs in network formation, we generated VF organoids by treating forebrain progenitors with Smoothened Agonist (SAG), a potent Sonic hedgehog (SHH) activator, during the first 14 days of differentiation (Figures 3A and 3B). To further bias toward Pvalb^+^ identity, we co-treated with MEK/ERK pathway inhibitor PD0325901 (Holter et al., 2021).

SAG treatment induced a significant shift in regional identity by day 10 while maintaining similar architecture and viability (Figures 1C, S8, and S9). VF organoid diameters showed some cell line variation early in development, with BRUCE4 being smaller than ES-E14TG2a and KH2 at days 1, 5, and 14 (*p* < 0.05; Figure S9A). However, overall organoid sizes were comparable between DF and VF types across development (*p* > 0.5; Figure 3C; Table S5). In addition, viability testing via acridine orange (live) and propidium iodide (dead) was qualitatively similar across maturation stages (Figures S9B–S9E).

IHC quantification at day 10 showed Pax6 expression was higher in DF than VF organoids (DF = 57.78 ± 38.20%; VF = 16.64 ± 17.81%; *p* < 0.001), while Nkx2.1 was enriched in VF organoids (DF = 1.82 ± 2.12%; VF = 34.64 ± 20.06%; *p* < 0.001; Figures 3D, 3E, S10A, and S10B).

scRNAseq at days 16, 30, and 60 integrated 17,148 DF and 10,945 VF cells (Figures S10C–S10F), including neural populations and non-neural “other” cells from off-target lineages. When the neural population from VF organoids was reclustered and projected onto the same UMAP coordinates as DF organoids (Figure 1H), VF cells occupied the same major neural clusters, confirming that both organoid types generate comparable forebrain cell types (Figure 3F). To validate IN identity, we performed IHC on serial cryosections and confirmed strong GABA expression in the same regions as Pvalb^+^ and Sst^+^ cells (Figure 3G). Wisteria floribunda agglutinin (WFA) labeling revealed extensive perineuronal net formation surrounding Pvalb^+^ INs (Figure 3G), indicating functional maturation. Additionally, we validated the presence of the recently described Pvalb^+^ IN marker Cox6a2 (Mostajo-Radji et al., 2025) by IHC staining in different serial sections, showing extensive colocalization in regions of Pvalb expression (Figure 3H). We also confirmed that VF organoids generated the same PN subtypes as DF organoids, with Ctip2^+^ and Satb2^+^ cells present in qualitatively lower numbers (Figure 3I). Finally, 3D image reconstruction showed robust colocalization of GABA^+^/Tubb3^+^ projections (Figure 3J; Video S2). Altogether, we conclude that VF organoids showed strong IN specification.

Video S2. 3D image reconstruction of a day 40 VF organoid, related to Figure 3 Immunostaining markers shown are GABA (green), Tubb3 (magenta), and DAPI (blue). Scale bars, 50 μm.

### DF and VF organoids exhibit distinct network dynamics

**Figure 4:**
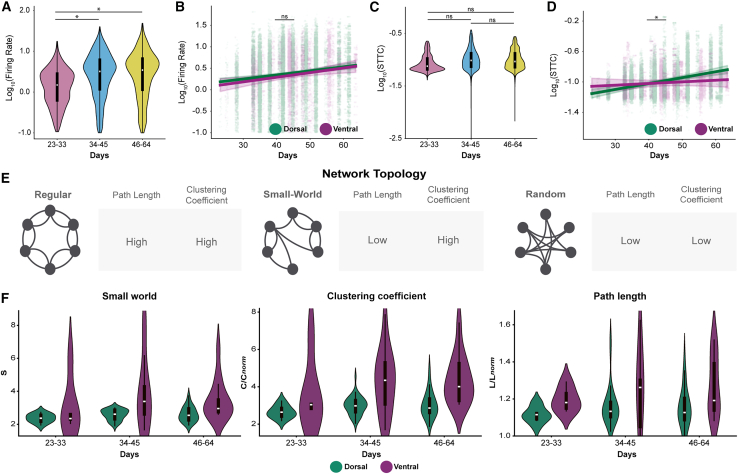
DF and VF organoids follow distinct developmental trajectories in neural dynamics (A) Violin plots showing the distribution of log-transformed firing rates across three developmental stages in organoids: 23–33 days (pink), 34–45 days (blue), and 46–64 days (yellow). *n* = 18 organoids, 7,489 units. (B) Scatterplot with regression lines (LME) showing the relationship between log-transformed firing rate (*y* axis) and age in days (*x* axis) for DF (green; *n* = 16 organoids, 28,809 units) and VF (purple; *n* = 18 organoids, 7,489 units) organoids. Individual data points represent recorded units. “ns” indicates non-significant difference between the slopes of DF and VF. (C) Same as (A) but for difference between log-transformed STTC values. (D) Same as (B) but for difference between the log-transformed STTC slopes. ns, not significant, ^∗^*p* < 0.017, ^∗∗^*p* < 0.003, ^∗∗∗^*p* < 0.0003 (Bonferroni corrected in A and C), mixed-effects model. (E) Schematic representations of different network topologies: regular (top), small-world (middle), and random (bottom). (F) Violin plots showing the distribution of (left) small-world index (S) for DF (green; *n* = 16 organoids) and VF (purple; *n* = 18 organoids), (middle) clustering coefficient (C), and (right) path length (L). ns, not significant, ^∗^*p* < 0.05, ^∗∗^*p* < 0.01, ^∗∗∗^*p* < 0.001, mixed-effects model. See also Figure S11; Tables S6–S10.

To understand the contribution of INs to circuit formation in organoids, we compared the electrophysiological development of VF organoids and DF organoids at the same time points used for DF recordings (Figure 2). In VF organoids, log-transformed mean firing rates significantly increased from early to mid (*p* < 0.001) and early to late stages (*p* < 0.002), but not between mid and late development (*p* = 0.76; Figure 4A; Table S6).

When comparing firing rates between VF and DF organoids at matched time points (using data from Figure 2C), we found no significant differences at early or mid-stages (*p* > 0.5; Table S6). However, DF organoids displayed modest but statistically significant higher firing rates at late stages (*p* = 0.02; Table S6). Mixed-effects modeling of age-related changes in firing rates showed no significant difference in developmental slopes (*p* = 0.74) or intercepts (*p* = 0.54), indicating overall similar developmental dynamics between the two types of organoids (Figure 4B; Table S7).

In contrast, network synchrony as measured by STTC displayed a fundamentally different developmental trend. VF organoids remained relatively stable across development (Figure 4C), whereas DF organoids exhibited a steady increase (Figure 2D; Tables S6 and S7). Mixed-effects analysis confirmed a significant difference in the rate of change (slope) between DF and VF STTC values (*p* = 0.04), while intercepts were not significantly different (*p* = 0.06; Figure 4D; Tables S6 and S7). These results suggest that elevated inhibitory tone in VF organoids alters circuit refinement compared to DF organoids. However, unlike progressive decorrelation observed *in vivo* (Chini et al., 2024; Golshani et al., 2009; Wu et al., 2024), neither organoid model displayed continual synchrony reduction, pointing to the importance of sensory input or external factors to fully recapitulate developmental dynamics.

To validate that differences in IN composition underlie these divergent trajectories, we performed qPCR measuring the expression of *Gad2* (pan-GABAergic) and *Pvalb* relative to *Camk2a* (pan-excitatory) across the same three developmental stages. VF organoids consistently exhibited higher relative expression ratios for both *Gad2/Camk2a* and *Pvalb/Camk2a* compared to DF organoids at all time points (Figure S11). This molecular signature of elevated inhibitory content parallels the electrophysiological divergence in STTC trajectories, with VF organoids maintaining stable synchrony while DF organoids show progressive increases, consistent with the constraining effect of inhibitory tone on network correlation.

### VF organoids develop stronger small-world topology through enhanced local clustering

*In vivo*, mouse cortical networks acquire small-world topology during early postnatal development before sensory experience (Chini et al., 2024; Hilgetag and Kaiser, 2004). To determine whether organoids recapitulate this trajectory and whether cellular composition influences topology, we analyzed small-world organization across DF and VF development.

The small-world index (*S* = *C*_*norm*_/*L*_*norm*_) quantifies the balance between local clustering (*C*) and path length (*L*) (Bassett and Bullmore, 2017) (Figure 4E). Values significantly greater than 1 indicate small-world organization. For each recording, we generated 1,000 surrogate datasets by shuffling neuron IDs while preserving firing rates (Okun et al., 2012; 2015). STTC values exceeding the 90th percentile were included in binary adjacency matrices. *S* values increased progressively in both organoid types (Tables S8 and S9). VF organoids showed significantly higher *S* than DF at all stages (*p* < 0.003 in all cases; Figure 4F). Analysis of *L*_*norm*_ and *C*_*norm*_ revealed that *C*_*norm*_ is the primary determinant of this difference (*p* < 0.001). VF organoids exhibited significantly higher *C*_*norm*_, particularly at late stages (*p* < 0.003; Table S10). This enhanced clustering is consistent with *in vivo* observations where inhibitory integration increases clustering coefficients, suggesting the elevated inhibitory content in VF organoids is associated with enhanced topological organization.

### Divergent network specialization in DF and VF organoids

**Figure 5:**
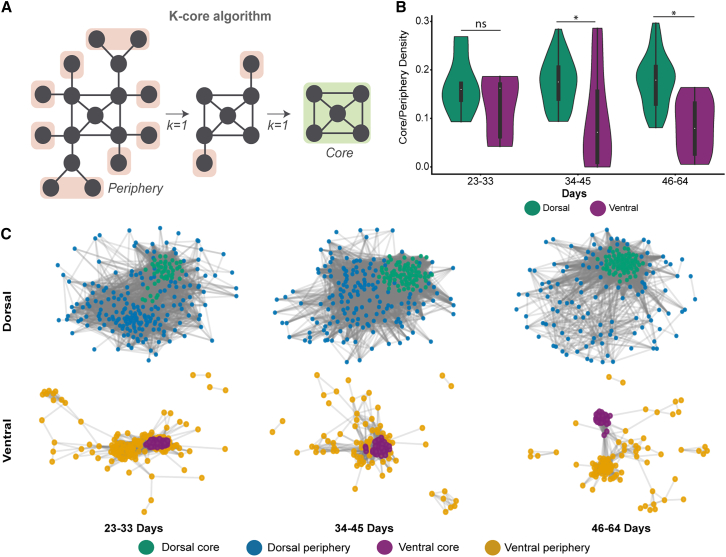
Divergent core-periphery organization reveals distinct network specialization in DF and VF organoids (A) Schematic of the *k*-core algorithm used to identify core and peripheral regions within neural networks. (B) Violin plots showing core and periphery density across developmental stages (23–33; 34–45; and 46–64 days) for DF (green; *n* = 16 organoids) and VF (purple; *n* = 18 organoids) organoids. ns, not significant; ^∗^*p* < 0.05, mixed-effects model. (C) Representative force-directed graph visualizations of core/periphery labeled nodes per age group DF core (dark green; *n* = 46, 79, 73, respectively), DF periphery (blue; *n* = 195, 165, 116, respectively), (bottom) VF core (purple; *n* = 21, 22, 22, respectively), and VF periphery (yellow; *n* = 53, 66, 63) regions.

During cortical development *in vivo*, networks transition from diffuse synchrony to hierarchical core-periphery organization (Bassett et al., 2013). We applied *k*-core decomposition to assess whether organoids exhibit similar specialization (Lahav et al., 2016) (Figure 5A).

Core-periphery connectivity showed no differences at early stages (days 23–33: DF = 0.17 ± 0.02; VF = 0.13 ± 0.03; *p* = 0.54). However, DF organoids exhibited significantly higher core-periphery interaction at intermediate (DF = 0.17 ± 0.01; VF = 0.10 ± 0.03; *p* < 0.001) and late stages (DF = 0.18 ± 0.01; VF = 0.08 ± 0.02; *p* < 0.001; Figure 5B). DF networks maintain globally integrated architecture, while VF networks progressively adopt segregated, modular structures (Figure 5C). This parallels *in vivo* observations where inhibitory maturation drives functional specialization (Wu et al., 2024), suggesting IN enrichment enables progression toward mature cortical organization with functionally distinct communities.

### DF and VF organoids develop distinct hub-based organization

**Figure 6:**
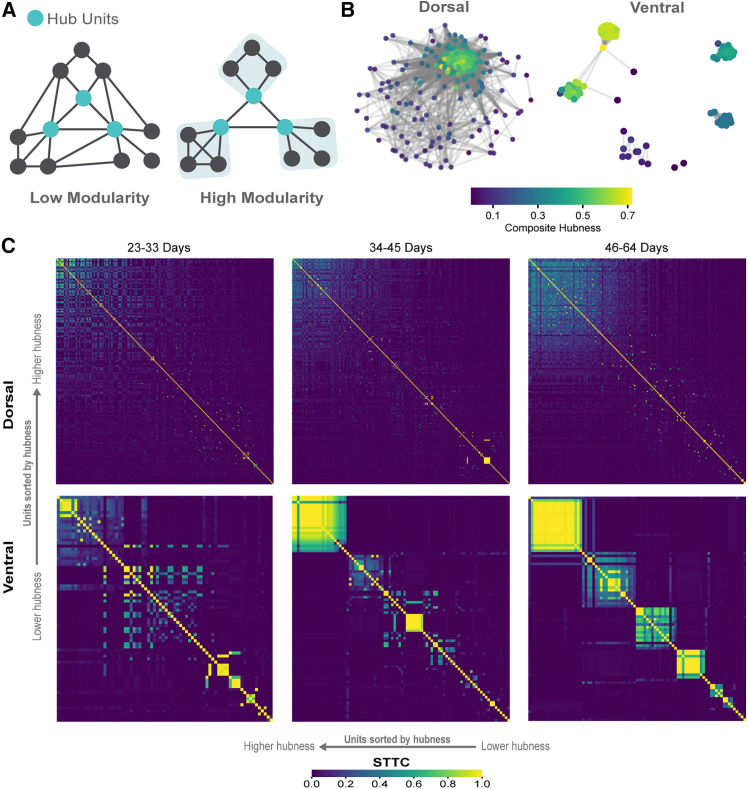
Network modularity dynamics distinguish dorsal and ventral forebrain organoid development (A) Schematics illustrating network modularity, comparing low and high modularity states and highlighting the role of high-hub units. (B) Comparison of examples between DF (*n* = 194 units) and VF (*n* = 87 units) forebrain organoids at the mature stage (46–64 days). (C) STTC matrix of units sorted by hubness score. See also Figures S12–S14; Table S11.

Hub neurons with disproportionate connectivity coordinate population activity during development (Bollmann et al., 2023; Mòdol et al., 2017). To investigate whether a similar phenomenon occurs in organoids, we calculated composite hubness scores incorporating degree, strength, betweenness, and closeness centrality (Figure 6A).

DF organoids formed densely interconnected networks with distributed hubs. VF organoids developed segregated clusters with localized hubs (Figures 6B, S12, and S13). STTC matrices sorted by hubness revealed broadly distributed synchrony in DF organoids versus spatially cohesive hub clusters in VF organoids (Figures 6C, S12, and S13). VF clusters emerged early and became more refined by late development, coinciding with increased modularity (23–33 days: *p* = 0.298; 34–45 days: *p* = 0.007; 46–64 days: *p* = 0.002; Figure S14; Table S11). The spatially refined hub organization in VF organoids is consistent with *in vivo* principles where inhibitory neurons are associated with modular specialization (Hensch, 2005; Contractor et al., 2021).

### Distinct core-periphery dynamics underpin developmental specialization in DF and VF organoids

*In vivo*, developing networks exhibit sequential activation patterns that refine as inhibitory circuits mature (Chini et al., 2022; Wu et al., 2024). We analyzed “*backbone”* units (neurons firing at least twice during 90% of network bursts) that form stable cores supporting sequential activity (van der Molen et al., 2026). Backbone unit proportions showed no early (*p* = 0.86) or intermediate stage differences (*p* = 0.16). By late stages, DF organoids showed significantly higher rigidity (*p* < 0.001; Figure S15; Table S12).

**Figure 7:**
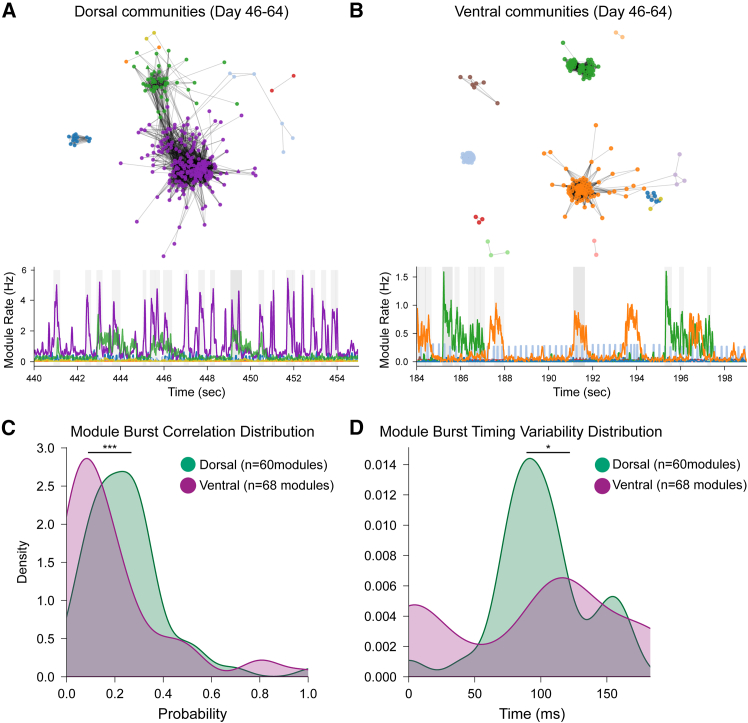
Functional community structure reveals differences in network organization between DF and VF organoids (A) DF organoids (46–64 days) exhibit a densely integrated community structure. Top: force-directed graph of the STTC-derived network, with node colors indicating module membership. Bottom: representative time-series showing synchronized activity across modules; module 4 (green) and module 6 (red) display highly correlated bursts. *n* = 7 modules. (B) VF organoids at the same stage show a more segregated structure. Top: network visualization reveals reduced inter-module connectivity. Bottom: module activity patterns show distinct temporal signatures with less correlation between different functional communities. *n* = 10 modules. (C) Distribution of module burst correlations shows a higher probability of synchronized bursting in DF (green; *n* = 16 organoids) compared to VF (purple; *n* = 18 organoids) organoids. (D) Distribution of burst timing variability indicates broader temporal spread in VF (purple; *n* = 18 organoids) modules, while DF (green; *n* = 16 organoids) modules exhibit tighter, more synchronized timing. ^∗^*p* < 0.05, ^∗∗∗^*p* < 0.0001, Kolmogorov-Smirnov test. See also Figure S15; Tables S12 and S13.

Louvain community detection (Schuurman and Bruner, 2024) revealed DF organoids exhibited higher burst-to-burst correlation (DF = 0.24 ± 0.01; VF = 0.19 ± 0.01; *p* = 0.001) and more regular temporal structure (DF = 95.2 ± 0.9 ms; VF = 94.0 ± 1.4 ms; *p* = 0.01), indicating stable ensemble recruitment (Figure 7; Table S13). VF organoids showed distributed, variable patterns. These organizational differences reflect complementary computational strategies. The stable patterns in DF organoids resemble early developmental states with dominant excitatory drive (Chini et al., 2022), while the variable dynamics in VF organoids parallel observations where elevated inhibitory tone enables flexible ensemble recruitment.

## Discussion

Our study shows that mouse forebrain organoids can self-organize into complex neural networks that recapitulate several organizational principles of cortical development. By generating regionalized DF and VF organoids with distinct cellular compositions, we systematically evaluated how E-I balance shapes network dynamics and topology. Both organoid types contain similar neuronal subtypes at different ratios, with VF organoids enriched in Pvalb^+^ INs. The emergence of small-world architecture in both models indicates that intrinsic developmental programs are sufficient to assemble complex network architectures even without sensory input (Chini et al., 2024; Hilgetag and Kaiser, 2004).

The progressive synchronization in DF organoids contrasts with *in vivo* decorrelation (Chini et al., 2022; Golshani et al., 2009), yet aligns with predictions from the E-I balance hypothesis, which posits that insufficient inhibitory tone prevents network decorrelation. DF organoids contain sparse IN populations, and our pharmacological and qPCR experiments suggest that GABAergic inhibition constrains synchronization. VF organoids enriched with Pvalb^+^ INs exhibit stable STTC trajectories. Importantly, while the observed differences in network maturation strongly correlate with inhibitory neuron enrichment, establishing causality between specific cell type proportions and these network properties will require future targeted manipulations, such as optogenetic or chemogenetic perturbations. Sustained synchronization in human cortical organoids (Mancinelli et al., 2025; Osaki et al., 2024; Trujillo et al., 2019; van der Molen et al., 2026) suggests this reflects a general constraint of systems with limited IN content. While neither model fully recapitulates *in vivo* decorrelation, likely requiring sensory inputs or long-range connectivity (Antón-Bolaños et al., 2018; Dorrn et al., 2010), the divergent trajectories reveal how cellular composition shapes network maturation independently of external cues. Comparable developmental trajectories of synchrony and desynchronization have been documented *ex vivo* using brain slices (Wu et al., 2024), providing a reference framework for interpreting organoid network maturation.

To contextualize these findings, we note that *in vivo* mouse neocortex contains approximately 15%–20% GABAergic interneurons, with Pvalb^+^ cells comprising roughly 40% of this population (Hensch, 2005). Our DF organoids, with sparse interneuron content, fall below this physiological range, while VF organoids likely exceed it. This positions *in vivo* neocortex between our two model systems along the E-I balance spectrum, supporting our interpretation that DF and VF organoids bracket the physiological state and provide complementary platforms for mechanistic investigation.

By establishing reproducible protocols across three genetically distinct mESC lines, we provide a platform to investigate how cellular composition governs circuit assembly. Because DF and VF organoids represent two points along a spectrum of neocortical E-I balance, organoids derived from mouse models of neurodevelopmental disorders with disrupted E-I balance would likely exhibit network topologies intermediate between these extremes, providing testable predictions that can be validated *in vivo* using genetically identical controls. While patient-derived iPSC organoids link to clinical phenotypes, mouse organoids uniquely enable mechanistic testing through causal perturbations, longitudinal recordings, and direct comparison to circuit and behavioral data from mice derived from the same mESC lines. Key organizational principles appear conserved across mammals (Bassett and Bullmore, 2017), though developmental timescales and IN composition differ between species (Krienen et al., 2020). In humans, understanding early network development is primarily inferred from neonatal EEGs showing emerging oscillatory patterns (Chini et al., 2022), but regional comparisons at cellular resolution remain inaccessible due to technical and ethical constraints. Forebrain organoids thus provide a tractable system to rigorously test hypotheses against well-defined *in vivo* benchmarks.

Several limitations should be considered when interpreting our findings. Although organoids recapitulate core features of network development, they lack essential *in vivo* characteristics such as vascularization and complete cellular diversity, including Vip^+^ INs and microglia that contribute to synaptic pruning (Velasco et al., 2019). Our organoids also contain off-target non-neural cell populations, as revealed by scRNAseq, at proportions comparable to those reported in other mouse organoid systems (Lindhout et al., 2025; Medina-Cano et al., 2022; Sánchez et al., 2024). Planar MEAs primarily record from surface neurons, which may bias our network analyses (Tanveer et al., 2025), and do not fully capture 3D circuit architecture. The self-contained nature of organoids also precludes studying how sensory inputs or long-range projections influence circuit development. Additionally, our qPCR analysis of PN/IN proportions required destructive sampling, preventing direct correlation with electrophysiological measurements at the individual organoid level. Non-destructive molecular profiling methods, such as extracellular vesicle analysis or fluorescent reporter lines, would enable such correlations in future studies. Future work incorporating additional cell types, 3D MEAs or optical recording technologies, and patterned stimulation paradigms will be necessary to investigate input-dependent maturation and more fully model *in vivo* developmental dynamics.

Despite these limitations, our findings establish mouse forebrain organoids as a powerful platform for dissecting how cellular composition governs cortical network assembly. By showing that E-I balance fundamentally shapes network topology in the absence of sensory input, we provide a tractable system to investigate the intrinsic developmental logic underlying circuit formation and its disruption in neurodevelopmental disorders.

## Methods

### mESC lines

We used three male mESC lines: BRUCE4 (RRID:CVCL_K037), ES-E14TG2a (RRID:CVCL_Y481), and KH2 (RRID:CVCL_C317). Detailed maintenance protocols are described in the supplemental methods.

### GMEM-based DF organoids generation

DF organoids were generated using a modified version of our previous protocol (Elliott et al., 2023). Full procedures are provided in supplemental methods.

### DMEM-based DF organoids generation

mESCs were dissociated into single cells using TrypLE Express Enzyme (Thermo Fisher Scientific [TFS] #12604021) for 5 min at 37°C, then re-aggregated in Lipidure-coated 96-well V-bottom plates at 3,000 cells per well in 150 μL of mESC maintenance medium supplemented with 10 μM Rho Kinase Inhibitor Y27632 (Tocris #1254) and 1,000 U/mL Recombinant Mouse Leukemia Inhibitory Factor (LIF) (Millipore Sigma [MS] #ESG1107).

After 24 h, the medium was replaced with forebrain patterning medium consisting of Dulbecco's Modified Eagle Medium/Nutrient Mixture F-12 (DMEM/F12) with GlutaMAX (TFS #10565018), 1X chemically defined (CD) lipid concentrate (TFS #11905031), 0.1 mM MEM non-essential amino acids (NEAAs; TFS #11140050), 1 mM sodium pyruvate (MS #S8636), 1X N2 supplement (TFS #17502048), and 2X B27 minus vitamin A (-VitA; TFS #12587010). Supplements included 10 μM Y27632, 5 μM XAV939 (StemCell Technologies (SCT) #1001052), and 5 μM SB431542 (Tocris #1614). Medium was changed daily, with N2 and B27 added post-filtration.

On day 5, organoids were transferred to ultralow adhesion plates (MS #CLS3471) with neuronal differentiation medium and placed on an orbital shaker at 68 rpm.

From days 6–12, progenitor expansion medium included a 1:1 mix of Neurobasal-A and BrainPhys media (SCT #05790), supplemented with B27 -VitA, N2 supplement, MEM NEAAs, CD lipid concentrate, and 200 μM ascorbic acid (Sigma Aldrich [SA] #49752). Medium was changed every 2–3 days under 5% CO_2_.

From day 13 onward, neural maturation medium consisted of BrainPhys medium supplemented with B27 Plus (TFS #A3582801), CD lipid concentrate, and 5 μg/mL heparin (SA #H3149). Ascorbic acid was included until day 25. Medium was changed every 2–3 days, with organoids maintained at 60 rpm (16 per well) to minimize fusion. Primocin (0.05 mg/mL; InvivoGen #antpm05) was included in all media throughout the protocol.

### VF organoids generation

VF organoids were generated using the same protocol as DF organoids with the following modifications: 250 nM LDN193189 (SCT #72147) was added from days 0–5, and 100 nM PD0325901 (SCT #72184) and 100 nM SAG (MS #SIAL-SML1314) were included from days 0–14.

### scRNAseq

Organoids were dissociated using the Worthington Papain Dissociation System (#LK003150) following manufacturer instructions. Libraries were prepared using the PIPseq T2 Single Cell RNA v4.0PLUS platform (Fluent #FBSSCRT28V4.05) or Illumina Single Cell 3′ RNA Prep, T2 (Illumina #20135689), pooling 3,333 cells per genotype (10,000 total per library).

Sequencing was performed on an AVITI PE75 flowcell. Data were processed with PIPseeker, aligned to GRCm39, and analyzed in Seurat using standard quality control, batch correction, clustering, and annotation based on reference brain atlases. Full protocols are provided in supplemental methods.

### IHC and imaging

For detailed IHC conditions, table of antibodies, and additional analysis information, see supplemental methods. Imaging was performed using either: Zeiss 880 confocal microscope with Airyscan Fast or Zeiss AxioImager Z2 widefield microscope, with acquisition via Zen Blue software and analysis in Zen Black/ImageJ.

### Electrophysiology

Day 25 organoids were plated on MaxOne MEAs (Maxwell Biosystems #PSM). Neuronal activity was recorded every 2–3 days at 20 kHz from the 1,020 most active electrodes under standard incubation conditions (5% CO_2_, 37°C). Recordings were filtered, spike-sorted with Kilosort2, and quality controlled. For pharmacology, activity in 60- to 65-day-old DF organoids was recorded before and after treatment with Gabazine (1 μM), NBQX (20 μM), or APV (100 μM). Full methods are detailed in the supplemental methods.

### Quantifications and statistical analyses

Statistical analyses were performed in Python. Details on statistical tests, sample sizes, and *p* values are provided in the figure legends. Significance was defined as *p* < 0.05, with corrections for multiple comparisons applied when appropriate.

### Spike train and network analysis

To quantify neuronal synchrony and population-level organization, we computed pairwise STTC using a 10 ms window and constructed functional connectivity matrices based on significance thresholds derived from spike-shuffled surrogate data. Binary networks were analyzed for clustering, path length, small-worldness, and hub metrics using custom Python pipelines. Full computational methods, including STTC definition, surrogate-based thresholding, and network metrics, are provided in the supplemental methods.

## Resource availability

### Lead contact

Requests for further information and resources should be directed to and will be fulfilled by the lead contact, Mohammed A. Mostajo-Radji (mmostajo@ucsc.edu).

### Materials availability

This study did not generate new reagents.

### Data and code availability

The accesion numbers for the scRNAseq data reported in this paper is GEO: GSE290330, GSE312396, and the scRNAseq data is also available in the UCSC Cell Browser: https://mouse-df-vf-organoid.cells.ucsc.edu, The accession number for the MEA data is DANDI: 001374. All the code is available in GitHub: https://github.com/braingeneers/Sakura_final.

## Acknowledgments

We thank the Colquitt lab for support with scRNA-seq library preparation, Kristof Tigyi for sharing mESC lines, and Tomasz Nowakowski for valuable manuscript feedback. We acknowledge the National Research Platform (NSF CNS1730158, ACI1540112, ACI1541349, OAC1826967), the University of California Office of the President (UCOP), and the UCSD Calit2/Qualcomm Institute. Sequencing was performed at the UC Davis Genome Center (RRID: SCR_012480, NIH 1S10OD010786). Imaging was provided by the UCSC Microscopy Core (RRID: SCR_021135).

This work was supported by 10.13039/100027426Schmidt Futures (SF857 to S.R.S., D.H., and M.T.); the 10.13039/100000051National Human Genome Research Institute (RM1HG011543 to S.R.S., D.H., and M.T.); the 10.13039/100000001National Science Foundation (NSF; 2134955 to S.R.S., D.H., and M.T.; 2034037 to M.T.; 2515389 to T.S., D.H., M.T., and M.A.M.-R.); the 10.13039/100000025National Institute of Mental Health (U24MH132628 to D.H. and M.A.M.-R.); the 10.13039/100000065National Institute of Neurological Disorders and Stroke (U24NS146314 to D.H. and M.A.M.-R.); the 10.13039/100000900California Institute for Regenerative Medicine (CIRM) (DISC4-16285 to S.R.S., M.T., and M.A.M.-R.; DISC4-16337 to M.A.M.-R.); UCOP(M25PR9045 to S.R.S., M.T., and M.A.M.-R.); and the 10.13039/100000874Brain and Behavior Research Foundation (33184 to M.A.M.-R.). H.E.S. was supported by NSF 10.13039/100023581Graduate Research Fellowship Program (GRFP), S.H. by University of California - Hispanic Serving Institutions Doctoral Diversity Initiative (UC-HSI DDI), and F.R. by CIRM Bridges Program. The content is solely the responsibility of the authors and does not necessarily represent the official views of the National Institutes of Health, the National Science Foundation, the University of California, CIRM, or any other agency of the State of California.

## Author contributions

S.H., H.E.S., M.T., and M.A.M.-R. conceptualized the project; S.H., H.E.S., I.C., G.A.K., A.R., D.S., S.V.-C., J.G., T.v.d.M., F.R., C.N.A., and K.V. conducted the experiments; M.C., M.R., S.R.S., B.M.C., T.S., D.H., M.T., and M.A.M.-R. provided supervision and secured funding; S.H., H.E.S., and M.A.M.-R. wrote the manuscript with input from all authors.

## Declaration of interests

K.V. is a co-founder, and D.H., S.R.S., and M.T. are advisory board members of Open Culture Science, Inc. A.R. is co-founder and CTO of Immergo Labs. H.E.S. and M.A.M.-R. are inventors on a patent application for brain organoid generation; M.A.M.-R. is also listed on patent applications for electrophysiology analysis and Pvalb^+^ IN generation, and advises Atoll Financial Group and Optimal.

## Declaration of generative AI and AI-assisted technologies in the writing process

During the preparation of this work, the authors used ChatGPT, Gemini, and Claude to improve language clarity and readability. All content was reviewed and edited by the authors, who take full responsibility for the final version.

## Supplemental information

Document S1. Figures S1–S15, Tables S1–S13, and supplemental methods

Document S2. Article plus supplemental information
