# Supplementary material for: Establishing mouse forebrain organoids as models of intrinsic cortical network assembly
Source: Stem Cell Reports. 2026 Mar 5;21(4):102832. doi: 10.1016/j.stemcr.2026.102832 (PMC13083800; doi:10.1016/j.stemcr.2026.102832)
Supplement: Document S1. Figures S1–S15, Tables S1–S13, and supplemental methods [file mmc1.pdf]

Stem Cell Reports, Volume *21*

Supplemental Information

Establishing mouse forebrain organoids as models of intrinsic cortical network assembly

Sebastian Hernandez, Hunter E. Schweiger, Isabel Cline, Gregory A. Kaurala, Ash Robbins, Daniel Solis, Samira Vera-Choqqueccota, Jinghui Geng, Tjitse van der Molen, Francisco Reyes, Chinweike Norman Asogwa, Kateryna Voitiuk, Mattia Chini, Marco Rolandi, Sofie R. Salama, Bradley M. Colquitt, Tal Sharf, David Haussler, Mircea Teodorescu, and Mohammed A. Mostajo-Radji

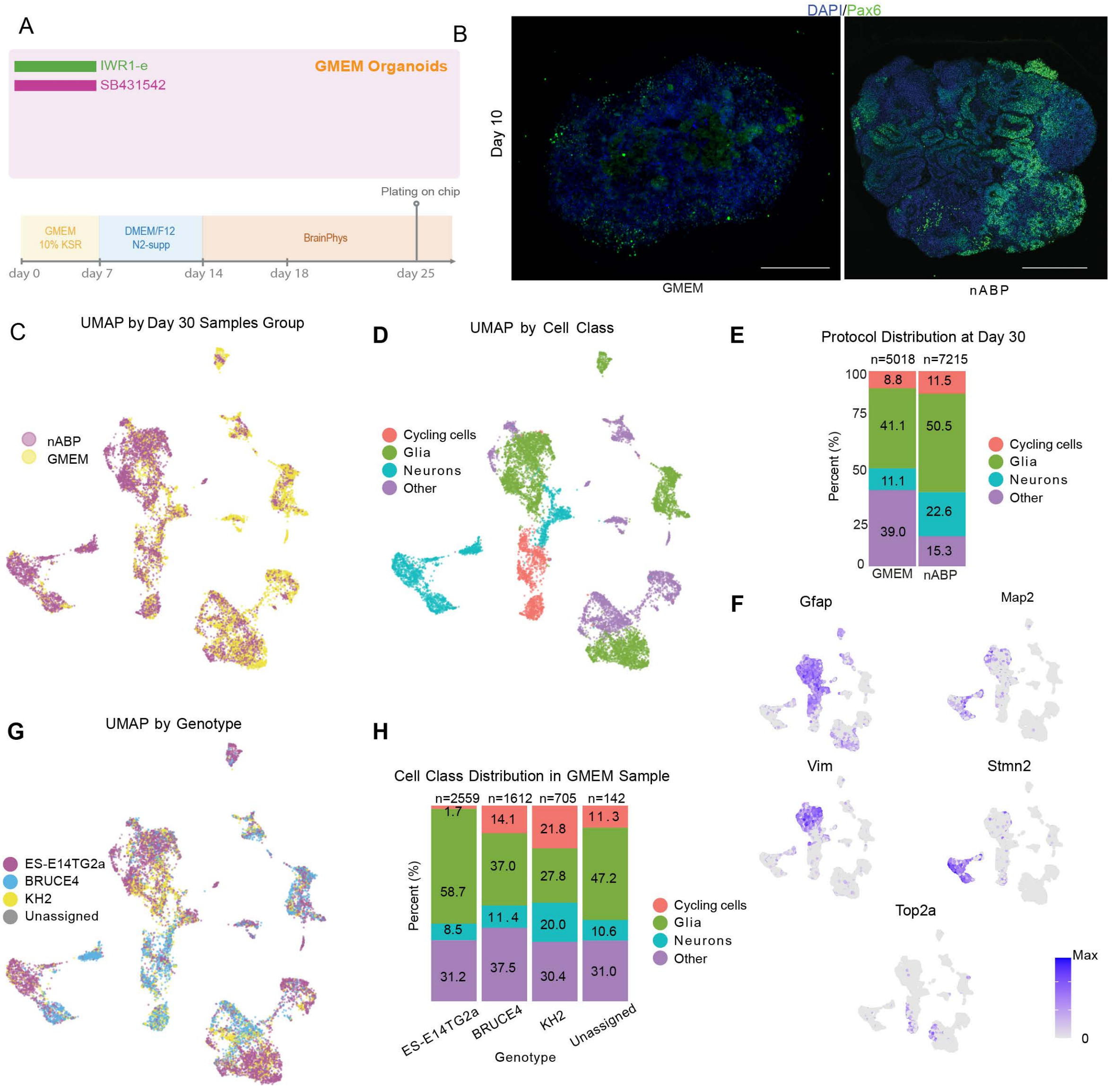

Figure S1: Comparison of GMEM-based and Neurobasal-A/BrainPhys (NABP) protocols in DF organoid development, related to Figure 1.

(A) Schematic of the GMEM-based protocol.

(B) Representative IHC images of day 10 organoids generated using the GMEM-based (left) and nABP (right) protocols, stained for DAPI (blue) and Pax6 (green). Scale bars: 250 µm.

(C) UMAP visualization of single-cell RNA sequencing data from day 30 samples, colored by protocol (NABP and GMEM-based).

(D) UMAP visualization showing cell class distribution (Cycling cells, Glia, Neurons, and Other).

(E) Stacked bar plot comparing cell class distributions between GMEM-based (n = 5,018 cells) and nABP (n = 7,215 cells) protocols at day 30. The NABP protocol shows an increase in neuronal populations and a decrease in off-target "Other" cells.

(F) UMAP plots displaying expression of key marker genes (Gfap, Map2, Vim, Stmn2, and Top2a).

(G) UMAP visualization colored by genotype (ES-E14TG2a, BRUCE4, KH2, and Unassigned).

(H) Detailed cell class distribution across different genotypes in GMEM-based samples. Other indicates off-target cells.

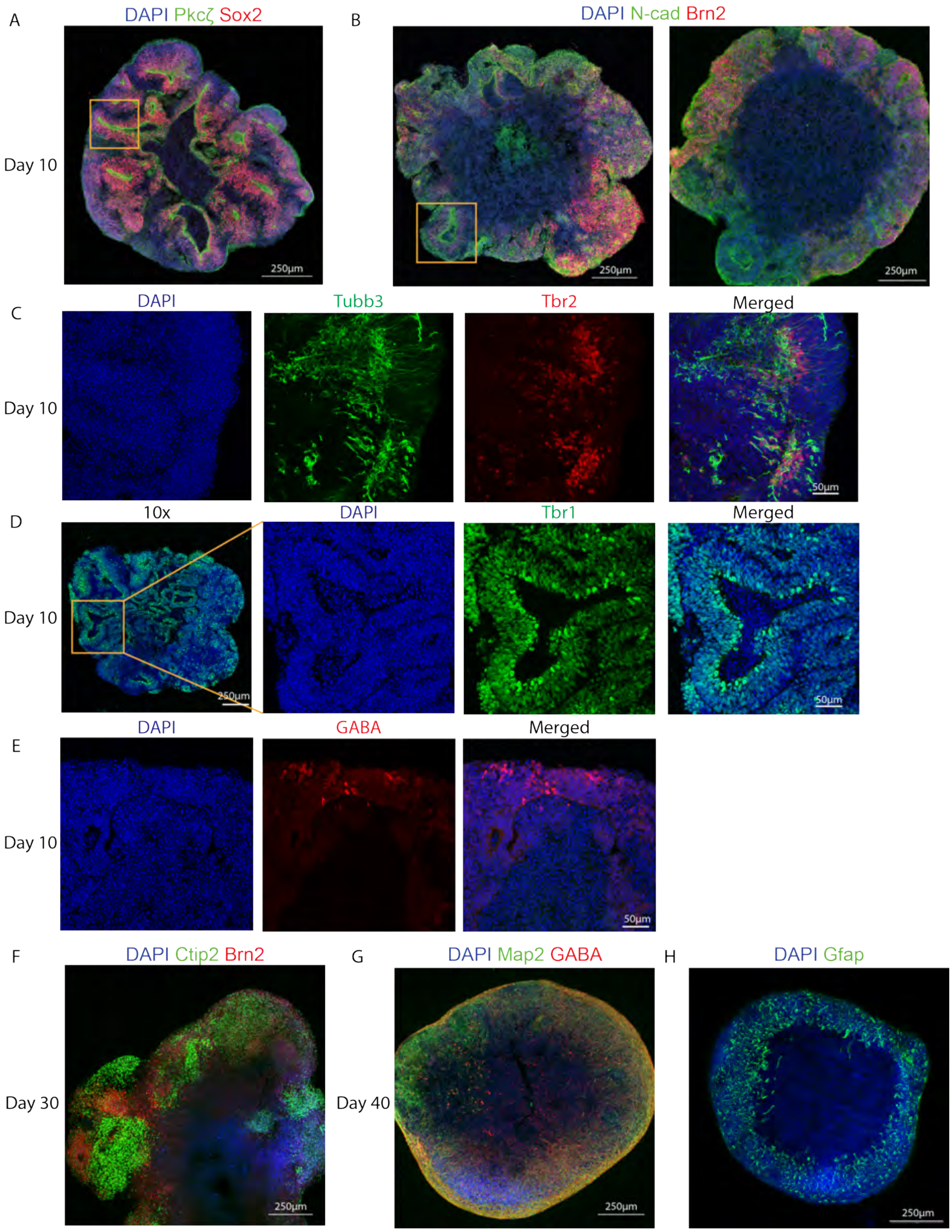
A
DAPI Pkcζ Sox2
Day 10
250µm
B
DAPI N-cad Brn2
250µm
250µm
C
DAPI
Tubb3
Tbr2
Merged
Day 10
50µm
D
10x
DAPI
Tbr1
Merged
Day 10
250µm
50µm
E
DAPI
GABA
Merged
Day 10
50µm
F
DAPI Ctip2 Brn2
Day 30
250µm
G
DAPI Map2 GABA
Day 40
250µm
H
DAPI Gfap
250µm

Figure S2: Immunohistochemical characterization of DF organoids, related to Figure 1.

(A) Low-magnification image of a day 10 organoid stained for Pkcζ (green) and Sox2 (red) (related to figure 1C).
(B) Low-magnification image of a day 10 organoid stained for N-cadherin (green) and Brn2 (red) (related to figure 1C).
(C) IHC of a day 10 DF organoid stained for Tubb3 (green) and Tbr2 (red), with merged image.
(D) IHC of a day 10 organoid. (left) low-magnification overview. (right) high-magnification view of Tbr1 (green).
(E) IHC of a day 10 organoid stained for GABA (red).
(F) Low-magnification image of a day 30 organoid stained for Ctip2 (green) and Brn2 (red) (corresponding to Figure 1D).
(G) IHC of a day 40 organoid stained for Map2 (green) and GABA (red).
(H) IHC of a day 40 organoid stained for Gfap (green).
Scale bars as indicated (50 or 250 μm). DAPI nuclear counterstain shown in blue.

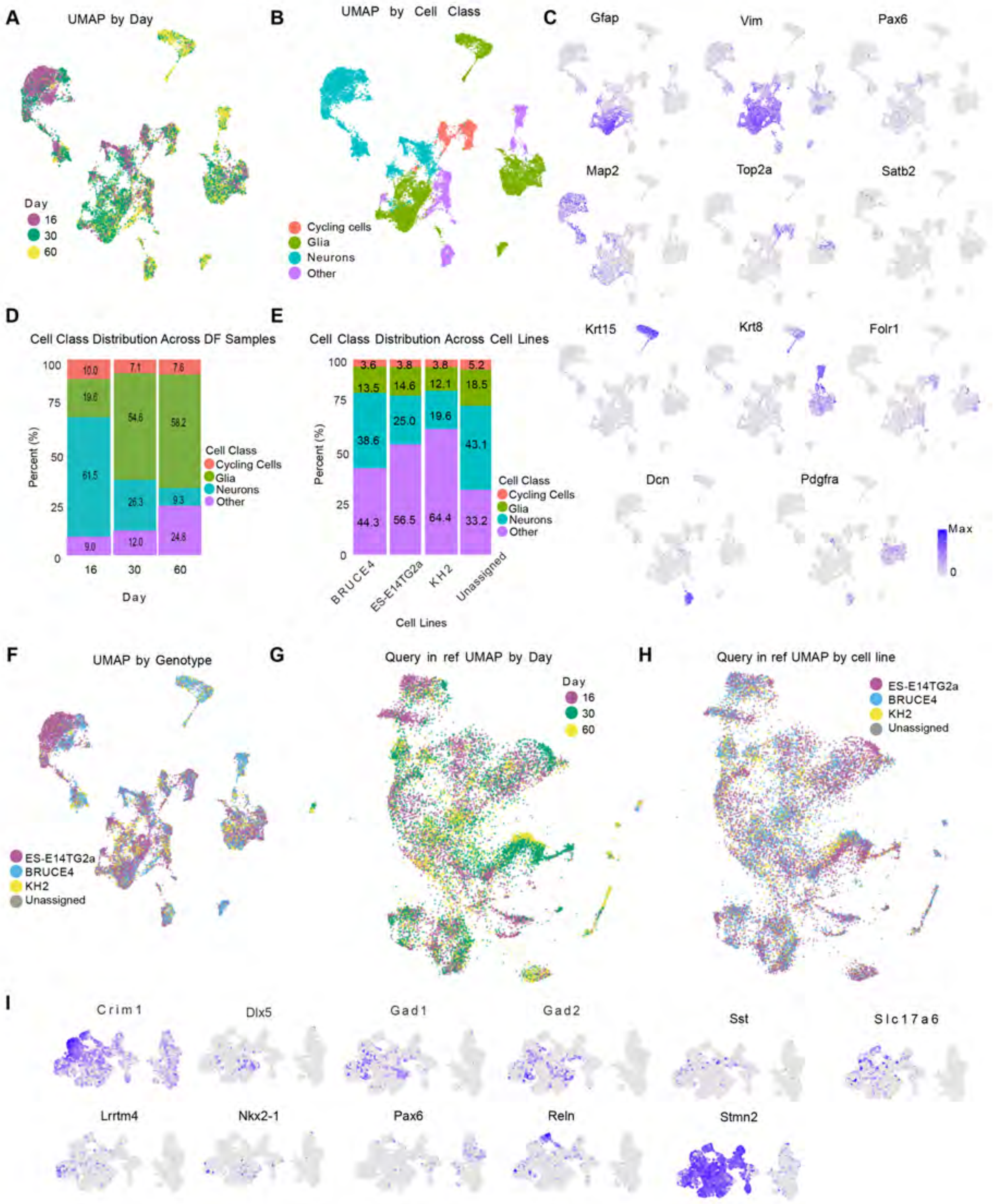
A
UMAP by Day
Day
16
30
60
B
UMAP by Cell Class
Cycling cells
Glia
Neurons
Other
C
Gfap
Vim
Pax6
Map2
Top2a
Satb2
Krt15
Krt8
Folr1
Dcn
Pdgfra
Max
0
D
Cell Class Distribution Across DF Samples
Percent (%)
Day
16
30
60
Cell Class
Cycling Cells
Glia
Neurons
Other
E
Cell Class Distribution Across Cell Lines
Percent (%)
Cell Lines
BRUCE4
ES-E14TG2a
KH2
Unassigned
F
UMAP by Genotype
ES-E14TG2a
BRUCE4
KH2
Unassigned
G
Query in ref UMAP by Day
H
Query in ref UMAP by cell line
I
Crim1
Dlx5
Gad1
Gad2
Sst
Slc17a6
Lrrtm4
Nkx2-1
Pax6
Reln
Stmn2

Figure S3: Developmental progression and cellular composition of NABP protocol DF organoids, related to Figure 1.
(A) UMAP by time point visualization of dorsal forebrain cells (days 16, 30, and 60). n = 17,148 cells.
(B) UMAP highlighting major cell classes (Cycling cells, Glia, Neurons, and Other). Other denotes off-target cell types.
(C) Feature plot displaying expression levels of canonical marker genes used for cell type classification (Gfap, Vim, Pax6, Map2, Top2a, Satb2, Krt15, Krt8, Folr1, Dcn, and Pdgfra). Expression intensity is indicated by a color gradient from gray (low) to purple (high) in same UMAP space as A-B.
(D) Stacked bar plot showing the proportional distribution of cell classes at each time-point. Other indicates off-target cells.
(E) Stacked bar plot showing the proportional distribution of cell classes for cell line. Other indicates off-target cells.
(F) UMAP visualization of cells grouped by genotype (ES-E14TG2a, BRUCE4, KH2, and Unassigned).
(G) DF organoid cells mapped into primary reference space colored by timepoint.
(H) Same as G but colored by genotype.
(I) Feature plot displaying expression levels of canonical marker genes used for cell type classification (Crim1, Dlx5, Gad1, Gad2, Sst, Slc17a6, Lrrtm4, Nkx2-1, Pax6, Reln, Stmn2). Expression intensity is indicated by a color gradient from gray (low) to purple (high) in same UMAP space as Figure 1H.

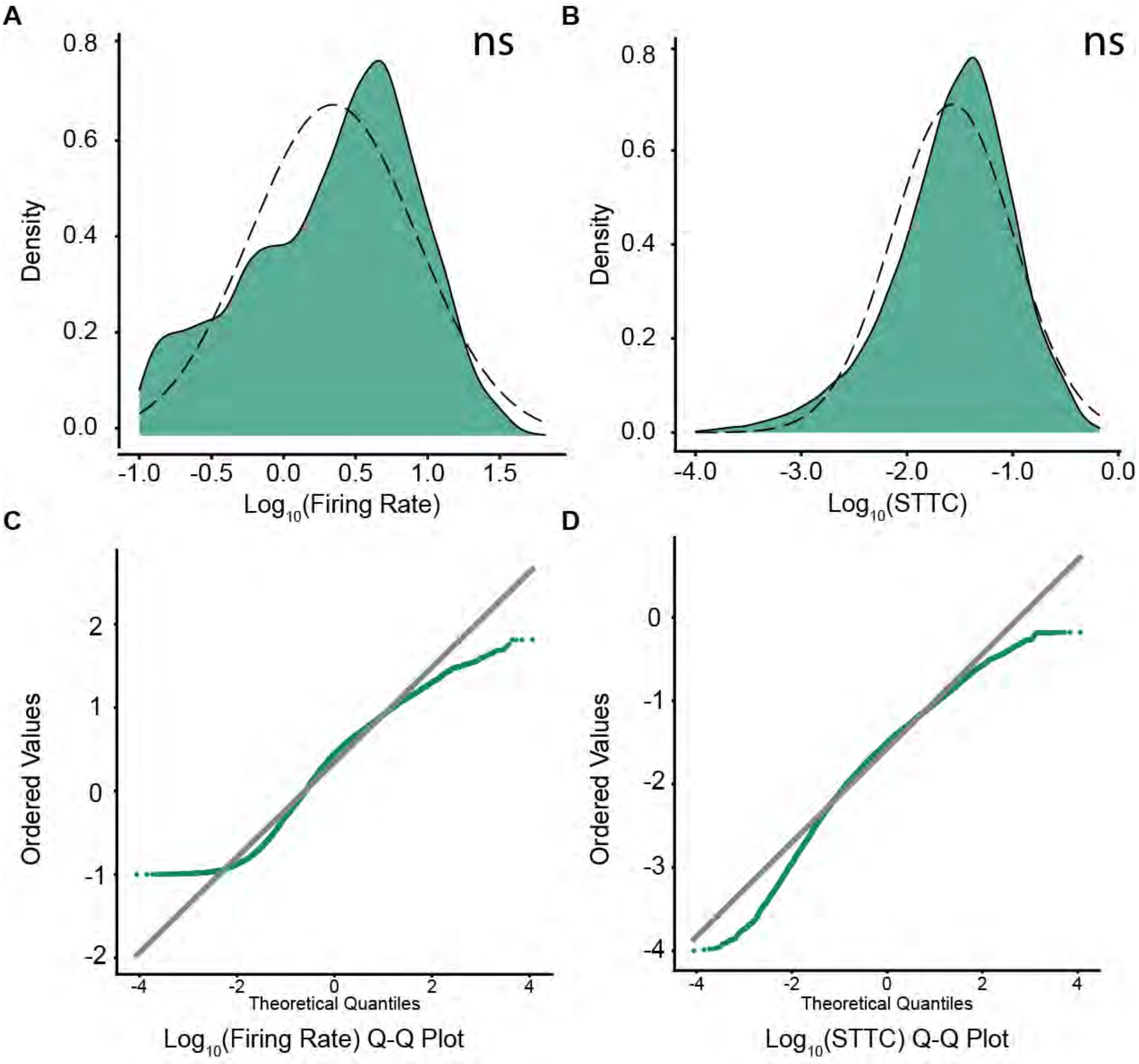

Figure S4: DF and VF organoids exhibit log-normal distributions, related to Figure 2. (A-B) Log-normal distribution of log transformed mean firing rate distribution (A) and log transformed mean STTC (B) (green) with theoretical normal distribution (dashed line). (C-D) Q-Q Plots showing that both log transformed mean firing rate distribution (A) and log transformed mean STTC follow a log-normal distribution.
ns = not significant. Kolmogorov–Smirnov test

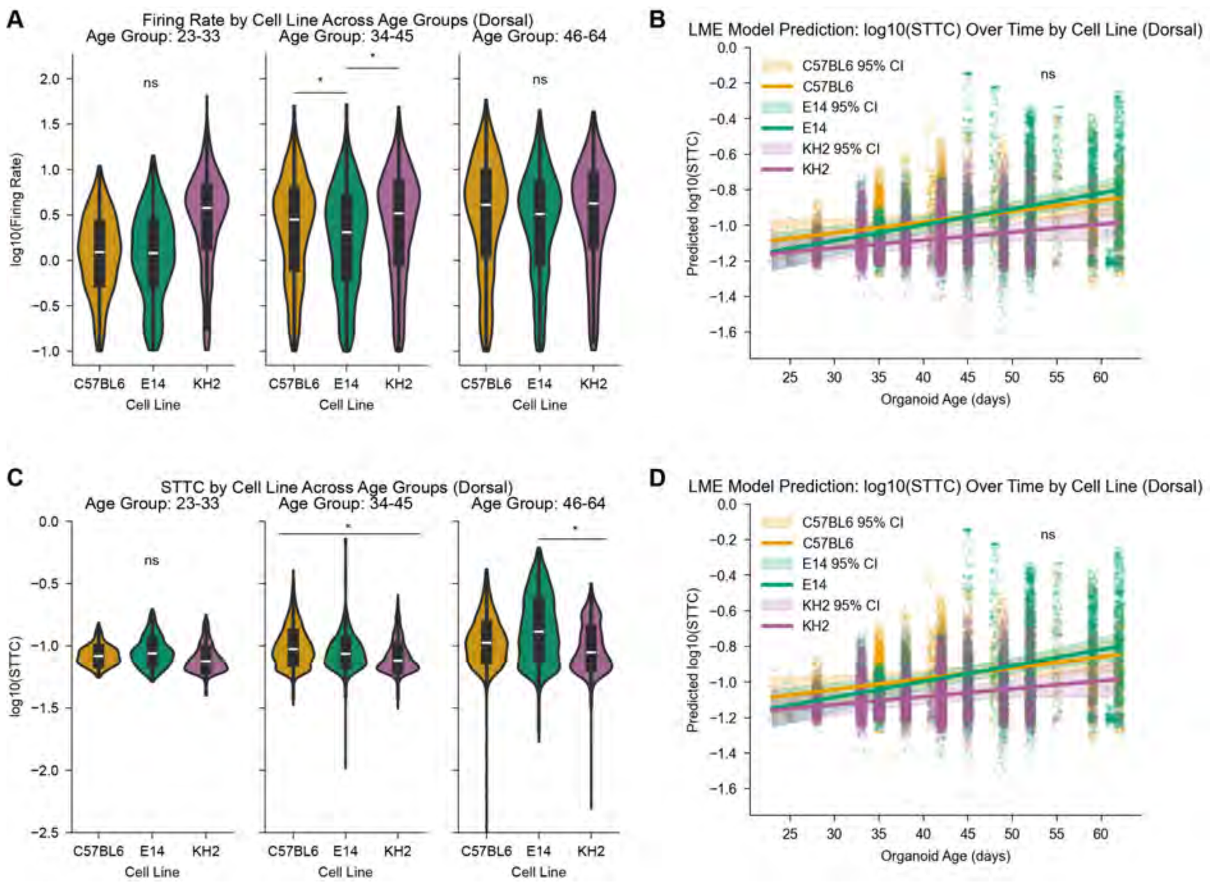

Figure S5: Similar DF electrophysiological distributions across cell lines, related to Figure 2.

(A) Violin plots showing log10(Firing Rate) distributions by cell line (C57BL6, ES-E14TG2a, and KH2) across three age groups (23-33, 34-45, and 46-64) in DF organoids. n = 16 organoids, 28,809 units.

(B) LME model predictions of log10(STTC) over development by cell line for DF organoids. (C) Violin plots showing log10(STTC) distributions by cell line across the same three age groups in DF organoids. (D) LME model prediction of log10(STTC) over development by cell line for DF organoids, similar to panel B but with potentially different parameter settings.

ns = not significant, $p < 0.017$ (Bonferroni corrected). Mixed-effects model

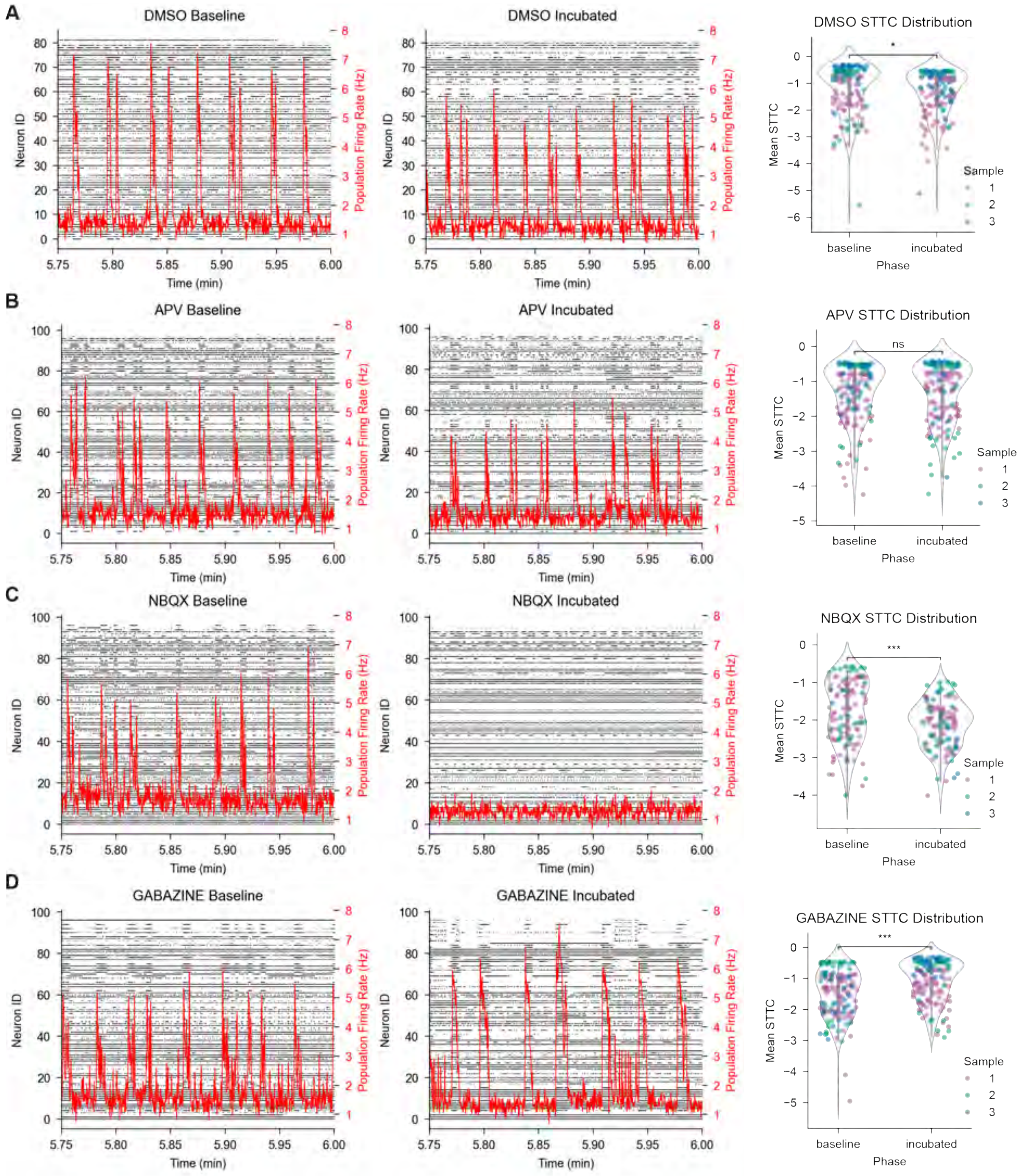

Figure S6: Impact of pharmacological perturbation on overall neural connectivity, related to Figure 2.

(A) Raster plots of neural activity (gray) and population firing rate (red) during baseline (left) and post-drug incubation (middle) for a 15s window in DMSO vehicle control. Each row represents individual unit spike trains. (Right) STTC distribution showing the mean log STTC values across all 3 organoid samples. Organoid 1 is shown in purple (n = 97), organoid 2 (n = 53) in green, and organoid 3 (n = 35) in yellow. Statistical significance indicated by asterisks: * $p < 0.05$, ** $p < 0.001$, *** $p < 0.0001$, ns: not significant.

(B) As in panel A but following treatment with NMDA antagonist APV.

(C) As in panel A but following treatment with AMPA/Kainate antagonist NBQX.

(D) As in panel A but following treatment with $GABA_A$ antagonist Gabazine.

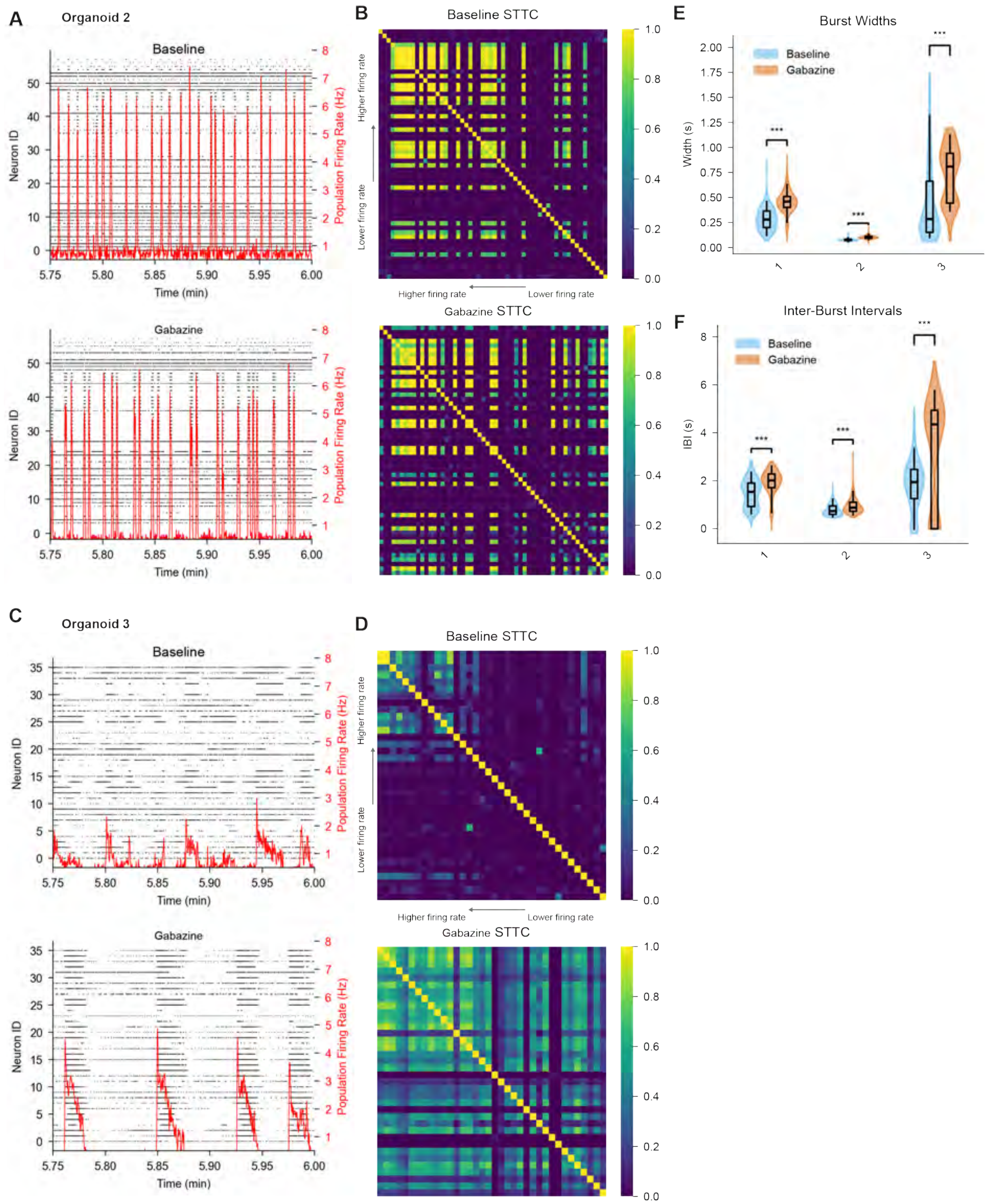

Figure S7: Effects of GABA receptor antagonism on bursting activity in dorsal forebrain organoids, related to Figure 2.

(A) Representative raster plots from Sample 2 showing neural activity (gray) and population firing rate (red) during baseline (left) and post-Gabazine incubation (middle) over a 15s window. Each row represents a single-unit spike train. n = 53

(B) STTC matrices sorted by firing rate (ascending to descending). (left) Baseline STTC matrix. Middle: STTC matrix post-Gabazine incubation. (right) Difference matrix showing STTC changes. n = 53

(C) Same as (A), but for Sample 3. n = 35

(D) Same as (B), but for Sample 3. n = 35

(E) Burst width across three organoids.

(F) Inter-burst interval across three organoids.

(E–F) Statistical comparison of baseline vs. Gabazine conditions. Statistical significance: * $p < 0.05$, ** $p < 0.001$, *** $p < 0.0001$; ns = not significant. Mann–Whitney U test.

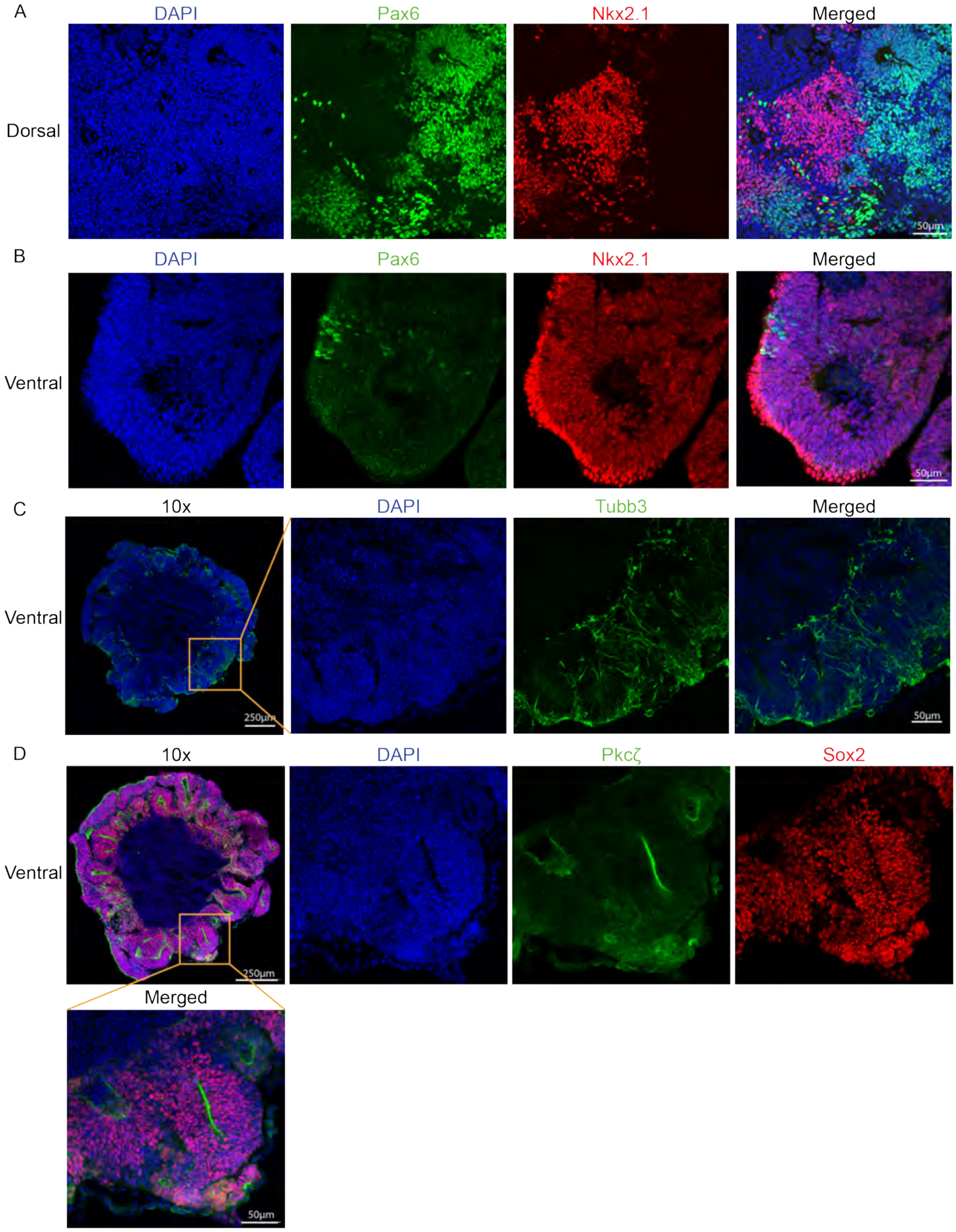

Figure S8: Patterning marker expression in developing DF and VF organoids, related to Figure 3.
(A) High-magnification view of the DF from Figure 3D showing Pax6 (green) and Nkx2.1 (red) staining with the merged image.
(B) High-magnification view of the VF from Figure 3D, showing Pax6 (green) and Nkx2.1 (red) staining with the merged image.
(C) Day 10 VF organoid. (left) Low-magnification overview. (right) High-magnification view of Tubb3 (green) staining with the merged image.
(D) Day 10 VF organoid. (left) Low-magnification overview. (right) High-magnification view of Pkcζ (green) and Sox2 (red).
All panels include DAPI nuclear counterstain (blue), with scale bars as indicated (50 or 250 µm).

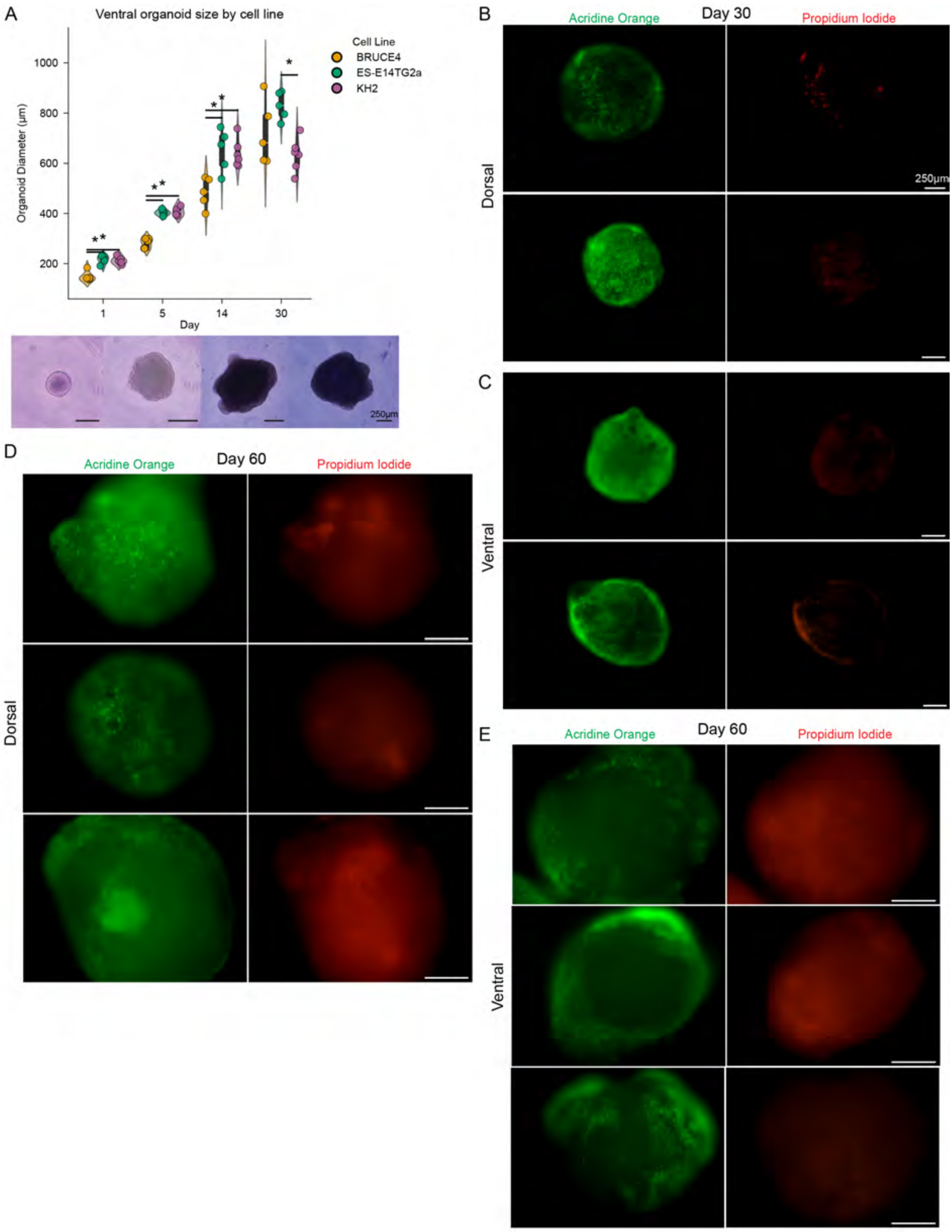

A
Ventral organoid size by cell line
Organoid Diameter (µm)
Day
Cell Line
BRUCE4
ES-E14TG2a
KH2
250µm
B
Acridine Orange
Day 30
Propidium Iodide
Dorsal
C
Ventral
D
Day 60
E

Figure S9: DF and VF organoids maintain viability and similar size over long term development, related to Figure 3.
(A) VF organoid size over time split by cell lines. n = 5 organoids per cell line
(B) Live/dead viability dye in day 30 DF organoids showing acridine orange (green) and propidium iodide (red).
(C) Same as B for day 30 VF organoids
(D) Same as B for day 60 DF organoids
(E) Same as B for day 60 VF organoids
Dunn's post hoc test, * $p < 0.05$.

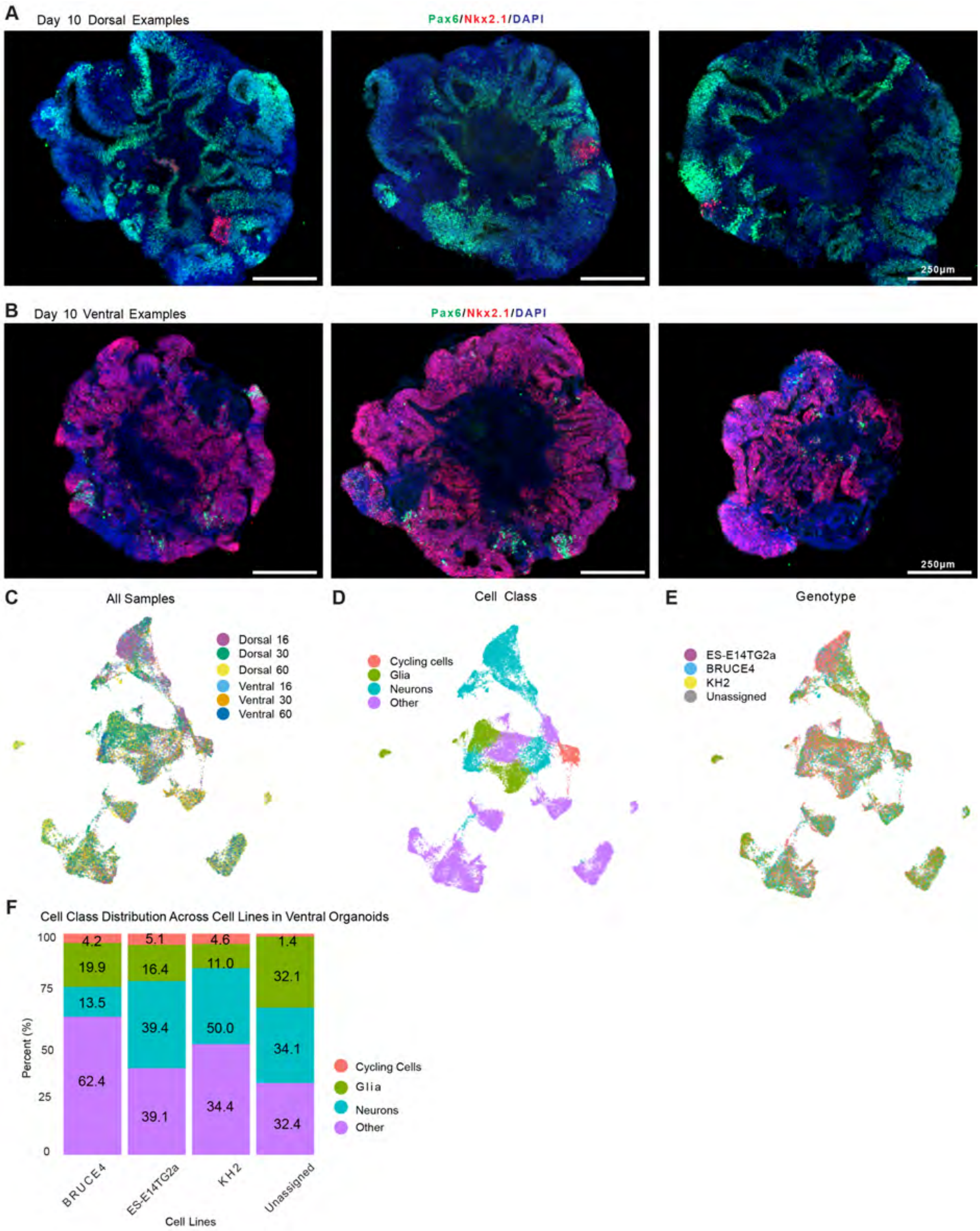

A
Day 10 Dorsal Examples
Pax6/Nkx2.1/DAPI
250μm
B
Day 10 Ventral Examples
Pax6/Nkx2.1/DAPI
250μm
C
All Samples
Dorsal 16
Dorsal 30
Dorsal 60
Ventral 16
Ventral 30
Ventral 60
D
Cell Class
Cycling cells
Glia
Neurons
Other
E
Genotype
ES-E14TG2a
BRUCE4
KH2
Unassigned
F
Cell Class Distribution Across Cell Lines in Ventral Organoids
Percent (%)
100
75
50
25
0
4.2
19.9
13.5
62.4
5.1
16.4
39.4
39.1
4.6
11.0
50.0
34.4
1.4
32.1
34.1
32.4
BRUCE4
ES-E14TG2a
KH2
Unassigned
Cell Lines
Cycling Cells
Glia
Neurons
Other

Figure S10: Characterization of DF and VF organoid development, related to Figure 3.

(A) IHC images of additional day 10 DF organoids stained for Pax6 (green) and Nkx2.1 (red). These organoids were used for quantifications in Figure 3D.
(B) IHC images of additional day 10 VF organoids stained for Pax6 (green) and Nkx2.1 (red). These organoids were used for quantifications in Figure 3D.
(C) UMAP visualization of scRNA-seq data colored by sample type (DF and VF). n = 10,495 cells.
(D) UMAP plot showing cell class (Cycling cells, Glia, Neurons, and Other). Other indicates off-target cells.
(E) UMAP visualization colored by genotype (ES-E14TG2a, BRUCE4, KH2, and Unassigned).
(F) Stacked bar plot showing cell class distribution across genotypes in VF samples. Other indicates off-target cells. Panels (A–B) include DAPI nuclear counterstain (blue) and scale bars as indicated (250 μm).

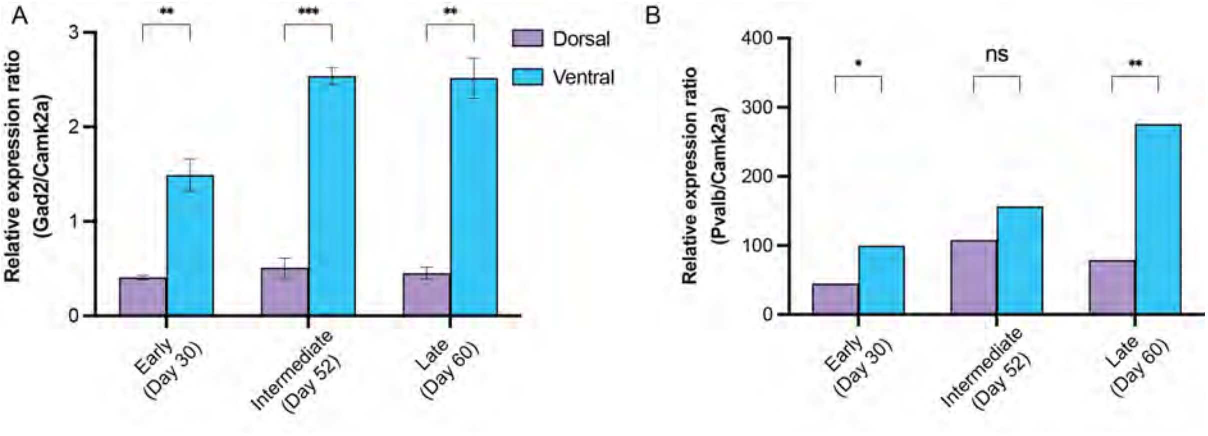

Figure S11: Relative expression of GABAergic and glutamatergic genes, related to Figure 4.
(A) Relative expression ratio of *Gad2* divided by *Camk2a* for timepoints: early (day 30), intermediate (day 52), and late (day 60) n = 3 organoids across 3 separate batches.
(B) Same as (A) but for relative expression ratio of *Pvalb* divided by *Camk2a*.

Core-Periphery, Hubness & development - DF Organoids
(Same STTC chip: through 23124)

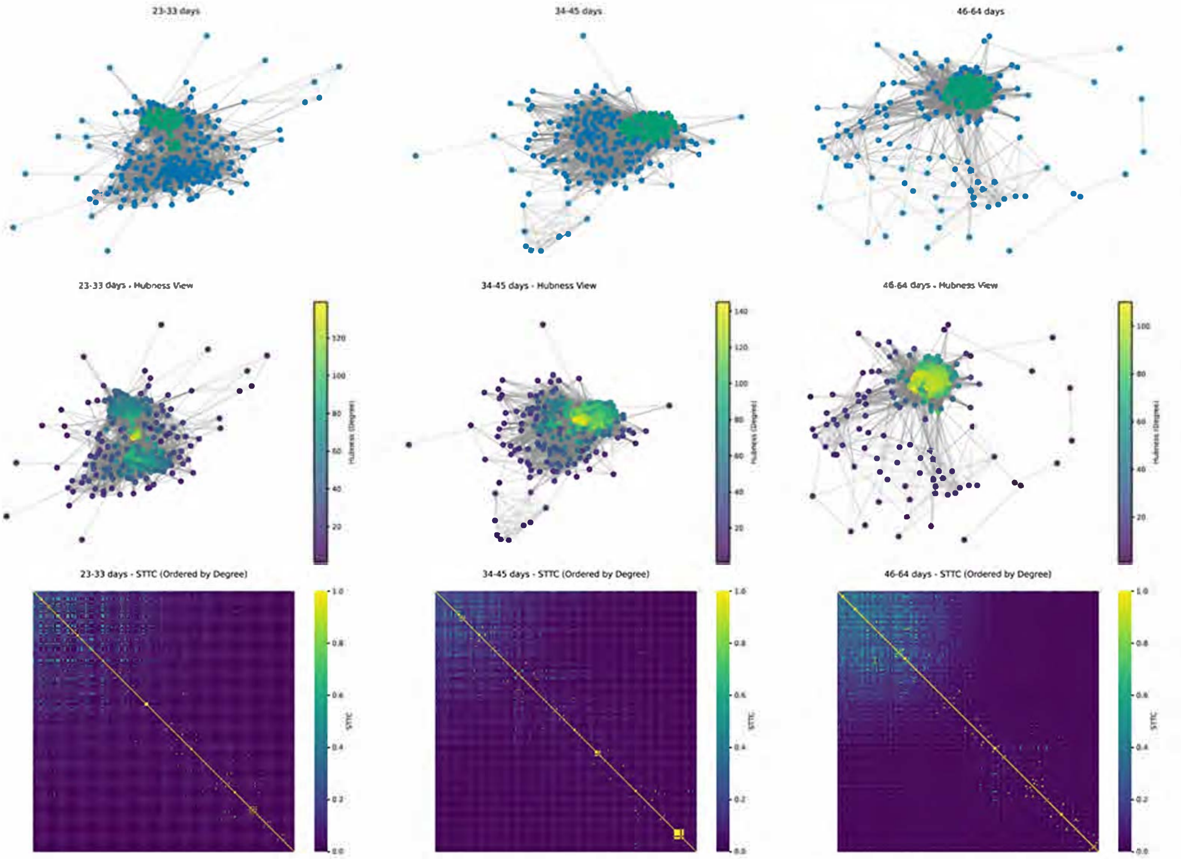

Core-Periphery, Hubness & development - DF Organoids
(Same STTC chip: through 22710)

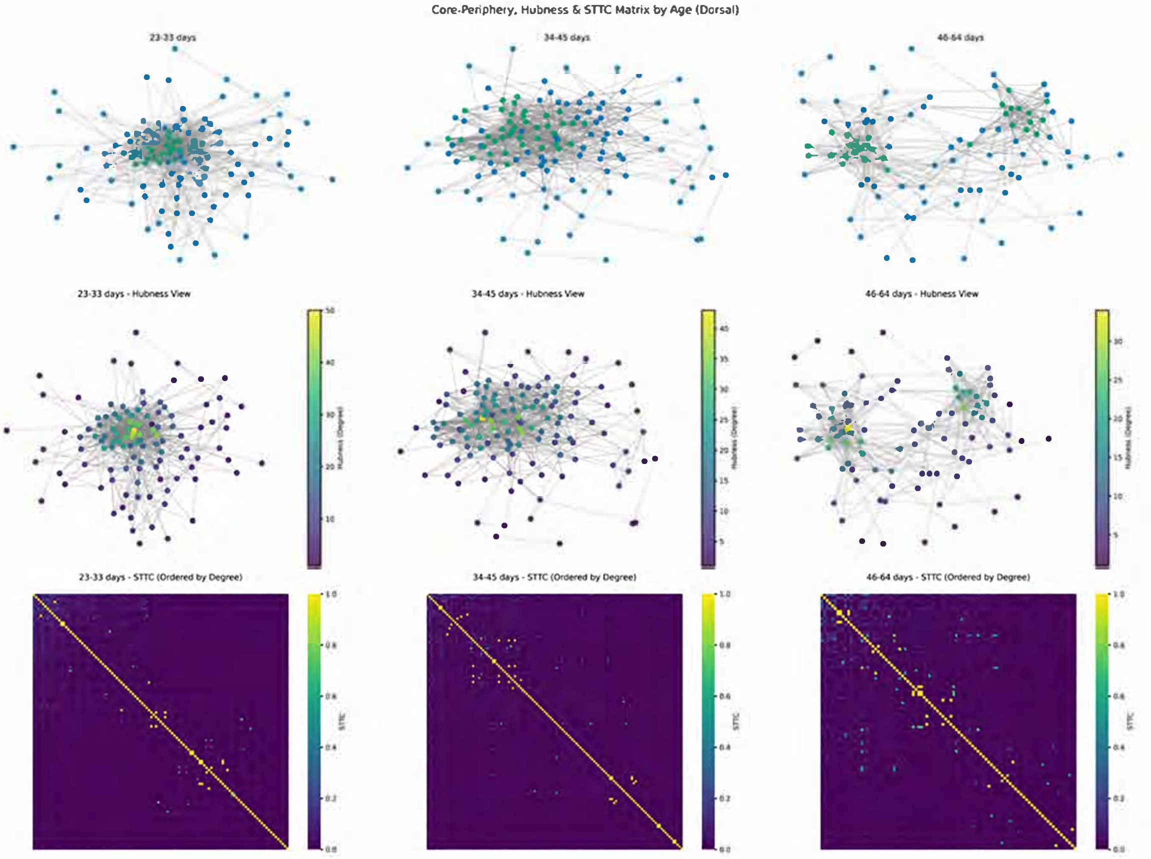

Figure S12: Developmental changes in network connectivity of DF organoids tracked longitudinally, related to Figure 6.
(A) Longitudinal analysis of organoid chip 23120 shown at three developmental time-points (23-33 days, 34-45 days, and 46-64 days). Upper panels display hubness visualizations where node colors represent hubness score. Lower panels show corresponding STTC matrices ordered by connection degree. n = green: 46, 79, 73; blue: 195, 165, 116. units
(B) Longitudinal analysis of organoid chip 23120 shown at three developmental time-points (23-33 days, 34-45 days, and 46-64 days). Upper panels display hubness visualizations where node colors represent hubness score. Lower panels show corresponding STTC matrices ordered by connection degree. green: 21, 39, 28; blue: 90, 90, 67.

Core-Periphery, Hubness & development - VF Organoids
(Same STTC chip: through 25136)

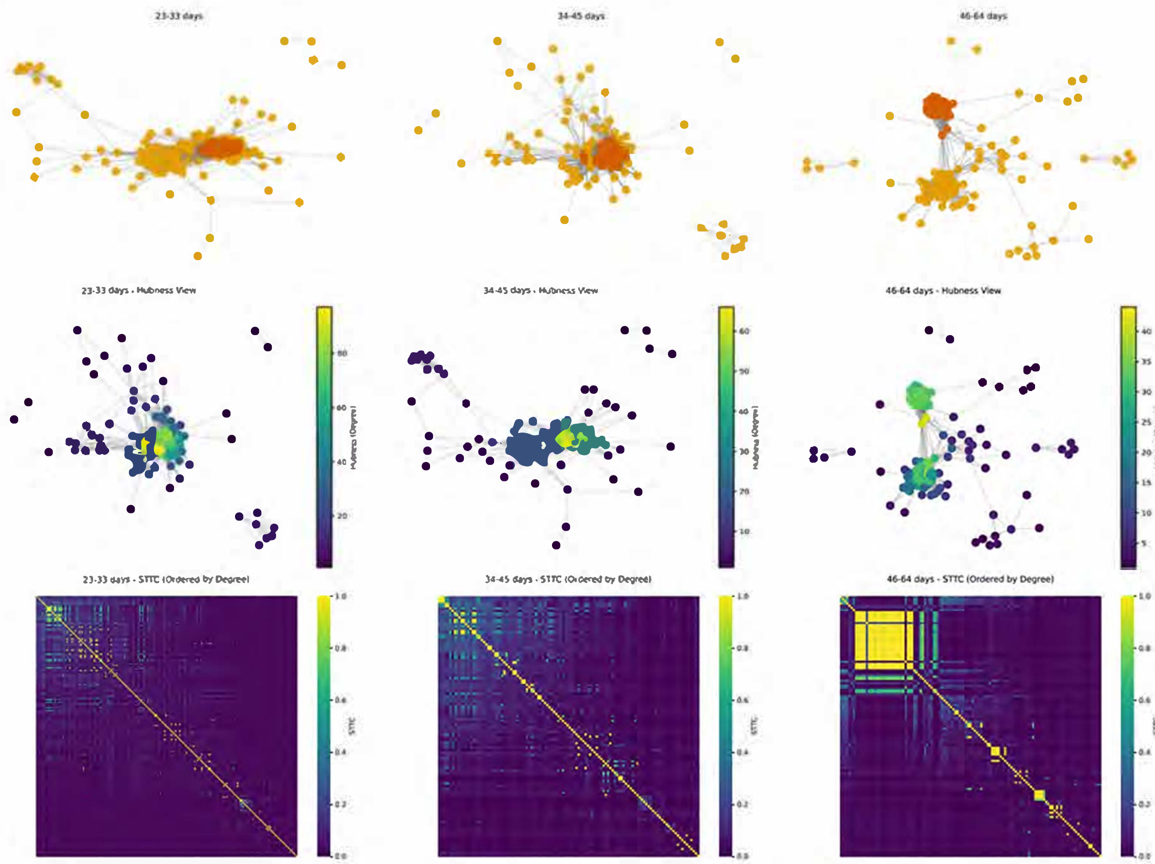

Core-Periphery, Hubness & development - VF Organoids
(Same STTC chip: through 22064b)

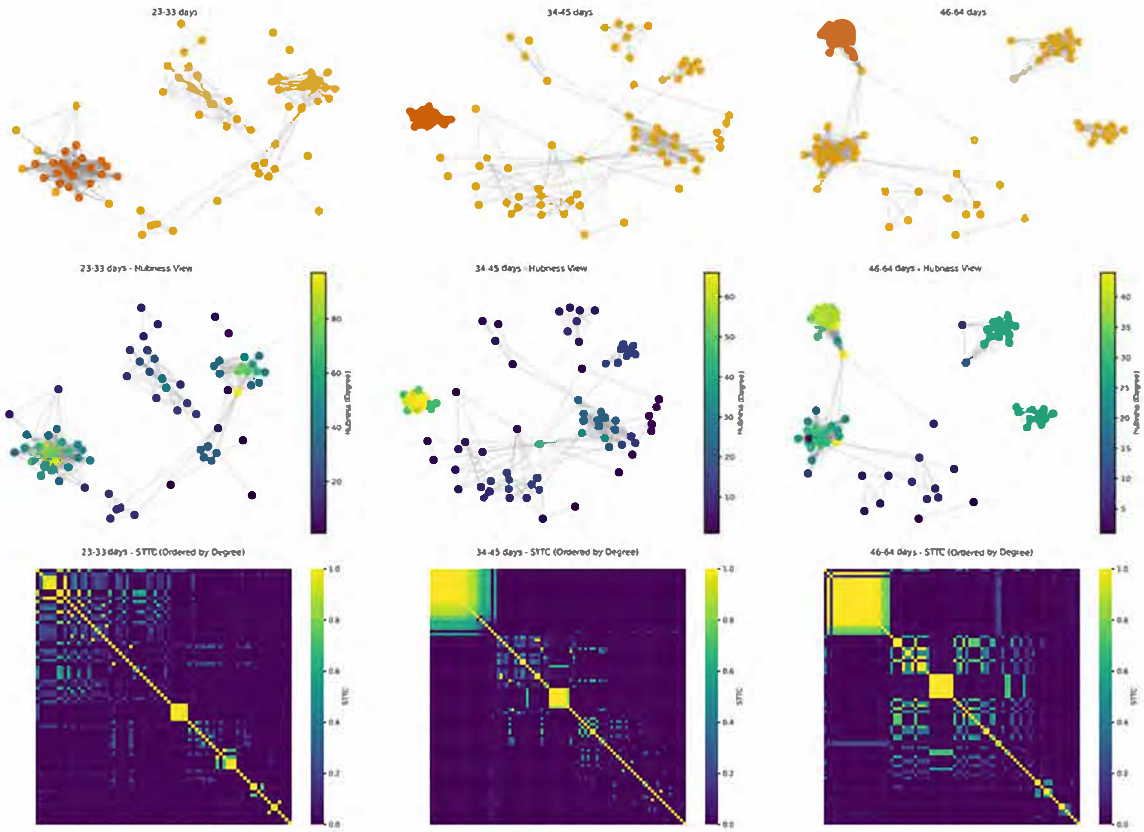

Figure S13: Developmental changes in network connectivity of VF organoids tracked longitudinally, related to Figure 6.
(A) Longitudinal analysis of organoid chip 25136 shown at three developmental time-points (23-33 days, 34-45 days, and 46-64 days). Upper panels display hubness visual-izations where node colors represent hubness score. Lower panels show corresponding STTC matrices ordered by connection degree. n = orange: 58, 39, 35; yellow: 132, 77, 86.
(B) Longitudinal analysis of organoid chip 22064b shown at three developmental time-points (23-33 days, 34-45 days, and 46-64 days). Upper panels display hubness visual-izations where node colors represent hubness score. Lower panels show corresponding STTC matrices ordered by connection degree. n = orange: 21, 22, 22; yellow: 53, 66, 63.

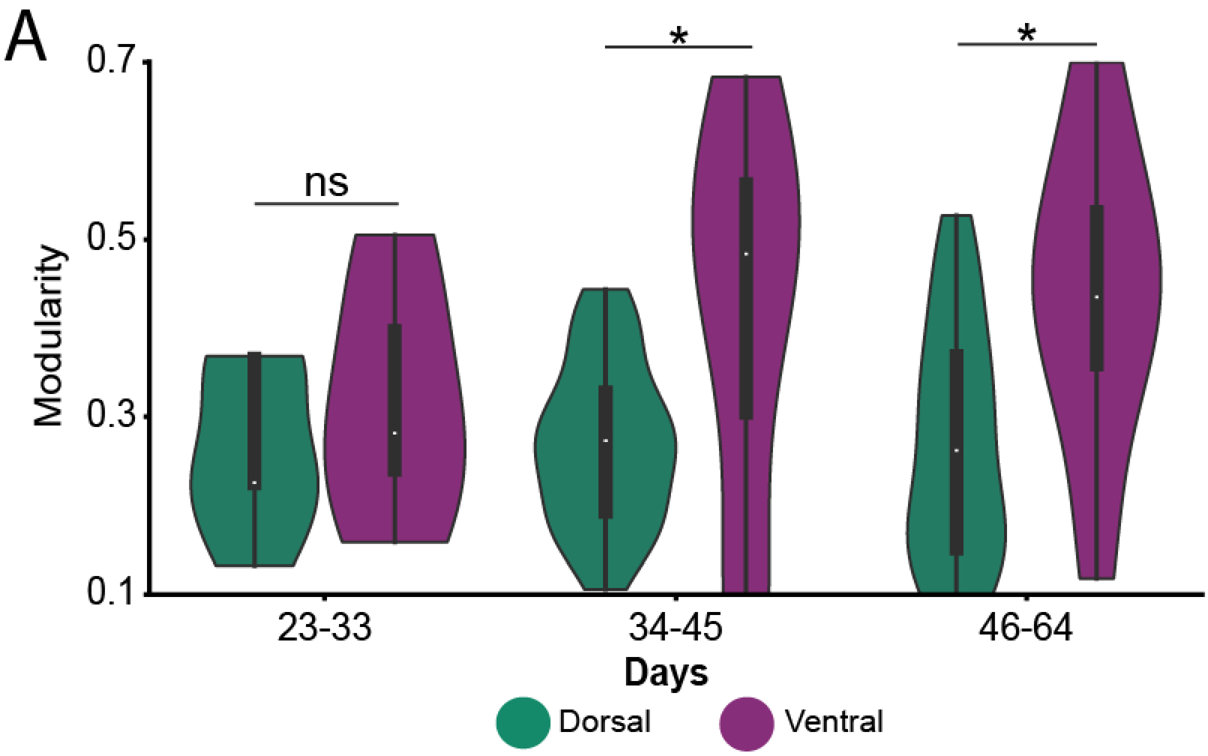

Figure S14: Developmental differences in modularity metric between DF and VF organoids, related to Figure 6.
(A) Violin plots showing network modularity values for DF (green; n = 16) and VF (purple; n = 18) organoids across three developmental time windows (23-33 days, 34-45 days, and 46-64 days). No significant difference in modularity is observed during early develop-ment (23-33 days, ns). However, VF organoids display significantly higher modu-larity compared to DF organoids during both mid (34-45 days) and late (46-64 days) developmental stages (* indicates p<0.05 Mann–Whitney U test).

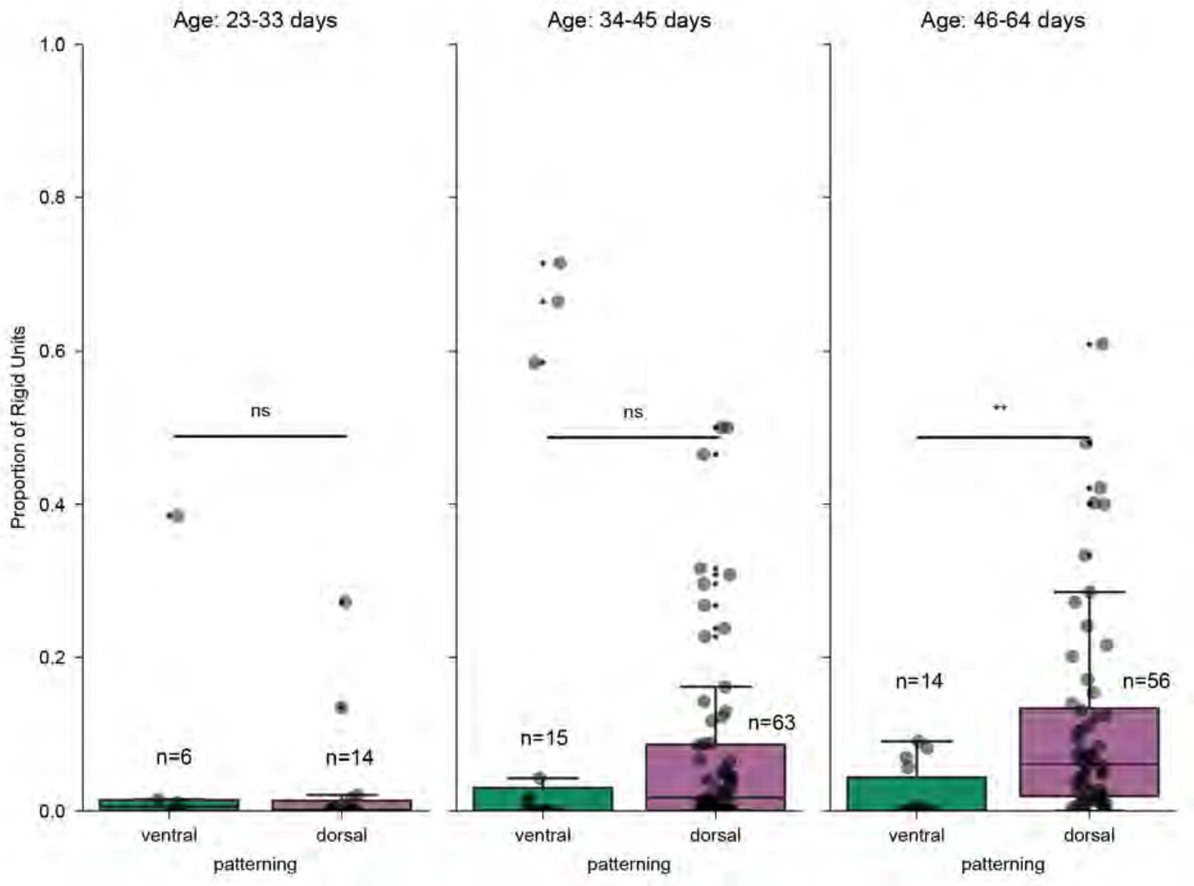

Figure S15: Proportion of rigid units over development for DF and VF, related to Figure 7.
(A) Proportion of rigid units over development for VF (Left; 23-33; n = 5; 34-45; n = 11; 46-64; n = 9)) and DF (Right; 23-33; n = 11; 34-45; n = 11; 46-64; n = 12).
ns = not significant, **p < 0.001, Mann-Whitney U test.

Supplemental Tables

Table S1: Statistical Comparison of Metrics Across Age Groups within Dorsal Organoids, related to Figure 2.
Firing rate (FR), Spike time tiling coefficient (STTC)
Mixed-effects model *p < 0.017, **p < 0.003, ***p < 0.001 (Bonferroni corrected)

Metric	Pairwise Age Group Comparisons				
	Age Groups	Diff	Std.Err.	z-value	p-value
$\log_{10}$ FR	23-33 vs 34-45	-0.202	0.053	-3.825	< 0.001**
	23-33 vs 46-64	-0.271	0.054	-5.063	< 0.001**
	34-45 vs 46-64	-0.069	0.035	-1.954	0.05
$\log_{10}$ STTC	23-33 vs 34-45	-0.087	0.040	-2.202	0.03*
	23-33 vs 46-64	-0.190	0.040	-4.728	< 0.001**
	34-45 vs 46-64	-0.103	0.027	-3.841	< 0.001**

Table S2: Statistical Comparison of Metrics Across Cell Lines within Dorsal Organoids, related to Figure 2.
Firing rate (FR), Spike time tiling coefficient (STTC)
Mixed-effects model *p < 0.017, **p < 0.003, ***p < 0.001 (Bonferroni corrected)

Metric	Age Group	Pairwise Cell Line Comparisons				
		Cell Lines	Diff	Std.Err.	z-value	p-value
$\log_{10}$ FR	23-33	C57BL6 vs E14	0.061	0.184	0.331	0.74
		C57BL6 vs KH2	-0.119	0.165	-0.722	0.47
		E14 vs KH2	-0.180	0.129	-1.399	0.16
	34-45	C57BL6 vs E14	0.144	0.055	2.614	0.009**
		C57BL6 vs KH2	-0.049	0.050	-0.973	0.33
		E14 vs KH2	-0.193	0.056	-3.464	0.001**
	46-64	C57BL6 vs E14	0.123	0.055	2.249	0.03*
		C57BL6 vs KH2	0.007	0.063	0.109	0.91
		E14 vs KH2	-0.116	0.061	-1.907	0.06
$\log_{10}$ STTC	23-33	C57BL6 vs E14	0.015	0.034	0.433	0.67
		C57BL6 vs KH2	0.046	0.031	1.509	0.13
		E14 vs KH2	0.031	0.024	1.315	0.19
	34-45	C57BL6 vs E14	0.019	0.040	0.467	0.64
		C57BL6 vs KH2	0.115	0.036	3.154	0.002**
		E14 vs KH2	0.096	0.041	2.370	0.02*
	46-64	C57BL6 vs E14	-0.040	0.051	-0.783	0.43
		C57BL6 vs KH2	0.106	0.058	1.819	0.07
		E14 vs KH2	0.146	0.057	2.586	0.01**

Table S3: Drug Effects on Firing Rate (FR), related to Figure 2.
Mixed-effects model, * Significant at p < 0.05. SEM = Standard Error of Mean

Drug	Baseline Mean (SEM)	Drug Mean (SEM)	p-value	Significant	Coefficient (SE)
APV	21.94 ± 1.04	18.78 ± 1.02	0.01	Yes*	-0.080 ± 0.108
DMSO	22.03 ± 1.19	19.63 ± 1.23	0.21	No	-0.064 ± 0.052
GABAZINE	20.81 ± 1.24	23.06 ± 1.46	0.79	No	$+0.017 \pm 0.063$
NBQX	16.64 ± 1.19	13.37 ± 1.12	0.05	No	-0.171 ± 0.088

* Significant at p < 0.05

Table S4: Drug Effects on Spike Time Tiling Coefficient (STTC), related to Figure 2.
Mixed-effects model, * Significant at p < 0.05. SEM = Standard Error of Mean

Drug	Baseline Mean (SEM)	Drug Mean (SEM)	p-value	Significant	Coefficient (SE)
APV	0.126 ± 0.010	0.132 ± 0.011	0.46	No	-0.080 ± 0.108
DMSO	0.168 ± 0.014	0.116 ± 0.010	0.52	No	-0.066 ± 0.104
GABAZINE	0.107 ± 0.011	0.188 ± 0.014	< 0.001	Yes*	$+0.352 \pm 0.084$
NBQX	0.063 ± 0.010	0.022 ± 0.004	0.003	Yes*	-0.324 ± 0.110

Table S5: Organoid size (diameter) comparisons across cell lines and patterning conditions over time, related to Figure 3.
Kruskal-Wallis tests with Dunn's post-hoc comparisons. *p < 0.05, **p < 0.01, ***p < 0.001, ns = not significant.

Comparison	Measure	Timepoints			
		Day 1	Day 5	Day 14	Day 30
Dorsal: Cell Line Comparisons					
**Overall KW Test**	p-value	**0.0088****	**0.0090****	**0.0076****	**0.0029****
	BRUCE4 vs E14	**0.0360***	**0.0131***	**0.0235***	0.0607 (ns)
	BRUCE4 vs KH2	**0.0155***	0.0573 (ns)	**0.0158***	**0.0027****
	E14 vs KH2	1.0000 (ns)	1.0000 (ns)	1.0000 (ns)	1.0000 (ns)
Ventral: Cell Line Comparisons					
**Overall KW Test**	p-value	**0.0087****	**0.0110***	**0.0105***	**0.0257***
	BRUCE4 vs E14	**0.0140***	**0.0305***	**0.0194***	0.4318 (ns)
	BRUCE4 vs KH2	**0.0400***	**0.0293***	**0.0340***	0.7148 (ns)
	E14 vs KH2	1.0000 (ns)	1.0000 (ns)	1.0000 (ns)	**0.0205***
Dorsal vs Ventral (pooled across cell lines)					
**t-test**	p-value	0.6737 (ns)	0.8490 (ns)	0.5194 (ns)	0.2925 (ns)
	n (D/V)	15/15	14/14	16/16	17/16

Table S6: Statistical Comparison of FR and STTC Across Age Groups within VF Organoids, related to Figure 4.
Firing rate (FR), Spike time tiling coefficient (STTC)
Mixed-effects model *p < 0.017, **p < 0.003, ***p < 0.001 (Bonferroni corrected)

Metric	Pairwise Age Group Comparisons				
	Age Groups	Diff	Std.Err.	z-value	p-value
$\log_{10}$ FR	23-33 vs 34-45	-0.292	0.090	-3.259	0.001**
	23-33 vs 46-64	-0.271	0.088	-3.089	0.002**
	34-45 vs 46-64	0.021	0.067	0.309	0.76
$\log_{10}$ STTC	23-33 vs 34-45	-0.100	0.048	-2.106	0.04*
	23-33 vs 46-64	-0.056	0.047	-1.193	0.23
	34-45 vs 46-64	0.045	0.036	1.251	0.21

Table S7: Statistical Comparison of FR and STTC Between DF and VF Organoids Across Age Groups, related to Figure 4.
Firing rate (FR), Spike time tiling coefficient (STTC)
Mixed-effects model, * Significant at p < 0.05.

Age Group	Metric	Coefficient		Significance		
		Estimate	Std.Err.	z-value	p-value	Significant
23-33 days	FR	-0.077	0.093	-0.828	0.41	No
	STTC	0.032	0.022	1.460	0.14	No
34-45 days	FR	0.011	0.051	0.222	0.82	No
	STTC	0.046	0.036	1.261	0.21	No
46-64 days	FR	-0.078	0.053	-1.459	0.15	No
	STTC	-0.102	0.043	-2.344	0.02*	Yes

Table S8: Age Group Comparisons for Small World Metrics in DF organoids, related to Figure 4.
Mixed-effects model *p < 0.017, **p < 0.003, ***p < 0.001 (Bonferroni corrected)

**Metric**	**Measure**	**Age Group Comparisons**		
		23-33 vs 34-45 days	34-45 vs 46-64 days	23-33 vs 46-64 days
**Small World (S)**	p-value	**< 0.001*** **	**< 0.001*** **	**< 0.001*** **
	Effect Size	0.178	0.0208	0.199
**Clustering (C_norm)**	p-value	**< 0.001*** **	**< 0.001*** **	**< 0.001*** **
	Effect Size	0.319	0.0701	0.389
**Path Length (L_norm)**	p-value	**< 0.001*** **	**< 0.001*** **	**< 0.001*** **
	Effect Size	0.046	0.0101	0.056

Table S9: Age Group Comparisons for Small World Metrics in VF organoids, related to Figure 4.
Mixed-effects model, *p < 0.017, **p < 0.003, ***p < 0.001 (Bonferroni corrected)

**Metric**	**Measure**	**Age Group Comparisons**		
		23-33 vs 34-45 days	34-45 vs 46-64 days	23-33 vs 46-64 days
**Small World (S)**	p-value	**0.003** **	**< 0.001*** **	**< 0.001*** **
	Effect Size	0.162	0.0727	0.235
**Clustering (C_norm)**	p-value	**< 0.001*** **	**< 0.001*** **	**< 0.001*** **
	Effect Size	0.202	0.3615	0.563
**Path Length (L_norm)**	p-value	**< 0.001*** **	**< 0.001*** **	**< 0.001*** **
	Effect Size	0.042	0.0377	0.080

Table S10: Patterning Comparisons (DF vs VF) by Age Group for Small World metrics, related to Figure 4.
Mixed-effects model *p < 0.05, **p < 0.01, ***p < 0.001.

Metric	Measure	Age Groups		
		23-33 days	34-45 days	46-64 days
	p-value	**< 0.001*** **	**< 0.001*** **	**< 0.001*** **
**Small World (S)**	Dorsal Mean	2.455	2.633	2.654
	Ventral Mean	3.137	3.300	3.372
	p-value	**< 0.001*** **	**< 0.001*** **	**< 0.001*** **
**Clustering (C_norm)**	Dorsal Mean	2.708	3.027	3.097
	Ventral Mean	3.723	3.925	4.286
	p-value	**< 0.001*** **	**< 0.001*** **	**< 0.001*** **
**Path Length (L_norm)**	Dorsal Mean	1.103	1.149	1.159
	Ventral Mean	1.206	1.248	1.285

Table S11: Statistical Comparison of modularity Across DF and VF age ranges, related to Figure 6.
Mixed-effects model *p < 0.017, **p < 0.003, ***p < 0.001 (Bonferroni corrected).

Comparison	p-value	Significant	Median	
			Group 1	Group 2
Dorsal vs Ventral (23-33 days)	0.30	No	0.2257	0.2814
Dorsal vs Ventral (34-45 days)	0.007	Yes**	0.2730	0.4836
Dorsal vs Ventral (46-64 days)	0.002	Yes**	0.2620	0.4349
Dorsal: 23-33 days vs 34-45 days	0.66	No	0.2257	0.2730
Dorsal: 34-45 days vs 46-64 days	0.74	No	0.2730	0.2620
Dorsal: 23-33 days vs 46-64 days	0.94	No	0.2257	0.2620
Ventral: 23-33 days vs 34-45 days	0.30	No	0.2814	0.4836
Ventral: 34-45 days vs 46-64 days	0.94	No	0.4836	0.4349
Ventral: 23-33 days vs 46-64 days	0.21	No	0.2814	0.4349

Table S12: Comparison of Rigid Unit Proportion Between DF and VF Organoids by Age Group, related to Figure 7
Mann-Whitney U test, * Significant at p < 0.05

Age Group (days)	DF Mean (± SD)	VF Mean (± SD)	U-statistic	p-value	Significant
23-33	0.033 ± 0.078	0.068 ± 0.155	39.5	0.86	No
34-45	0.076 ± 0.124	0.136 ± 0.270	582.5	0.16	No
46-64	0.112 ± 0.137	0.022 ± 0.035	640.0	< 0.001	Yes*
Overall	0.087 ± 0.131	0.079 ± 0.185	3164.0	0.001	Yes*

Table S13: Comparison of Bursting Dynamics Between DF and VF Organoids, related to Figure 7.
Kolmogorov-Smirnov test, * Significant at p < 0.05. SEM = Standard Error of Mean

Measure	DF Mean (SEM)	VF Mean (SEM)	p-value	Sig	Effect Size
Burst Correlation	0.239 ± 0.017	0.191 ± 0.014	0.001	Yes*	0.249
Timing Variability (ms)	95.2 ± 0.9	94.0 ± 1.4	0.02	Yes*	0.021

SUPPLEMENTAL METHODS

mESC Maintenance

mESCs were maintained on plates coated with 0.5 µg/mL recombinant human vitronectin (Thermo Fisher Scientific #A14700) in 1X PBS (pH~7.4; Thermo Fisher Scientific #70011044) for 15 min at room temperature. Cells were cultured in mESC maintenance medium consisting of Glasgow Minimum Essential Medium (GMEM; Thermo Fisher Scientific #11710035) supplemented with 10% embryonic stem cell-qualified fetal bovine serum (Thermo Fisher Scientific #10439001), 0.1 mM MEM Non-Essential Amino Acids (Thermo Fisher Scientific #11140050), 1 mM sodium pyruvate (Millipore Sigma #S8636), 2 mM GlutaMAX supplement (Thermo Fisher Scientific #35050061), 0.1 mM 2-mercaptoethanol (Millipore Sigma #M3148), 0.05 mg/mL Primocin (InvivoGen #ant-pm-05), and 1000 U/mL recombinant mouse leukemia inhibitory factor (Millipore Sigma #ESG1107), with daily medium changes.

Cells were passaged using ReLeSR (Stem Cell Technologies #05872) according to manufacturer instructions and cryopreserved in mFreSR medium (Stem Cell Technologies #05855). All lines were tested for mycoplasma, and tested negative, every 6-9 months (MycoAlert #Lonza LT07-318).

GMEM-Based DF Organoids Generation

mESCs were dissociated into single cells with TrypLE Express (Thermo Fisher Scientific #12604021) for 5 minutes at 37 °C and re-aggregated in Lipidure-coated 96-well V-bottom plates at 3,000 cells per well in 100 µL of differentiation medium. The medium consisted of Glasgow Minimum Essential Medium (GMEM Thermo Fisher Scientific #11710035) supplemented with 10% KnockOut Serum Replacement (Thermo Fisher Scientific #10828028), 0.1 mM MEM Non-Essential Amino Acids (Thermo Fisher Scientific #11140050), 1 mM Sodium Pyruvate (Millipore Sigma #S8636), 2 mM GlutaMAX (Thermo Fisher Scientific #35050061), 0.1 mM 2-Mercaptoethanol (Millipore Sigma #M3148), and 0.05 mg/mL Primocin (InvivoGen #ant-pm-05). Additional supplements included 20 µM Y27632 (Tocris Bioscience #1254), 3 µM IWR1-ε (Cayman Chemical #13659), and 5 µM SB431542 (Tocris Bioscience #1614). Medium was changed daily from days 0 to 7.

On day 7, organoids were transferred to ultra-low adhesion plates (Millipore Sigma #CLS3471) with N2 medium composed of DMEM/F12 with GlutaMAX (Thermo Fisher Scientific #10565018), 1X N2 Supplement (Thermo Fisher Scientific #17502048), and Primocin. Cultures were maintained on an orbital shaker at 60 rpm under 5% CO_2 with medium changes every 2-3 days.

From day 14 onward, organoids were cultured in neuronal maturation medium containing BrainPhys Neuronal Medium (Stem Cell Technologies #05790), supplemented with N2, Chemically Defined Lipid Concentrate (Thermo Fisher Scientific #11905031), B27 Supplement (Thermo Fisher Scientific #17504044), Primocin, and 0.5% (v/v) Matrigel GFR (Corning #354230).

Single-Cell Dissociation

Organoids (8-10 per genotype) were dissociated using the Worthington Papain Dissociation System (#LK003150). The dissociation solution consisted of 20 U/mL papain, 1 mM L-cysteine, and 0.5 mM EDTA in EBSS, activated for 30 min at 37 °C. DNase I (200 U/mL) was added post-activation. Tissue samples were incubated in this solution for 30 min at 37 °C with gentle agitation every ~10 min, then mechanically dissociated using flame-polished glass Pasteur pipettes (Fisher Scientific #13-678-6B). After centrifugation (300 RCF, 3 min), cells were resuspended in 1X PBS with 0.1% BSA (Millipore Sigma #A3311), filtered through a 40 µm strainer (Corning #431750), and manually counted.

scRNAseq and Computational Analysis

Sequencing was conducted on an AVITI PE75 Flowcell (Element Biosciences) at the UC Davis Technologies Core, generating approximately 900 million reads. Raw FASTQ files were processed using the PIPseeker pipeline (v3.3), with default parameters for alignment, transcript quantification, and cell calling against the GRCm39 mouse reference genome (GENCODE vM29 2022.04, Ensembl 106).

Downstream analysis was performed in Seurat (v5.1.0) (Hao et al., 2024) using sensitivity 5 matrices. Quality control steps included:

- Genotype demultiplexing with Souporcell (Heaton et al., 2020).
- Doublet removal using DoubletFinder v2.0.4 (McGinnis et al., 2019).
- Batch integration using Harmony (Korsunsky et al., 2019).

Cells were excluded if they had mitochondrial content > 20%, unique gene counts below the 5th percentile, or total RNA counts > 50,000. SCTransform was applied with mitochondrial regression,variable genes were selected (Lause et al., 2021; Choudhary and Satija, 2022). Dimensionality and 3,000 variable genes was performed using 40 principal components, followed by Leiden clustering at resolutions from 0.5 to 2. Clusters were visualized using UMAP (Becht et al., 2019), and resolution selection was guided by marker gene expression. Cell types were annotated using the Allen Brain Atlas (Yao et al., 2021}), UCSC Cell Browser (Speir et al., 2021), and the Arlotta developmental atlas (Di Bella et al., 2021). For reference mapping, dorsal forebrain datasets were integrated using Seurat's standard workflow: log-normalization (scale factor 10,000), variable gene selection, data scaling, and PCA (30 components). Harmony was used for integration prior to label transfer via canonical correlation analysis (CCA)-based anchor identification (Hao et al., 2024). Neuronal cells were subset and dimensionality reduction was redone after reperforming SCTransform to rescale neuronal genes.

Viability testing

Organoids were transferred to 1.5 mL microcentrifuge tubes containing 500 µL of culture medium. Acridine orange-propidium iodide (AOPI) dye (Revvity #CS2-0106-5ML) was added at a 1:1,000 dilution and incubated for 30 minutes at room temperature. Following incubation, organoids were washed with 1× PBS and imaged using an EVOS M7000 microscope.

Organoid size measurement

Organoids were imaged on a standard tissue culture microscope at 4× magnification using an iPhone 15 camera at 1× zoom. A calibration slide was used to establish a pixel-to-micrometer conversion. Organoid diameters were measured using the straight-line and Measure tools in Fiji (v1.54p). Measurements were exported as CSV files and analyzed using Python (v3.11).

qPCR

DF and VF organoids were collected at Day 30, Day 52, and Day 60 across three independent batches. For each condition and batch, three organoids derived from the same plate were pooled to form a single biological replicate (N=3 biological replicates per condition).

Total RNA was isolated from pooled organoid samples using TRIzol® reagent (Thermo Fisher Scientific #15596026) according to the manufacturer's instructions, involving chloroform extraction and isopropanol precipitation. The resulting RNA pellet was washed with 75% ethanol and resuspended. RNA concentration and purity were verified using a NanoDrop spectrophotometer. cDNA was synthesized from 1000 ng of total RNA using the SuperScript IV Reverse Transcriptase system (Thermo Fisher Scientific #18090010), employing oligo(dT) primers (Thermo Fisher Scientific #AM5730G). Key components included DTT (Thermo Fisher Scientific #707265ML) and RNaseOUT Recombinant RNase Inhibitor (Thermo Fisher Scientific #10777019). The cDNA was stored at −20 °C.

qPCR reactions were performed using TaqMan Gene Expression Assays (FAM; Thermo Fisher Scientific #4453320) on a QuantStudio Pro Real-Time PCR System (Thermo Fisher Scientific). We targeted Gapdh (housekeeping control), Camk2a (excitatory neuron marker), Pvalb (PV+ interneuron marker), and Gad2 (pan-GABAergic marker). Reactions were assembled using TaqMan Fast Advanced Master Mix (Thermo Fisher Scientific #4444556), cDNA template, and the corresponding TaqMan probe. Each batch included all six experimental conditions run with three technical replicates per gene. The standard cycling program was used: an initial hold (50°C for 2 min and 95°C for 10 min), followed by 40 cycles of denaturation (95°C for 15 s) and annealing/extension (60°C for 1 min). Fluorescence data was collected during each extension step.

Relative gene expression was determined by calculating ΔCt for each sample (Ct_target − Ct_Gapdh). These ΔCt values were converted to expression ratios using $2^{-\Delta Ct}$, providing a normalized measure proportional to transcript abundance. For both dorsal and ventral samples, expression ratios (Pvalb/Camk2a and Gad2/Camk2a) were calculated by dividing the corresponding normalized $2^{-\Delta Ct}$ values for each gene within the same sample. Statistical analyses were performed directly on these gene expression ratios.

Cryosection Immunohistochemistry

Organoids were fixed in 4% paraformaldehyde (Thermo Fisher Scientific #28908), cryoprotected in 30% sucrose (Millipore Sigma #S8501), and embedded in a 1:1 mix of Tissue-Tek O.C.T. Compound (Sakura #4583) and 30% sucrose. Cryosections (20 µm) were cut

using a Leica CM3050 and collected directly onto slides. After PBS washes, sections were blocked (5% donkey serum, 0.1% Triton X-100) for 1 h, incubated with primary antibodies overnight at 4 °C, washed, then incubated with secondary antibodies for 90 min at room temperature. After final washes, sections were mounted with Fluoromount-G (Thermo Fisher Scientific #00-4958-02).

Vibratome Section Immunohistochemistry

For whole-mount analysis, organoids were fixed in 4% PFA (4 °C, overnight), embedded in 4% low-melt agarose (Invitrogen #16520-050), and sectioned at 50 µm using a Leica VT1000s vibratome. Sections underwent sequential blocking:

Initial block: 5% donkey serum, 1% BSA, 0.5% Triton X-100 (4 °C, 1 h)
Antibody block: 2% donkey serum, 0.1% Triton X-100 with primary antibodies (overnight, 4 °C)

Following PBS washes, sections were incubated with secondary antibodies (30 min, RT), counterstained with Hoechst 33342 or DAPI, and mounted with Fluoromount-G (Fisher Scientific #OB100-01).

Antibody Panel and Imaging

The following primary antibodies were used for immunohistochemistry, listed alphabetically by target antigen:

- Anti-Brn2 (rabbit; Thermo Fisher Scientific #PA530124, RRID:AB_2547598; 1:400)
- Anti-Cox6A2 (rabbit; Novus Biologicals #NBP1-31112; RRID:AB_2085447; 1:100)
- Anti-Ctip2 (rat; Abcam #ab18465, RRID:AB_2064130; 1:250)
- Anti-GABA (rabbit; Thermo Fisher Scientific #PA5-32241, RRID:AB_2549714; 1:375)
- Anti-Gfap (mouse; Thermo Fisher Scientific #G6171, RRID:AB_1840893; 1:100)
- Anti-Map2 (chicken; Thermo Fisher Scientific #PA1-10005 ; RRID: AB_1076848; 1:1000)
- Anti-Map2 (rabbit; Proteintech #17490-1-AP, RRID:AB_2137880; 1:2000)
- Anti-N-cadherin (mouse; Abcam #ab98952, RRID:AB_10696943; 1:250)
- Anti-Nkx2.1 (rabbit; Abcam #ab76013, RRID:AB_1310784; 1:400)
- Anti-Parvalbumin (rabbit; Swant #PV27, RRID:AB_2631173; 1:375)
- Anti-Pax6 (mouse; BD Biosciences #561462, RRID:AB_10715442; 1:100)
- Anti-Pkcζ (mouse; Santa Cruz Biotechnology #sc17781, RRID:AB_628148; 1:500)
- Anti-Satb2 (mouse; Abcam #ab51502, RRID:AB_882455; 1:100)
- Anti-Sox2 (mouse; Santa Cruz Biotechnology #sc365823, RRID:AB_10842165; 1:500)
- Anti-Sst (mouse; Santa Cruz Biotechnology #sc55565, RRID:AB_831726; 1:100)

Secondary detection used Alexa Fluor-conjugated antibodies (1:750). Nuclear counterstaining was performed with 300 nM DAPI (Thermo Fisher Scientific #D1306).

For perineuronal nets detection we used and biotinylated WFA (Vector Laboratories #B-1355-2, RRID:AB_2336874; 1:200) followed by Alexa 488-streptavidin (Thermo Fisher Scientific #S11223; 1:500).

Analysis of Immunohistochemistry

Organoid imaging was performed on a Zeiss AxioImager Z2 microscope with 10x magnification using Zen Blue software. Z-stacks were acquired at 1.53 µm spacing from three non-adjacent cryosections per organoid, with tile scanning for larger samples. The analysis included 4-5 organoid replicates per cell line and condition (DF or VF) across two independent mESC lines (ES-E14TG2a and KH2).

Raw .czi files were converted to .ims format using the Imaris file converter, deconvolved in AutoQuant X3 3.1, and analyzed in Imaris (v10.2). Nuclear segmentation was performed on the DAPI channel. Spot detection used an XY diameter of 4.5 µm, PSF model elongation of 15 µm, background subtraction, quality filter threshold >1747, and nearest-neighbor distance between 4.83 and 12.0 µm.

For marker quantification, Pax6+ and Nkx2.1+ cells were identified using the same spot detection parameters, with additional colocalization filters requiring a maximum distance of 14 µm from DAPI+ nuclei. The pipeline was automated using Imaris Arena with consistent parameters across all patterning conditions.

Exported metrics included DAPI+ cell counts and percentages of Pax6+/DAPI+ and Nkx2.1+/DAPI+ double-positive cells. Statistical analysis was performed on 162 dorsal and 113 ventral images using a Mann-Whitney U test to compare proportions between conditions. Significance was defined as $p < 0.05$. Quality control measures included blinded analysis (experimenter masked to conditions).

For 3D image reconstruction, .czi files were converted and deconvolved as described above. Images and videos were exported using Imaris software (Oxford Instruments) and saved as PNG or MP4 files, respectively.

Electrophysiology Preparation

MEAs were coated with 0.01% polyethylenimine (Millipore Sigma #408727) in 1X PBS for 1 h at 37 °C, then washed three times with deionized water and air-dried for 10 min. They were subsequently coated with 20 µg/mL mouse laminin (Fisher Scientific #CB40232) and 5 µg/mL human fibronectin (Fisher Scientific #CB40008) in 1X PBS for 1 h at 37 °C. Organoids were placed onto the coated MEAs, excess medium was removed, and samples were incubated at 37 °C for 5-8 min to promote adhesion before adding pre-warmed neuronal differentiation medium.

Electrophysiological Data Processing

Electrophysiological activity was monitored every 2-3 days using Maxwell Biosystems acquisition software, sampling signals from 1024 of the ~26,000 electrodes in a sweeping checkerboard pattern (30 s per configuration). The 1,020 most active electrodes, spaced at least 50 µm apart, were selected for recording to ensure single-unit resolution. All recordings were performed in a humidified incubator (5% CO_2, 37 °C) at a sampling rate of 20 kHz and saved in HDF5 format.

Raw extracellular recordings were band-pass filtered between 300-6,000 Hz and spike-sorted using Kilosort2 (Pachitariu et al., 2016; Hill et al., 2011) within a custom Python pipeline. Units were excluded if they exhibited interspike interval (ISI) violation rates > 0.5, mean firing rates < 0.1 Hz, or signal-to-noise ratios (SNR) < 3.

Pharmacological Modulation of Neuronal Activity

DF organoids aged 60 to 65 days were scanned for spontaneous activity, and electrodes were selected based on the highest activity levels as described in the Electrophysiological Data Processing section.

Drug concentrations were selected based on established effective doses from previous studies (Mayer et al., 2019; van der Molen et al., 2025). Following a 10-minute baseline recording, the following pharmacological agents were applied:

- Gabazine (SR95531; Abcam #ab120042) at 1 μM.
- NBQX (Abcam #ab120045) at 20 μM.
- APV at 100 μM.

Stock solutions were prepared for 1:1000 dilution in culture medium. Gabazine and NBQX were dissolved in DMSO, and APV in water. After drug administration, organoids were incubated for 30 minutes before acquiring a 10-minute recording of drug-modulated activity.

Recordings were processed using the following pipeline:

- Concatenation using SpikeInterface (Hill et al., 2011).
- Spike sorting as described in the Electrophysiological Data Processing section.
- Manual curation using Phy visualization software (Rossant et al., 2013).

STTC Analysis

We quantified pairwise neuronal synchronization using the STTC with a $\Delta t = 10$ ms timescale (Chini et al., 2022; Chini et al., 2024; Cutts and Eglen, 2014) . The STTC is defined as:

$$\mathrm{STTC} = \frac{1}{2}\left(\frac{P_A - T_B}{1 - P_A T_B} + \frac{P_B - T_A}{1 - P_B T_A}\right)$$

where:

- P_A is the proportion of spikes in train A that fall within $\pm\Delta t$ of any spike in train B.
- T_A is the proportion of the total recording duration that lies within $\pm\Delta t$ of spikes in train A.
- P_B and T_B are the corresponding quantities for spike train B.

This symmetric measure ranges from -1 (perfect anti-correlation) to +1 (perfect synchrony), with 0 indicating independence.

Functional Network Analysis

Network Construction

Functional connectivity matrices were derived from thresholded, binarized STTC values. To establish significance thresholds while preserving population rate dynamics, we:

1. Generated 1,000 surrogate datasets by spike identity shuffling.
2. Computed STTC distributions from shuffled data.
3. Set thresholds at the 90th percentile of null distributions.
4. Binarized matrices using these subject-specific thresholds.

Global Network Metrics

Using NetworkX (Hagberg et al., 2008) and custom Numba-accelerated functions, we computed:

- **Clustering coefficient**: Local density of connections using a Numba-accelerated parallel implementation (compute_clustering_coeff_parallel).
- **Characteristic path length**: Mean shortest path distance using NetworkX's (average_shortest_path_length) on the largest connected component.

All metrics were normalized by dividing by corresponding values from 100 synthetic random networks via generated (generate_random_graph) with identical node and edge counts.

Small-worldness was calculated as:

$$\text{Small-worldness} = \frac{C/C_{rand}}{L/L_{rand}}$$

where C and L are the clustering coefficient and path length, respectively. Binary functional networks were created using STTC values thresholded at the 90th percentile of surrogate values obtained by shuffling neuron identities across 1,000 randomized networks while preserving firing rate distributions.

Hub Identification

We computed a composite hubness score by integrating four nodal metrics:

- **Degree**: Number of connections (degrees_und).
- **Strength**: Sum of connection weights (strengths_und, using weighted matrices).
- **Betweenness centrality**: Fraction of shortest paths passing through nodes (betweenness_bin).
- **Closeness centrality**: Inverse average shortest path length (distance_bin derived).

Each metric was z-scored across nodes before summation to create the composite score.

Analysis computed:

- Firing rate distributions (mean ± SEM across replicates).
- Coefficient of variation (CV) of interspike intervals.
- Population synchrony (pairwise spike train correlations).
- E/I balance ratios (excitatory vs. inhibitory input currents).
- Weight distribution evolution (Kolmogorov-Smirnov tests).

SUPPLEMENTAL REFERENCES

Becht, E., McInnes, L., Healy, J., Dutertre, C., Kwok, I.W.H., Ng, L.G., Ginhoux, F. and Newell, E.W. (2019). Dimensionality Reduction for Visualizing Single-Cell Data Using UMAP. Nat. Biotechnol. *37*, 38-44.

Choudhary, S. and Satija, R. (2022). Comparison and Evaluation of Statistical Error Models for scRNA-seq. Genome Biol. *23*, 27.

Cutts, C.S. and Eglen, S.J. (2014). Detecting Pairwise Correlations in Spike Trains: An Objective Comparison of Methods and Application to the Study of Retinal Waves. J. Neurosci. *34*, 14288-14303.

Hagberg, A.A., Schult, D.A. and Swart, P.J. (2008). Exploring network structure, dynamics, and function using NetworkX. Proceedings of the 7th Python in Science Conference (SciPy2008) , 11-16.

Hao, Y., Stuart, T., Kowalski, M.H., Choudhary, S., Hoffman, P., Hartman, A., Srivastava, A., Molla, G., Madad, S., Fernandez-Granda, C., et al. (2024). Dictionary Learning for Integrative, Multimodal and Scalable Single-Cell Analysis. Nat. Biotechnol. *42*, 293-304.

Heaton, H., Talman, A.M., Knights, A., Imaz, M., Gaffney, D.J., Durbin, R., Hemberg, M. and Lawniczak, M.K.N. (2020). Souporcell: Robust Clustering of Single-Cell RNA-seq Data by Genotype Without Reference Genotypes. Nat. Methods *17*, 615-620.

Hill, D.N., Mehta, S.B. and Kleinfeld, D. (2011). Quality Metrics to Accompany Spike Sorting of Extracellular Signals. J. Neurosci. *31*, 8699-8705.

Korsunsky, I., Millard, N., Fan, J., Slowikowski, K., Zhang, F., Wei, K., Baglaenko, Y., Brenner, M., Loh, P. and Raychaudhuri, S. (2019). Fast, Sensitive and Accurate Integration of Single-Cell Data with Harmony. Nat. Methods *16*, 1289-1296.

Lause, J., Berens, P. and Kobak, D. (2021). Analytic Pearson Residuals for Normalization of Single-Cell RNA-seq UMI Data. Genome Biol. *22*, 258.

Mayer, S., Chen, J., Velmeshev, D., Mayer, A., Eze, U.C., Bhaduri, A., Cunha, C.E., Jung, D., Arjun, A., Li, E., et al. (2019). Multimodal Single-Cell Analysis Reveals Physiological Maturation in the Developing Human Neocortex. Neuron *102*, 143-158.e7.

McGinnis, C.S., Murrow, L.M. and Gartner, Z.J. (2019). DoubletFinder: Doublet Detection in Single-Cell RNA Sequencing Data Using Artificial Nearest Neighbors. Cell Syst. *8*, 329-337.e4.

Pachitariu, M., Steinmetz, N.A., Kadir, S.N., Carandini, M. and Harris, K.D. (2016). Fast and accurate spike sorting of high-channel count probes with KiloSort. 30th Conference on Neural Information Processing Systems (NIPS 2016), pp. 4455-4463. Barcelona, Spain.

Rossant, C., Hunter, M., Steinmetz, N., Wallace, M., Spacek, M., Gestes, C., McKenzie, Z., Nolan, C., Buccino, A., Zapp, S., et al. (2023). phy: Interactive Visualization and Manual Spike Sorting of Large-Scale Ephys Data [Python]. The Cortical Processing Laboratory at UCL.

Speir, M.L., Bhaduri, A., Markov, N.S., Moreno, P., Nowakowski, T.J., Papatheodorou, I., Pollen, A.A., Raney, B.J., Seninge, L., Kent, W.J., et al. (2021). UCSC Cell Browser: visualize your single-cell data. Bioinformatics *37*, 4578-4580.

Yao, Z., van Velthoven, C.T.J., Kunst, M., Zhang, M., McMillen, D., Lee, C., Jung, W., Goldy, J., Abdelhak, A., Aitken, M., et al. (2023). A High-Resolution Transcriptomic and Spatial Atlas of Cell Types in the Whole Mouse Brain. Nature *624*, 317-332.
