# Supplementary material for: Establishing mouse forebrain organoids as models of intrinsic cortical network assembly
Source: Stem Cell Reports. 2026 Mar 5;21(4):102832. doi: 10.1016/j.stemcr.2026.102832 (PMC13083800; doi:10.1016/j.stemcr.2026.102832)
Supplement: Document S2. Article plus supplemental information [file mmc4.pdf]

STEM CELL REPORTS
Article
ISSCR
OPEN ACCESS

Establishing mouse forebrain organoids as models of intrinsic cortical network assembly

Sebastian Hernandez,[1,2,11] Hunter E. Schweiger,[1,3,11] Isabel Cline,[1,4] Gregory A. Kaurala,[1] Ash Robbins,[1,2] Daniel Solis,[1,5] Samira Vera-Choqqueccota,[1,5] Jinghui Geng,[1,2] Tjitse van der Molen,[1,5,6,7] Francisco Reyes,[1,8] Chinweike Norman Asogwa,[3] Kateryna Voitiuk,[1,2] Mattia Chini,[9,10] Marco Rolandi,[1,2] Sofie R. Salama,[1,3] Bradley M. Colquitt,[1,3] Tal Sharf,[1,5] David Haussler,[1,5] Mircea Teodorescu,[1,2,5,*] and Mohammed A. Mostajo-Radji[1,12,*]

[1]Genomics Institute, University of California, Santa Cruz, Santa Cruz, CA 95064, USA
[2]Department of Electrical and Computer Engineering, University of California, Santa Cruz, Santa Cruz, CA 95064, USA
[3]Department of Molecular, Cellular and Developmental Biology, University of California, Santa Cruz, Santa Cruz, CA 95064, USA
[4]Department of Chemistry and Biochemistry, University of California, Santa Cruz, Santa Cruz, CA 95064, USA
[5]Department of Biomolecular Engineering, University of California, Santa Cruz, Santa Cruz, CA 95064, USA
[6]Neuroscience Research Institute, University of California, Santa Barbara, Santa Barbara, CA 93106, USA
[7]Department of Molecular, Cellular and Developmental Biology, University of California, Santa Barbara, Santa Barbara, CA 93106, USA
[8]Biotechnology Program, Berkeley City College, Berkeley, CA 94704, USA
[9]Institute of Developmental Neurophysiology, Center for Molecular Neurobiology, University Medical Center Hamburg-Eppendorf, Hamburg, Germany
[10]Present address: GIGA Neurosciences, University of Liège, CHU Sart Tilman, B-4000 Liège, Belgium
[11]These authors contributed equally
[12]Lead contact
*Correspondence: mteodore@ucsc.edu (M.T.), mmostajo@ucsc.edu (M.A.M.-R.)
https://doi.org/10.1016/j.stemcr.2026.102832

SUMMARY

The mouse cortex is a canonical model for studying how functional neural networks emerge, yet it remains unclear which topological features arise from intrinsic cellular organization versus sensory input. Mouse forebrain organoids provide a powerful system to investigate these intrinsic mechanisms. We generated dorsal (DF) and ventral (VF) forebrain organoids from mouse pluripotent stem cells and tracked their development using longitudinal electrophysiology. DF organoids showed progressively stronger network-wide correlations, while VF organoids developed more refined activity patterns with enhanced small-world topology and increased modular organization. Both organoid types form small-world networks, but their topological organization differs. These differences emerge without extrinsic inputs and correlate with Pvalb^{+} interneuron enrichment in VF organoids. Our findings demonstrate how cellular composition influences neural circuit self-organization, establishing mouse forebrain organoids as a tractable platform to study cortical network architecture.

INTRODUCTION

The brain is organized as a complex network whose topology supports efficient computation. Across species, a key feature is small-world architecture, defined by high local clustering and short path lengths that enable efficient information segregation and integration with minimal wiring and metabolic costs (Achard et al., 2006; Bassett and Bullmore, 2017; Watts and Strogatz, 1998). This topology is conserved across spatial scales and species, from the *C. elegans* connectome to the brains of mice, cats, macaques, and humans (Bassett and Bullmore, 2017). In humans, the small-world organization of neural circuits changes during development and aging, and its disruption has been linked to neuropsychiatric disorders (Liao et al., 2017). Despite extensive characterization of adult brain networks, the developmental processes that generate this topology remain poorly understood.

Dissociated neuronal cultures have shown that both network topology and cellular composition shape emergent dynamics through self-organization. Effective connectivity networks progressively develop small-world architecture, modular organization, and hub neurons as cultures mature (Antonello et al., 2022; Downes et al., 2012; Schroeter et al., 2015), while excitatory-inhibitory (E-I) balance strongly influences activity patterns (Crocco et al., 2025). Yet, 2D monolayers lack spatial constraints and regional organization of intact tissue, and *in vivo*, it is difficult to separate cellular composition from sensory-driven activity. This makes it challenging to accurately model how 3D cytoarchitecture and cellular diversity alone shape network topology.

Brain organoids derived from pluripotent stem cells (PSCs) provide 3D models that recapitulate key aspects of brain development, including neuronal subtype diversity, layered structure, and functional synaptic networks (Velasco et al., 2019). Most research has focused on human organoids, which generate spontaneous electrical activity that intensifies and becomes more structured with maturation (Alam El Din et al., 2025; Mancinelli et al., 2025;

Published by Elsevier Inc. on behalf of International Society for Stem Cell Research.
This is an open access article under the CC BY license (http://creativecommons.org/licenses/by/4.0/).

Samarasinghe et al., 2021; Sharf et al., 2022; Trujillo et al., 2019; van der Molen et al., 2026). Longitudinal multielectrode array (MEA) recordings show progressive increases in firing rate, synchrony, and oscillatory complexity across months in culture, paralleling developmental transitions observed in neonatal electroencephalograms (EEGs) (Trujillo et al., 2019; Sharf et al., 2022; Mancinelli et al., 2025; Chini et al., 2022). *In vivo*, developing cortical networks initially exhibit highly synchronized bursts of activity that gradually become more independent as inhibitory circuits mature. This transition, first characterized in mice and later observed in humans, reflects conserved mechanisms through which increasing inhibitory tone refines E-I balance (Wu et al., 2024).

The developing mouse cortex provides well-characterized benchmarks for circuit assembly. Early synchronized activity becomes sparser and less correlated as inhibitory tone increases, while networks acquire hub neurons and small-world topology with dense local clustering and sparse long-range connections (Chini et al., 2022, 2024; Golshani et al., 2009; Hilgetag and Kaiser, 2004; Wu et al., 2024). These features arise before sensory experience, offering valuable reference points for organoid models. Mouse organoids are therefore ideally suited to test how regional cellular composition shapes emergent network topology.

Efforts to model mouse forebrain development with organoids date back over two decades but remain technically limited. The Sasai group first described PSC-derived mouse forebrain organoids in 2005, later refining the protocol using Glasgow Minimum Essential Medium (GMEM)-based conditions (Watanabe et al., 2005; Eiraku et al., 2008). Subsequent studies followed this approach (Elliott et al., 2023; Ferdaos et al., 2022; Mall et al., 2017; Park et al., 2024) or used reaggregated primary progenitors (Mostajo-Radji et al., 2025; Tanveer et al., 2025). Other efforts produced unguided organoids with partial forebrain identity (Lindhout et al., 2025; Sánchez et al., 2024) or achieved limited cortical induction (Medina-Cano et al., 2022). More recently, N2B27-based protocols have generated organoids with cortical projection neurons (PNs) that persist for up to 40 days (Li et al., 2020; Medina-Cano et al., 2025). Nonetheless, protocols that yield mouse forebrain organoids with sustained network activity and functional synaptic connectivity are still lacking.

Here, we address a key gap in the field by systematically investigating how regional cellular composition shapes the formation of neural networks independent of sensory input. We established a protocol that generates dorsal (DF) and ventral (VF) forebrain organoids with robust, long-lasting neural activity from mouse PSCs and characterized their network development. Despite differences in initial patterning, both organoids generate neocortical cell types but with markedly different inhibitory neuron content, providing complementary systems to investigate how E-I balance influences cortical circuit assembly. DF organoids, composed primarily of excitatory PNs, exhibit progressive synchronization, whereas VF organoids, enriched in GABAergic interneurons (INs; including Pvalb$^+$ INs), show refined temporal coordination, enhanced small-world topology, and increased modular organization with stable connectivity patterns. Although both types form small-world networks, they differ in topological organization, demonstrating how cellular composition influences circuit formation. These findings establish mouse forebrain organoids as a tractable platform to study how neuronal populations shape cortical network architecture and to probe the developmental logic underlying circuit assembly and its potential disruption in disease. Importantly, this platform allows mechanistic testing not feasible in human organoids, including causal perturbations and *in vivo* validation using genetically identical controls.

RESULTS

A standardized protocol for DF organoid generation

GMEM-based DF organoids generate active neurons, but these are sparse and unsuitable for modeling circuit-level dynamics (Eiraku et al., 2008; Park et al., 2024). To address this, we established a robust protocol for generating DF organoids by aggregating 3,000 mouse embryonic stem cells (mESCs) per well, inducing DF identity via WNT and TGFβ inhibition, and promoting neuronal differentiation through a stepwise Neurobasal-A/BrainPhys to BrainPhys media transition (Figure 1A; see “methods”). This protocol increased radial glia abundance relative to GMEM-based methods, indicating improved DF specification, while reducing off-target lineages and enhancing neuronal yield (Figure S1).

We tested the protocol in 3 genetically distinct mESC lines: BRUCE4 (C57BL/6 background), ES-E14TG2a (129/Ola background), and KH2 (C57BL/6 × 129/Sv hybrid background). DF organoids yielded consistent sizes over time, with the BRUCE4 line being slightly smaller than the other 2 lines (Figure 1B). Immunohistochemistry (IHC) revealed stage-specific marker expression in DF organoids. By day 10, Sox2$^+$ progenitors, Pkcζ$^+$ polarity, and N-cadherin$^+$ neuroepithelium were evident (Figures 1C, S2A, and S2B). Organoids expressed the intermediate progenitor marker Tbr2, the neuronal marker Tubb3, and DF markers Tbr1 and Brn2 (Figures 1C and S2B–S2D), reflecting mid corticogenesis with deep-layer Tbr1$^+$ PNs and upper-layer Brn2$^+$ progenitors and PNs (Di Bella et al., 2021). Small GABA$^+$ IN populations were also detected (Figure S2E). By days 30–40, organoids expressed Ctip2

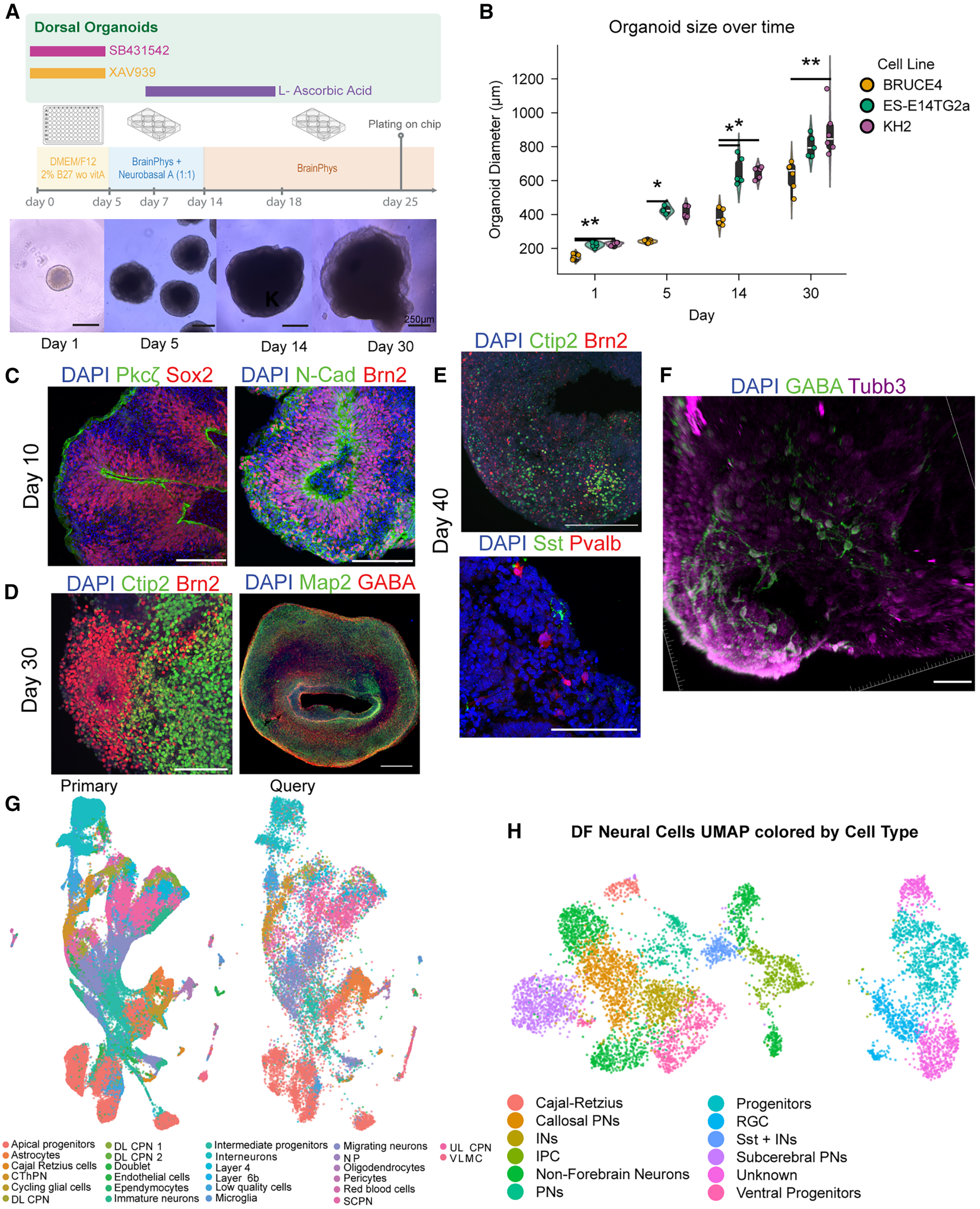

Figure 1. An optimized protocol for DF organoid development

(A) Schematic of the protocol for DF organoid development.

(B) Organoid diameter across 3 cell lines at days 1, 5, 14, and 30. $*p < 0.05$, $**p < 0.003$, Tukey's HSD. $n = 5$ organoids for each cell line.

(C) IHC of DF organoids at day 10 stained for: (left) Pkcζ (green) and Sox2 (red); (right) N-cadherin (green) and Brn2 (red).

(D) Day 30 DF organoids stained for: (left) Ctip2 (green) and Brn2 (red); (right) Map2 (green) and GABA (red).

(E) Day 40 DF organoids stained for (top) Ctip2 (green) and Brn2 (red); (bottom) Sst (green) and Pvalb (red).

(F) 3D reconstruction of day 40 DF organoid stained for GABA (green) and Tubb3 (magenta). DAPI nuclear counterstain shown in blue. Scale bars, 100 µm.

(legend continued on next page)

and Brn2 in corticofugal and callosal PNs, respectively, and contained Gfap$^+$ astrocytes and GABA$^+$ INs (Figures 1D, 1E, and S2F–S2H). Consistent with prior reports, small populations of Pvalb$^+$ and Sst$^+$ INs appeared, suggesting that 3D environments promote their maturation (Figure 1E) (Mostajo-Radji et al., 2025; Walsh et al., 2025). 3D image reconstruction showed Tubb3$^+$ neuronal projections including GABA$^+$ and GABA$^-$ neurons (Figure 1F; Video S1).

Single-cell RNA sequencing (scRNAseq) of DF organoids at days 16, 30, and 60 yielded 17,970 transcriptomes across the 3 cell lines (Figure S3A). UMAP visualization identified neurons, progenitors, glia, and off-target non-neural cells (Figures S3B and S3C). Cellular diversity increased over time and remained consistent across lines (Figures S3D–S3F). Anchor-based label transfer to a developing mouse cortex reference (E10.5 to P4; Di Bella et al., 2021) confirmed alignment with *in vivo* cell types (Figures 1G, S3G, and S3H). Neural subclustering revealed diverse subtypes, including *Reelin*$^+$ Cajal-Retzius cells, *Slc17a6*$^+$ maturing PNs, *Crim1*$^+$ and *Lrrtm4*$^+$ corticofugal PNs, *Gad1*$^+$/*Gad2*$^+$/Sst$^+$ INs, *Stmn2*$^+$/*Map2*$^+$ non-forebrain neurons (~25%–30% of neurons), *Mfng*$^+$ intermediate progenitors, *Vim*$^+$/*Hes6*$^+$ radial glia, *Sox2*$^+$ progenitors (Figures 1H and S3I), suggesting robust forebrain specification across diverse genetic backgrounds.

Progressive network maturation in DF organoids

To characterize the progressive maturation of network activity patterns in DF organoids, we performed longitudinal extracellular recordings using high-density MEAs (Figure 2A). These arrays contain 26,400 recording sites and provide simultaneous readout from 1,024 channels, enabling network-level analysis with single-cell resolution.

Neural activity was analyzed across three developmental stages: early (days 23–33; 15 recordings; 3,678 putative neurons), intermediate (days 34–45; 55 recordings; 16,281 putative neurons), and late (days 46–64; 49 recordings; 10,037 putative neurons). Network function was quantified using two metrics: firing rate, reflecting single-neuron activity, and the spike-time tiling coefficient (STTC) with a 10 ms window, capturing pairwise temporal correlations independent of firing rate (Figures 2B–2D). Mean firing rates rose progressively, consistent with *in vivo* trends (Figure 2C) (Chini et al., 2022, 2024). STTC values increased in mean and variability over development, consistent with the emergence of structured, heterogeneous connectivity patterns (Figures 2D and 2E; Table S1). Both measures followed log-normal distributions, consistent with fundamental electrophysiological principles (Buzsáki and Mizuseki, 2014) (Figure S4). Maturation patterns were conserved across organoids from the 3 cell lines (Table S2). Some line-specific differences appeared at individual stages, but linear mixed-effects modeling revealed no significant divergence in developmental slopes for either firing rate or STTC (Figures 2F, 2G, and S5).

The progressive increase in synchronization contrasts with *in vivo* cortical development, where early network-wide synchrony transitions to sparse, decorrelated subnetwork activity (Chini et al., 2022, 2024; Golshani et al., 2009). This developmental decorrelation is thought to arise from the maturation and integration of inhibitory INs, which shift the excitatory-inhibitory balance toward inhibition (Chini et al., 2022; Wu et al., 2024). DF organoids contain relatively sparse IN populations, suggesting that insufficient inhibitory tone may prevent decorrelation. Similar sustained synchronization has been observed in human cortical organoids (Osaki et al., 2024; Trujillo et al., 2019; van der Molen et al., 2026), suggesting this may reflect a general constraint of organoid systems with limited IN content.

To investigate whether E-I balance influences network dynamics, we pharmacologically manipulated their synaptic transmission. Dimethyl sulfoxide (DMSO) vehicle control and 2-amino-5-phosphonovaleric acid (APV; NMDA receptor antagonist) had no significant effects on STTC (Tables S3 and S4; Figures S6A and S6B). In contrast, 2,3-dihydroxy-6-nitro-7-sulfamoyl-benzo[f]quinoxaline (NBQX; AMPA/Kainate antagonist) significantly reduced network connectivity ($p < 0.003$; Figure S6C; Table S4), consistent with the dominant role of AMPA receptors in cortical excitatory transmission. To examine inhibition, we blocked $GABA_A$ receptors with Gabazine, artificially elevating the E-I ratio (Figure 2H). Gabazine increased STTC significantly ($p < 0.001$) while leaving firing rates unchanged ($p = 0.79$; Figures 2I, 2J, and S6D; Tables S3 and S4), and prolonged burst durations (Figure S7). These results indicate that inhibitory tone modulates network synchronization in DF organoids.

Generation and characterization of VF organoids

To investigate the role of INs in network formation, we generated VF organoids by treating forebrain progenitors with Smoothened Agonist (SAG), a potent Sonic hedgehog (SHH) activator, during the first 14 days of differentiation

(G) Anchor-based label transfer mapping between primary tissue (developing mouse cerebral cortex) and organoid (DF organoids) datasets. DL CPN, deep layer callosal PN; UL CPN, upper layer callosal PN; SCPN, subcerebral PN; CThPN, corticothalamic PN; VLMC, vascular and leptomeningeal cells. $n = 17,970$ cells.
(H) UMAP visualization of neural lineage cell types in DF organoids. IPC, intermediate progenitor; RGC, radial glial cell. $n = 5,349$ cells. See also Figures S1–S3; Video S1.

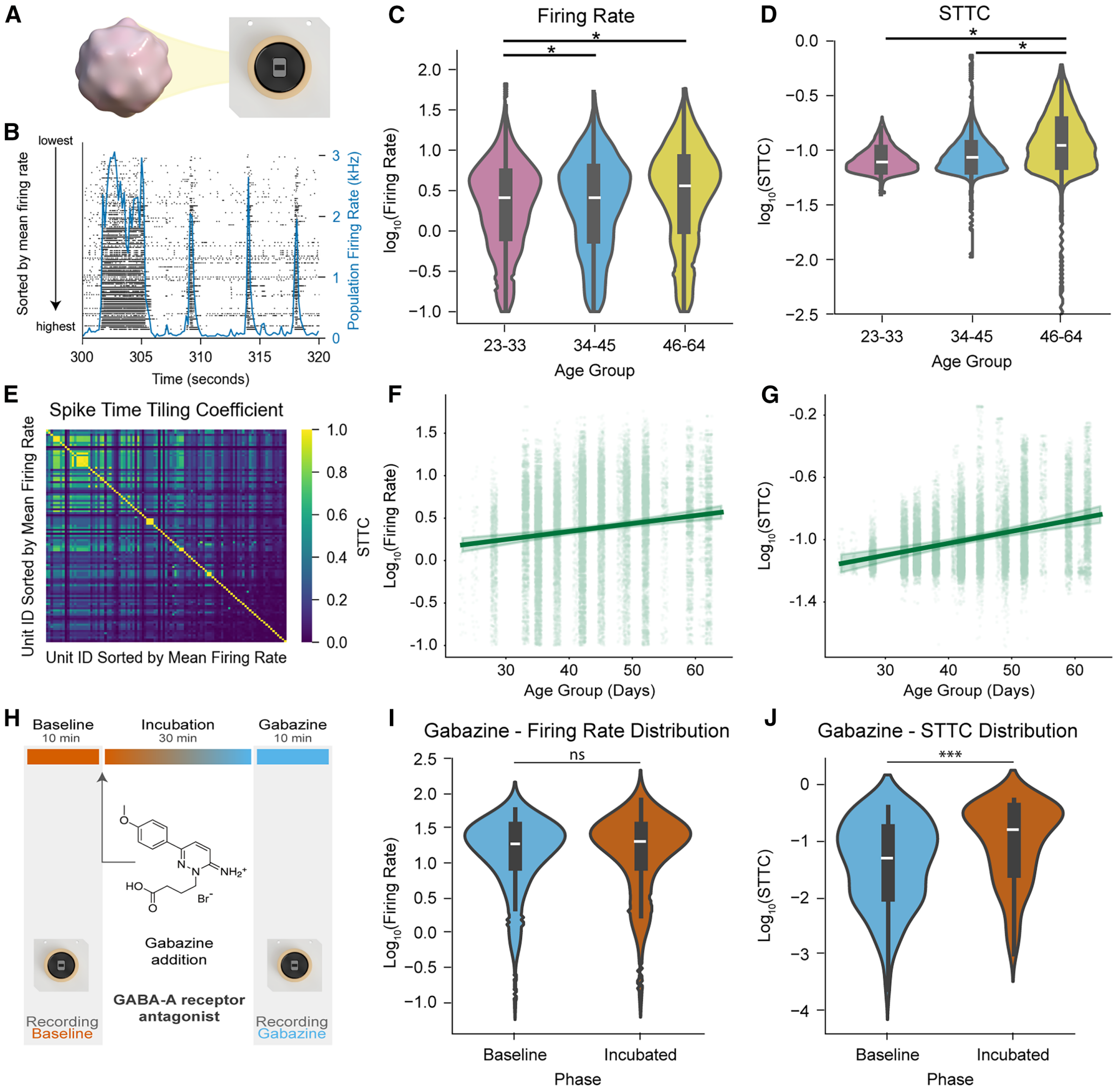

Figure 2. Neuronal activity and E-I balance in DF organoid networks

(A) Schematic of the MEA recording setup.

(B) Representative raster plot showing neuronal activity, with the population firing rate over time (blue). Units sorted by mean firing rate.

(C and D) Violin plots showing log transformed mean firing rates (Hz) (C) and log-transformed mean STTC (D) over early (23–33 days), mid (34–45 days), and late (46–64 days). $n = 16$ organoids, 28,809 units.

(E) STTC matrix showing pairwise spike train correlations, sorted by mean firing rate.

(F and G) Linear mixed-effects model predicted line plot of the log-transformed mean firing rate distribution (F) and log-transformed STTC (G). ns, not significant, * significant after Bonferroni correction, $p < 0.017$, mixed-effects model (D–G). Data shown as mean ± CI.

(H) Schematic of the recording protocol: 10-min baseline recording, 30-min drug incubation, and 10-min post-incubation recording.

(I and J) Violin plots showing (I) firing rates and (J) STTC distributions during baseline (blue) and post-Gabazine (orange). ($n = 3$ organoids, 133 units). ns, not significant, $***p < 0.0001$, mixed-effects models. See also Figures S4–S7; Tables S1–S4.

(Figures 3A and 3B). To further bias toward Pvalb$^+$ identity, we co-treated with MEK/ERK pathway inhibitor PD0325901 (Holter et al., 2021).

SAG treatment induced a significant shift in regional identity by day 10 while maintaining similar architecture and viability (Figures 1C, S8, and S9). VF organoid diameters showed some cell line variation early in development, with BRUCE4 being smaller than ES-E14TG2a and KH2 at days 1, 5, and 14 ($p < 0.05$; Figure S9A). However, overall organoid sizes were comparable between DF and VF types across development ($p > 0.5$; Figure 3C; Table S5). In addition, viability testing via acridine orange

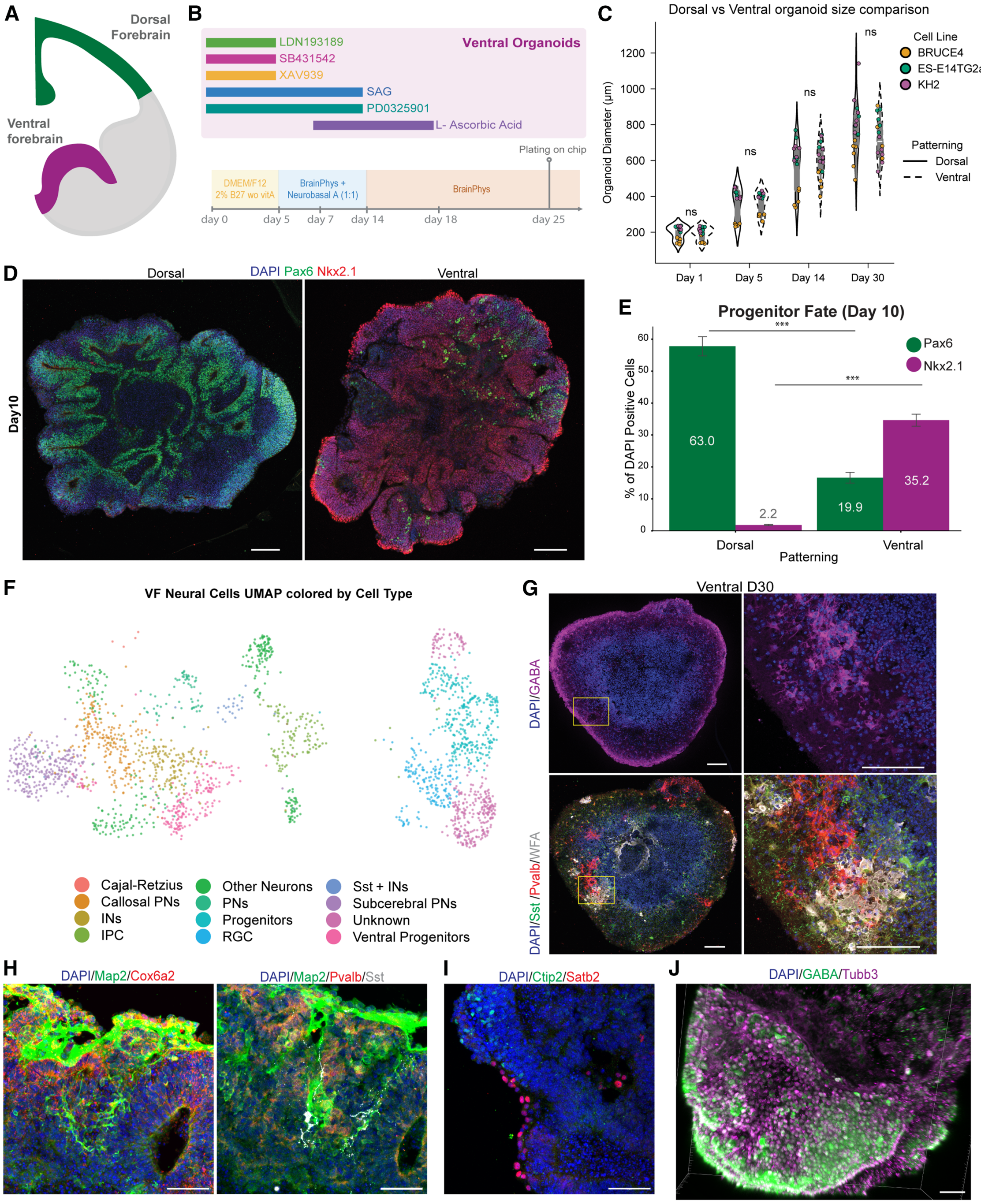

Figure 3. Characterization of the VF organoid development protocol

(A) Schematic representation of DF (green) and VF (purple) regions.

(B) Schematic of the protocol for VF organoid development.

(C) Organoid diameter over time for DF and VF organoids for all 3 cell lines. Student's *t* test, ns, not significant.

(D) IHC of day 10 organoids showing DF marker Pax6 (green) and VF marker Nkx2.1 (red). (n = 20 organoids from 4 different batches for both DF and VF).

(E) Quantification of $Pax6^+$ and $Nkx2.1^+$ cells across DF and VF patterned organoids. n = 20 organoids from 4 different batches for both DF and VF, $^{***}p < 0.0001$; Mann-Whitney U test. Data are shown as mean ± SEM.

(legend continued on next page)

(live) and propidium iodide (dead) was qualitatively similar across maturation stages (Figures S9B–S9E).

IHC quantification at day 10 showed Pax6 expression was higher in DF than VF organoids (DF = 57.78 ± 38.20%; VF = 16.64 ± 17.81%; $p < 0.001$), while Nkx2.1 was enriched in VF organoids (DF = 1.82 ± 2.12%; VF = 34.64 ± 20.06%; $p < 0.001$; Figures 3D, 3E, S10A, and S10B).

scRNAseq at days 16, 30, and 60 integrated 17,148 DF and 10,945 VF cells (Figures S10C–S10F), including neural populations and non-neural "other" cells from off-target lineages. When the neural population from VF organoids was reclustered and projected onto the same UMAP coordinates as DF organoids (Figure 1H), VF cells occupied the same major neural clusters, confirming that both organoid types generate comparable forebrain cell types (Figure 3F). To validate IN identity, we performed IHC on serial cryosections and confirmed strong GABA expression in the same regions as Pvalb^{+} and Sst^{+} cells (Figure 3G). Wisteria floribunda agglutinin (WFA) labeling revealed extensive perineuronal net formation surrounding Pvalb^{+} INs (Figure 3G), indicating functional maturation. Additionally, we validated the presence of the recently described Pvalb^{+} IN marker Cox6a2 (Mostajo-Radji et al., 2025) by IHC staining in different serial sections, showing extensive colocalization in regions of Pvalb expression (Figure 3H). We also confirmed that VF organoids generated the same PN subtypes as DF organoids, with Ctip2^{+} and Satb2^{+} cells present in qualitatively lower numbers (Figure 3I). Finally, 3D image reconstruction showed robust colocalization of GABA^{+}/Tubb3^{+} projections (Figure 3J; Video S2). Altogether, we conclude that VF organoids showed strong IN specification.

DF and VF organoids exhibit distinct network dynamics

To understand the contribution of INs to circuit formation in organoids, we compared the electrophysiological development of VF organoids and DF organoids at the same time points used for DF recordings (Figure 2). In VF organoids, log-transformed mean firing rates significantly increased from early to mid ($p < 0.001$) and early to late stages ($p < 0.002$), but not between mid and late development ($p = 0.76$; Figure 4A; Table S6).

When comparing firing rates between VF and DF organoids at matched time points (using data from Figure 2C), we found no significant differences at early or mid-stages ($p > 0.5$; Table S6). However, DF organoids displayed modest but statistically significant higher firing rates at late stages ($p = 0.02$; Table S6). Mixed-effects modeling of age-related changes in firing rates showed no significant difference in developmental slopes ($p = 0.74$) or intercepts ($p = 0.54$), indicating overall similar developmental dynamics between the two types of organoids (Figure 4B; Table S7).

In contrast, network synchrony as measured by STTC displayed a fundamentally different developmental trend. VF organoids remained relatively stable across development (Figure 4C), whereas DF organoids exhibited a steady increase (Figure 2D; Tables S6 and S7). Mixed-effects analysis confirmed a significant difference in the rate of change (slope) between DF and VF STTC values ($p = 0.04$), while intercepts were not significantly different ($p = 0.06$; Figure 4D; Tables S6 and S7). These results suggest that elevated inhibitory tone in VF organoids alters circuit refinement compared to DF organoids. However, unlike progressive decorrelation observed *in vivo* (Chini et al., 2024; Golshani et al., 2009; Wu et al., 2024), neither organoid model displayed continual synchrony reduction, pointing to the importance of sensory input or external factors to fully recapitulate developmental dynamics.

To validate that differences in IN composition underlie these divergent trajectories, we performed qPCR measuring the expression of *Gad2* (pan-GABAergic) and *Pvalb* relative to *Camk2a* (pan-excitatory) across the same three developmental stages. VF organoids consistently exhibited higher relative expression ratios for both *Gad2/Camk2a* and *Pvalb/Camk2a* compared to DF organoids at all time points (Figure S11). This molecular signature of elevated inhibitory content parallels the electrophysiological divergence in STTC trajectories, with VF organoids maintaining stable synchrony while DF organoids show progressive increases, consistent with the constraining effect of inhibitory tone on network correlation.

VF organoids develop stronger small-world topology through enhanced local clustering

In vivo, mouse cortical networks acquire small-world topology during early postnatal development before sensory experience (Chini et al., 2024; Hilgetag and Kaiser, 2004). To determine whether organoids recapitulate this

(F) UMAP visualization of neural populations from scRNA-seq in VF organoids. IPC, intermediate progenitor; RGC, radial glial cell. $n = 2{,}211$ cells.
(G) IHC of day 30 VF organoids showing: (top) GABA (magenta); (bottom) Sst (green), Pvalb (red), and WFA (gray) in the following section.
(H) IHC of day 30 VF organoids showing: (left) Map2 (green), Cox6a2 (red); (right) Map2 (green), Pvalb (red), and Sst (gray) in the following section.
(I) IHC of day 30 VF organoids showing Ctip2 (green) and Satb2 (red).
(J) 3D reconstruction of day 40 VF organoid stained for GABA (green) and Tubb3 (magenta). DAPI nuclear counterstain is shown in blue. Scale bars, 50 μm. See also Figures S8–S10; Video S2; Table S5.

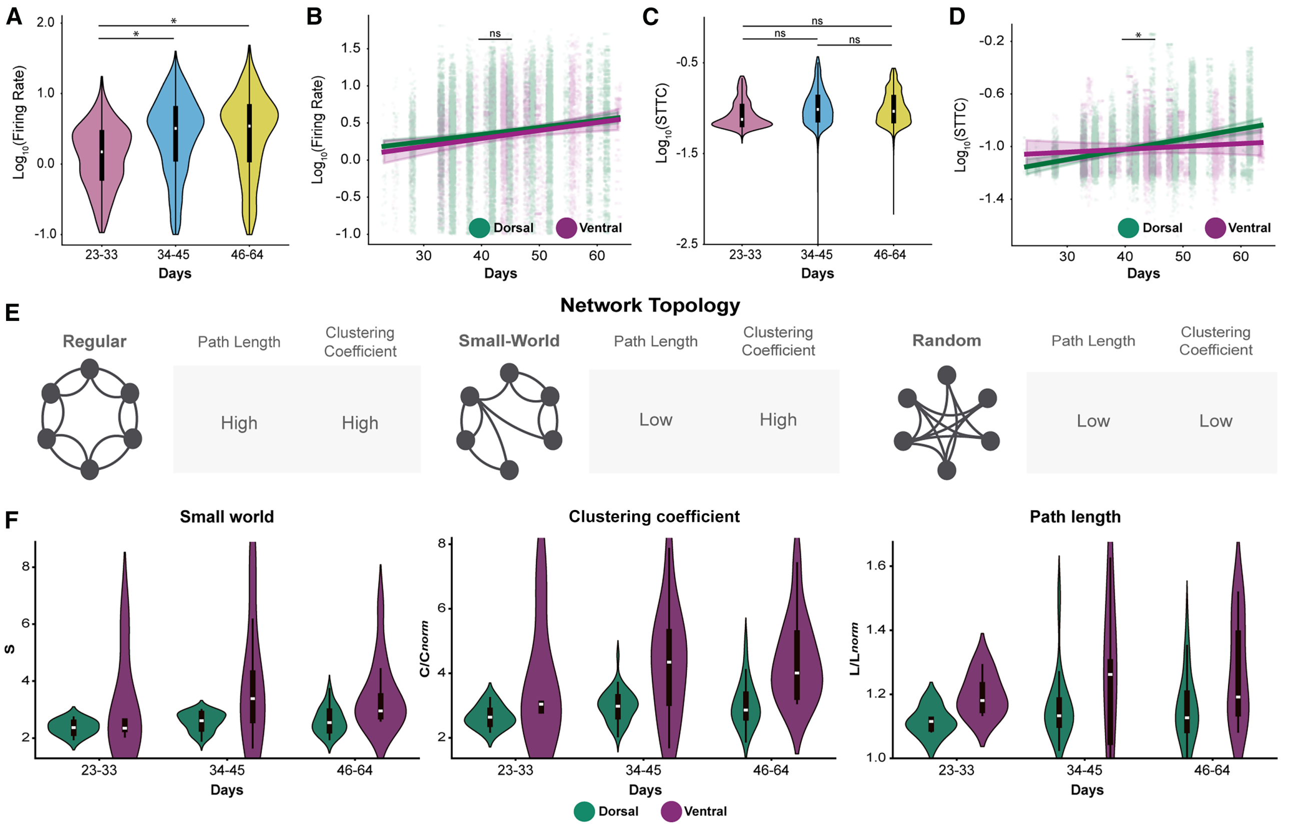

Figure 4. DF and VF organoids follow distinct developmental trajectories in neural dynamics
(A) Violin plots showing the distribution of log-transformed firing rates across three developmental stages in organoids: 23–33 days (pink), 34–45 days (blue), and 46–64 days (yellow). $n = 18$ organoids, 7,489 units.
(B) Scatterplot with regression lines (LME) showing the relationship between log-transformed firing rate (y axis) and age in days (x axis) for DF (green; $n = 16$ organoids, 28,809 units) and VF (purple; $n = 18$ organoids, 7,489 units) organoids. Individual data points represent recorded units. "ns" indicates non-significant difference between the slopes of DF and VF.
(C) Same as (A) but for difference between log-transformed STTC values.
(D) Same as (B) but for difference between the log-transformed STTC slopes. ns, not significant, $*p < 0.017$, $**p < 0.003$, $***p < 0.0003$ (Bonferroni corrected in A and C), mixed-effects model.
(E) Schematic representations of different network topologies: regular (top), small-world (middle), and random (bottom).
(F) Violin plots showing the distribution of (left) small-world index (S) for DF (green; $n = 16$ organoids) and VF (purple; $n = 18$ organoids), (middle) clustering coefficient (C), and (right) path length (L). ns, not significant, $*p < 0.05$, $**p < 0.01$, $***p < 0.001$, mixed-effects model. See also Figure S11; Tables S6–S10.

trajectory and whether cellular composition influences topology, we analyzed small-world organization across DF and VF development.

The small-world index ($S = C_{norm}/L_{norm}$) quantifies the balance between local clustering (C) and path length (L) (Bassett and Bullmore, 2017) (Figure 4E). Values significantly greater than 1 indicate small-world organization. For each recording, we generated 1,000 surrogate datasets by shuffling neuron IDs while preserving firing rates (Okun et al., 2012; 2015). STTC values exceeding the 90th percentile were included in binary adjacency matrices. S values increased progressively in both organoid types (Tables S8 and S9). VF organoids showed significantly higher S than DF at all stages ($p < 0.003$ in all cases; Figure 4F). Analysis of L_{norm} and C_{norm} revealed that C_{norm} is the primary determinant of this difference ($p < 0.001$). VF organoids exhibited significantly higher C_{norm}, particularly at late stages ($p < 0.003$; Table S10). This enhanced clustering is consistent with *in vivo* observations where inhibitory integration increases clustering coefficients, suggesting the elevated inhibitory content in VF organoids is associated with enhanced topological organization.

Divergent network specialization in DF and VF organoids

During cortical development *in vivo*, networks transition from diffuse synchrony to hierarchical core-periphery

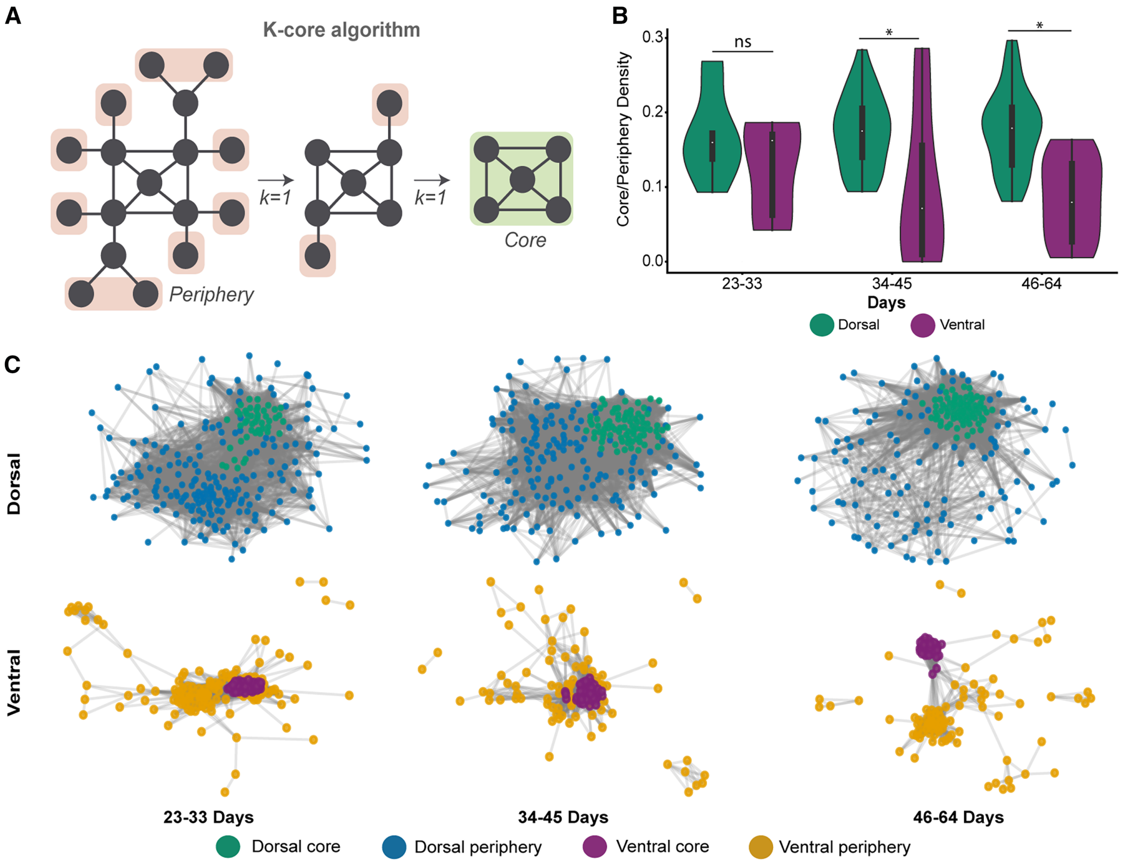

Figure 5. Divergent core-periphery organization reveals distinct network specialization in DF and VF organoids

(A) Schematic of the *k*-core algorithm used to identify core and peripheral regions within neural networks.

(B) Violin plots showing core and periphery density across developmental stages (23–33; 34–45; and 46–64 days) for DF (green; $n = 16$ organoids) and VF (purple; $n = 18$ organoids) organoids. ns, not significant; $^*p < 0.05$, mixed-effects model.

(C) Representative force-directed graph visualizations of core/periphery labeled nodes per age group DF core (dark green; $n = 46, 79, 73$, respectively), DF periphery (blue; $n = 195, 165, 116$, respectively), (bottom) VF core (purple; $n = 21, 22, 22$, respectively), and VF periphery (yellow; $n = 53, 66, 63$) regions.

organization (Bassett et al., 2013). We applied *k*-core decomposition to assess whether organoids exhibit similar specialization (Lahav et al., 2016) (Figure 5A).

Core-periphery connectivity showed no differences at early stages (days 23–33: DF = 0.17 ± 0.02; VF = 0.13 ± 0.03; $p = 0.54$). However, DF organoids exhibited significantly higher core-periphery interaction at intermediate (DF = 0.17 ± 0.01; VF = 0.10 ± 0.03; $p < 0.001$) and late stages (DF = 0.18 ± 0.01; VF = 0.08 ± 0.02; $p < 0.001$; Figure 5B). DF networks maintain globally integrated architecture, while VF networks progressively adopt segregated, modular structures (Figure 5C). This parallels *in vivo* observations where inhibitory maturation drives functional specialization (Wu et al., 2024), suggesting IN enrichment enables progression toward mature cortical organization with functionally distinct communities.

DF and VF organoids develop distinct hub-based organization

Hub neurons with disproportionate connectivity coordinate population activity during development (Bollmann et al., 2023; Mòdol et al., 2017). To investigate whether a similar phenomenon occurs in organoids, we calculated composite hubness scores incorporating degree, strength, betweenness, and closeness centrality (Figure 6A).

DF organoids formed densely interconnected networks with distributed hubs. VF organoids developed segregated clusters with localized hubs (Figures 6B, S12, and S13). STTC matrices sorted by hubness revealed broadly distributed synchrony in DF organoids versus spatially cohesive hub clusters in VF organoids (Figures 6C, S12, and S13). VF clusters emerged early and became more refined by late development, coinciding with increased modularity (23–33 days: $p = 0.298$; 34–45 days: $p = 0.007$;

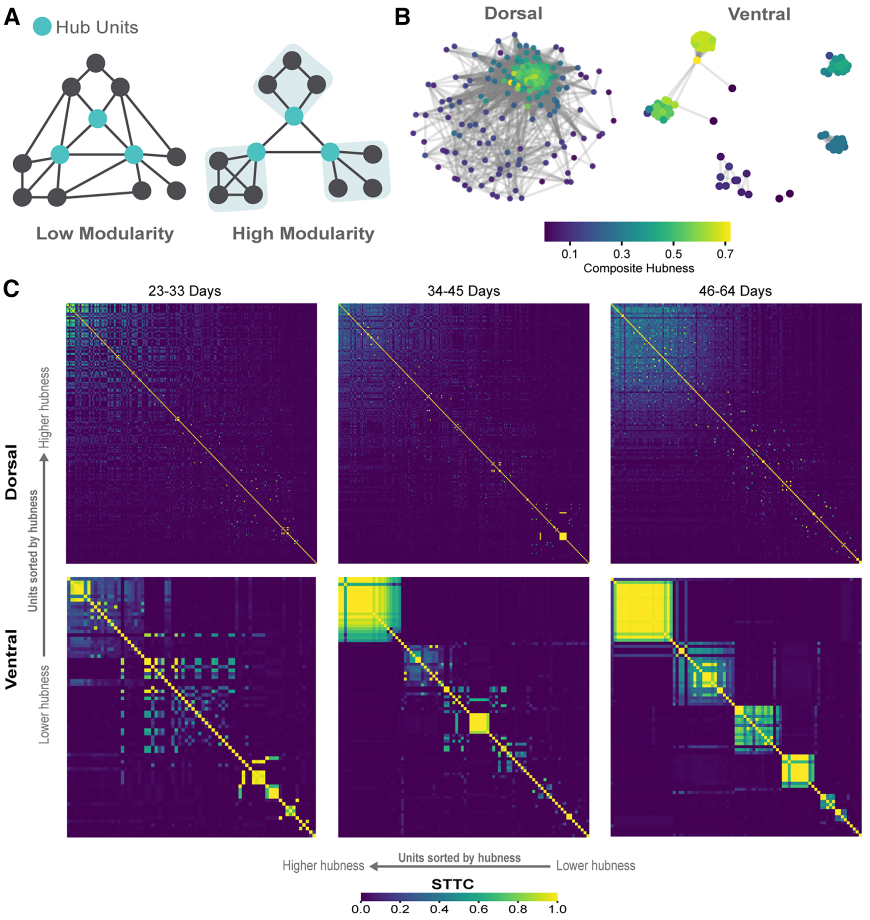

Figure 6. Network modularity dynamics distinguish dorsal and ventral forebrain organoid development

(A) Schematics illustrating network modularity, comparing low and high modularity states and highlighting the role of high-hub units.

(B) Comparison of examples between DF (n = 194 units) and VF (n = 87 units) forebrain organoids at the mature stage (46–64 days).

(C) STTC matrix of units sorted by hubness score. See also Figures S12–S14; Table S11.

46–64 days: $p = 0.002$; Figure S14; Table S11). The spatially refined hub organization in VF organoids is consistent with *in vivo* principles where inhibitory neurons are associated with modular specialization (Hensch, 2005; Contractor et al., 2021).

Distinct core-periphery dynamics underpin developmental specialization in DF and VF organoids

In vivo, developing networks exhibit sequential activation patterns that refine as inhibitory circuits mature (Chini et al., 2022; Wu et al., 2024). We analyzed "*backbone*" units (neurons firing at least twice during 90% of network bursts) that form stable cores supporting sequential activity (van der Molen et al., 2026). Backbone unit proportions showed no early ($p = 0.86$) or intermediate stage differences ($p = 0.16$). By late stages, DF organoids showed significantly higher rigidity ($p < 0.001$; Figure S15; Table S12).

Louvain community detection (Schuurman and Bruner, 2024) revealed DF organoids exhibited higher burst-to-burst correlation (DF = 0.24 ± 0.01; VF = 0.19 ± 0.01; $p = 0.001$) and more regular temporal structure (DF = 95.2 ± 0.9 ms; VF = 94.0 ± 1.4 ms; $p = 0.01$), indicating stable ensemble recruitment (Figure 7; Table S13). VF organoids showed distributed, variable patterns. These organizational differences reflect complementary computational strategies. The stable patterns in DF organoids resemble early developmental states with dominant excitatory drive (Chini et al., 2022), while the variable dynamics in VF organoids parallel observations where elevated inhibitory tone enables flexible ensemble recruitment.

DISCUSSION

Our study shows that mouse forebrain organoids can self-organize into complex neural networks that recapitulate several organizational principles of cortical development. By generating regionalized DF and VF organoids with distinct cellular compositions, we systematically evaluated how E-I balance shapes network dynamics and topology. Both organoid types contain similar neuronal subtypes at different ratios, with VF organoids enriched in $Pvalb^+$ INs. The emergence of small-world architecture in both models indicates that intrinsic developmental programs are

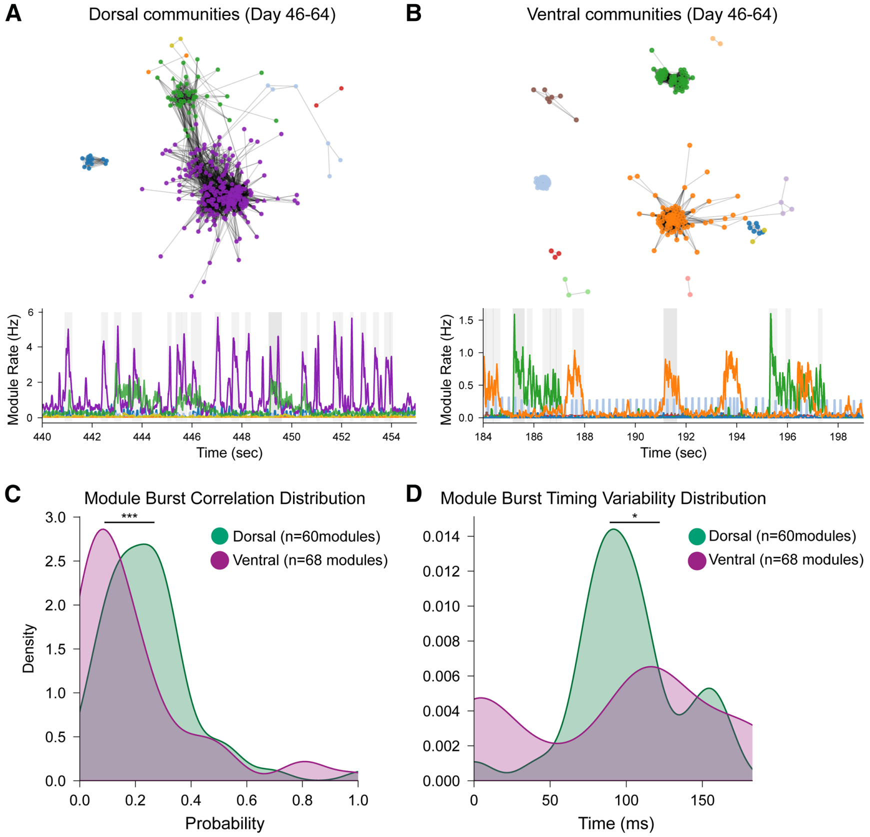

Figure 7. Functional community structure reveals differences in network organization between DF and VF organoids
(A) DF organoids (46–64 days) exhibit a densely integrated community structure. Top: force-directed graph of the STTC-derived network, with node colors indicating module membership. Bottom: representative time-series showing synchronized activity across modules; module 4 (green) and module 6 (red) display highly correlated bursts. $n = 7$ modules.
(B) VF organoids at the same stage show a more segregated structure. Top: network visualization reveals reduced inter-module connectivity. Bottom: module activity patterns show distinct temporal signatures with less correlation between different functional communities. $n = 10$ modules.
(C) Distribution of module burst correlations shows a higher probability of synchronized bursting in DF (green; $n = 16$ organoids) compared to VF (purple; $n = 18$ organoids) organoids.
(D) Distribution of burst timing variability indicates broader temporal spread in VF (purple; $n = 18$ organoids) modules, while DF (green; $n = 16$ organoids) modules exhibit tighter, more synchronized timing. $^*p < 0.05$, $^{***}p < 0.0001$, Kolmogorov-Smirnov test. See also Figure S15; Tables S12 and S13.

sufficient to assemble complex network architectures even without sensory input (Chini et al., 2024; Hilgetag and Kaiser, 2004).

The progressive synchronization in DF organoids contrasts with *in vivo* decorrelation (Chini et al., 2022; Golshani et al., 2009), yet aligns with predictions from the E-I balance hypothesis, which posits that insufficient inhibitory tone prevents network decorrelation. DF organoids contain sparse IN populations, and our pharmacological and qPCR experiments suggest that GABAergic inhibition constrains synchronization. VF organoids enriched with Pvalb$^+$ INs exhibit stable STTC trajectories. Importantly, while the observed differences in network maturation strongly correlate with inhibitory neuron enrichment, establishing causality between specific cell type proportions and these network properties will require future targeted manipulations, such as optogenetic or chemogenetic perturbations. Sustained synchronization in human cortical organoids (Mancinelli et al., 2025; Osaki et al., 2024; Trujillo et al., 2019; van der Molen et al., 2026) suggests this reflects a general constraint of systems with limited IN content. While neither model fully recapitulates *in vivo* decorrelation, likely requiring sensory inputs or long-range connectivity (Antón-Bolaños et al., 2018; Dorrn et al., 2010), the divergent trajectories reveal how cellular composition shapes network maturation independently of external cues. Comparable developmental trajectories of synchrony and desynchronization have been documented *ex vivo* using brain slices (Wu et al., 2024), providing a reference framework for interpreting organoid network maturation.

To contextualize these findings, we note that *in vivo* mouse neocortex contains approximately 15%–20% GABAergic interneurons, with Pvalb$^+$ cells comprising roughly 40% of this population (Hensch, 2005). Our DF organoids, with sparse interneuron content, fall below this physiological range, while VF organoids likely exceed it. This positions *in vivo* neocortex between our two model systems along the E-I balance spectrum, supporting our interpretation that DF and VF organoids bracket the physiological state and provide complementary platforms for mechanistic investigation.

By establishing reproducible protocols across three genetically distinct mESC lines, we provide a platform to

investigate how cellular composition governs circuit assembly. Because DF and VF organoids represent two points along a spectrum of neocortical E-I balance, organoids derived from mouse models of neurodevelopmental disorders with disrupted E-I balance would likely exhibit network topologies intermediate between these extremes, providing testable predictions that can be validated *in vivo* using genetically identical controls. While patient-derived iPSC organoids link to clinical phenotypes, mouse organoids uniquely enable mechanistic testing through causal perturbations, longitudinal recordings, and direct comparison to circuit and behavioral data from mice derived from the same mESC lines. Key organizational principles appear conserved across mammals (Bassett and Bullmore, 2017), though developmental timescales and IN composition differ between species (Krienen et al., 2020). In humans, understanding early network development is primarily inferred from neonatal EEGs showing emerging oscillatory patterns (Chini et al., 2022), but regional comparisons at cellular resolution remain inaccessible due to technical and ethical constraints. Forebrain organoids thus provide a tractable system to rigorously test hypotheses against well-defined *in vivo* benchmarks.

Several limitations should be considered when interpreting our findings. Although organoids recapitulate core features of network development, they lack essential *in vivo* characteristics such as vascularization and complete cellular diversity, including Vip$^+$ INs and microglia that contribute to synaptic pruning (Velasco et al., 2019). Our organoids also contain off-target non-neural cell populations, as revealed by scRNAseq, at proportions comparable to those reported in other mouse organoid systems (Lindhout et al., 2025; Medina-Cano et al., 2022; Sánchez et al., 2024). Planar MEAs primarily record from surface neurons, which may bias our network analyses (Tanveer et al., 2025), and do not fully capture 3D circuit architecture. The self-contained nature of organoids also precludes studying how sensory inputs or long-range projections influence circuit development. Additionally, our qPCR analysis of PN/IN proportions required destructive sampling, preventing direct correlation with electrophysiological measurements at the individual organoid level. Non-destructive molecular profiling methods, such as extracellular vesicle analysis or fluorescent reporter lines, would enable such correlations in future studies. Future work incorporating additional cell types, 3D MEAs or optical recording technologies, and patterned stimulation paradigms will be necessary to investigate input-dependent maturation and more fully model *in vivo* developmental dynamics.

Despite these limitations, our findings establish mouse forebrain organoids as a powerful platform for dissecting how cellular composition governs cortical network assembly. By showing that E-I balance fundamentally shapes network topology in the absence of sensory input, we provide a tractable system to investigate the intrinsic developmental logic underlying circuit formation and its disruption in neurodevelopmental disorders.

METHODS

mESC lines

We used three male mESC lines: BRUCE4 (RRID:CVCL_K037), ES-E14TG2a (RRID:CVCL_Y481), and KH2 (RRID:CVCL_C317). Detailed maintenance protocols are described in the supplemental methods.

GMEM-based DF organoids generation

DF organoids were generated using a modified version of our previous protocol (Elliott et al., 2023). Full procedures are provided in supplemental methods.

DMEM-based DF organoids generation

mESCs were dissociated into single cells using TrypLE Express Enzyme (Thermo Fisher Scientific [TFS] #12604021) for 5 min at 37°C, then re-aggregated in Lipidure-coated 96-well V-bottom plates at 3,000 cells per well in 150 μL of mESC maintenance medium supplemented with 10 μM Rho Kinase Inhibitor Y27632 (Tocris #1254) and 1,000 U/mL Recombinant Mouse Leukemia Inhibitory Factor (LIF) (Millipore Sigma [MS] #ESG1107).

After 24 h, the medium was replaced with forebrain patterning medium consisting of Dulbecco's Modified Eagle Medium/Nutrient Mixture F-12 (DMEM/F12) with GlutaMAX (TFS #10565018), 1X chemically defined (CD) lipid concentrate (TFS #11905031), 0.1 mM MEM non-essential amino acids (NEAAs; TFS #11140050), 1 mM sodium pyruvate (MS #S8636), 1X N2 supplement (TFS #17502048), and 2X B27 minus vitamin A (-VitA; TFS #12587010). Supplements included 10 μM Y27632, 5 μM XAV939 (StemCell Technologies (SCT) #1001052), and 5 μM SB431542 (Tocris #1614). Medium was changed daily, with N2 and B27 added post-filtration.

On day 5, organoids were transferred to ultralow adhesion plates (MS #CLS3471) with neuronal differentiation medium and placed on an orbital shaker at 68 rpm.

From days 6–12, progenitor expansion medium included a 1:1 mix of Neurobasal-A and BrainPhys media (SCT #05790), supplemented with B27 -VitA, N2 supplement, MEM NEAAs, CD lipid concentrate, and 200 μM ascorbic acid (Sigma Aldrich [SA] #49752). Medium was changed every 2–3 days under 5% CO_2.

From day 13 onward, neural maturation medium consisted of BrainPhys medium supplemented with B27 Plus (TFS #A3582801), CD lipid concentrate, and 5 μg/mL

heparin (SA #H3149). Ascorbic acid was included until day 25. Medium was changed every 2–3 days, with organoids maintained at 60 rpm (16 per well) to minimize fusion. Primocin (0.05 mg/mL; InvivoGen #antpm05) was included in all media throughout the protocol.

VF organoids generation

VF organoids were generated using the same protocol as DF organoids with the following modifications: 250 nM LDN193189 (SCT #72147) was added from days 0–5, and 100 nM PD0325901 (SCT #72184) and 100 nM SAG (MS #SIAL-SML1314) were included from days 0–14.

scRNAseq

Organoids were dissociated using the Worthington Papain Dissociation System (#LK003150) following manufacturer instructions. Libraries were prepared using the PIPseq T2 Single Cell RNA v4.0PLUS platform (Fluent #FBSSCRT28V4.05) or Illumina Single Cell 3′ RNA Prep, T2 (Illumina #20135689), pooling 3,333 cells per genotype (10,000 total per library).

Sequencing was performed on an AVITI PE75 flowcell. Data were processed with PIPseeker, aligned to GRCm39, and analyzed in Seurat using standard quality control, batch correction, clustering, and annotation based on reference brain atlases. Full protocols are provided in supplemental methods.

IHC and imaging

For detailed IHC conditions, table of antibodies, and additional analysis information, see supplemental methods. Imaging was performed using either: Zeiss 880 confocal microscope with Airyscan Fast or Zeiss AxioImager Z2 widefield microscope, with acquisition via Zen Blue software and analysis in Zen Black/ImageJ.

Electrophysiology

Day 25 organoids were plated on MaxOne MEAs (Maxwell Biosystems #PSM). Neuronal activity was recorded every 2–3 days at 20 kHz from the 1,020 most active electrodes under standard incubation conditions (5% CO_2, 37°C). Recordings were filtered, spike-sorted with Kilosort2, and quality controlled. For pharmacology, activity in 60- to 65-day-old DF organoids was recorded before and after treatment with Gabazine (1 μM), NBQX (20 μM), or APV (100 μM). Full methods are detailed in the supplemental methods.

Quantifications and statistical analyses

Statistical analyses were performed in Python. Details on statistical tests, sample sizes, and p values are provided in the figure legends. Significance was defined as $p < 0.05$, with corrections for multiple comparisons applied when appropriate.

Spike train and network analysis

To quantify neuronal synchrony and population-level organization, we computed pairwise STTC using a 10 ms window and constructed functional connectivity matrices based on significance thresholds derived from spike-shuffled surrogate data. Binary networks were analyzed for clustering, path length, small-worldness, and hub metrics using custom Python pipelines. Full computational methods, including STTC definition, surrogate-based thresholding, and network metrics, are provided in the supplemental methods.

RESOURCE AVAILABILITY

Lead contact

Requests for further information and resources should be directed to and will be fulfilled by the lead contact, Mohammed A. Mostajo-Radji (mmostajo@ucsc.edu).

Materials availability

This study did not generate new reagents.

Data and code availability

The accesion numbers for the scRNAseq data reported in this paper is GEO: GSE290330, GSE312396, and the scRNAseq data is also available in the UCSC Cell Browser: https://mouse-df-vf-organoid.cells.ucsc.edu, The accession number for the MEA data is DANDI: 001374. All the code is available in GitHub: https://github.com/braingeneers/Sakura_final.

ACKNOWLEDGMENTS

We thank the Colquitt lab for support with scRNA-seq library preparation, Kristof Tigyi for sharing mESC lines, and Tomasz Nowakowski for valuable manuscript feedback. We acknowledge the National Research Platform (NSF CNS1730158, ACI1540112, ACI1541349, OAC1826967), the University of California Office of the President (UCOP), and the UCSD Calit2/Qualcomm Institute. Sequencing was performed at the UC Davis Genome Center (RRID: SCR_012480, NIH 1S10OD010786). Imaging was provided by the UCSC Microscopy Core (RRID: SCR_021135).

This work was supported by Schmidt Futures (SF857 to S.R.S., D.H., and M.T.); the National Human Genome Research Institute (RM1HG011543 to S.R.S., D.H., and M.T.); the National Science Foundation (NSF; 2134955 to S.R.S., D.H., and M.T.; 2034037 to M.T.; 2515389 to T.S., D.H., M.T., and M.A.M.-R.); the National Institute of Mental Health (U24MH132628 to D.H. and M.A.M.-R.); the National Institute of Neurological Disorders and Stroke (U24NS146314 to D.H. and M.A.M.-R.); the California Institute for Regenerative Medicine (CIRM) (DISC4-16285 to S.R.S., M.T., and M.A.M.-R.; DISC4-16337 to M.A.M.-R.); UCOP(M25PR9045 to S.R.S., M.T., and M.A.M.-R.); and the Brain and Behavior Research Foundation (33184 to M.A.M.-R.). H.E.S. was supported by NSF Graduate Research Fellowship Program (GRFP), S.H. by University of California - Hispanic Serving Institutions Doctoral Diversity Initiative (UC-HSI DDI), and F.R. by CIRM Bridges Program. The content is solely the responsibility of the authors and does not necessarily represent the official views of the National Institutes of

Health, the National Science Foundation, the University of California, CIRM, or any other agency of the State of California.

AUTHOR CONTRIBUTIONS

S.H., H.E.S., M.T., and M.A.M.-R. conceptualized the project; S.H., H.E.S., I.C., G.A.K., A.R., D.S., S.V.-C., J.G., T.v.d.M., F.R., C.N.A., and K.V. conducted the experiments; M.C., M.R., S.R.S., B.M.C., T.S., D.H., M.T., and M.A.M.-R. provided supervision and secured funding; S.H., H.E.S., and M.A.M.-R. wrote the manuscript with input from all authors.

DECLARATION OF INTERESTS

K.V. is a co-founder, and D.H., S.R.S., and M.T. are advisory board members of Open Culture Science, Inc. A.R. is co-founder and CTO of Immergo Labs. H.E.S. and M.A.M.-R. are inventors on a patent application for brain organoid generation; M.A.M.-R. is also listed on patent applications for electrophysiology analysis and Pvalb^{+} IN generation, and advises Atoll Financial Group and Optimal.

DECLARATION OF GENERATIVE AI AND AI-ASSISTED TECHNOLOGIES IN THE WRITING PROCESS

During the preparation of this work, the authors used ChatGPT, Gemini, and Claude to improve language clarity and readability. All content was reviewed and edited by the authors, who take full responsibility for the final version.

SUPPLEMENTAL INFORMATION

Supplemental information can be found online at https://doi.org/10.1016/j.stemcr.2026.102832.

Received: June 3, 2025
Revised: February 2, 2026
Accepted: February 3, 2026
Published: March 5, 2026

REFERENCES

Achard, S., Salvador, R., Whitcher, B., Suckling, J., and Bullmore, E. (2006). A resilient, low-frequency, small-world human brain functional network with highly connected association cortical hubs. J. Neurosci. *26*, 63–72.

Antón-Bolaños, N., Espinosa, A., and López-Bendito, G. (2018). Developmental interactions between thalamus and cortex: a true love reciprocal story. Curr. Opin. Neurobiol. *52*, 33–41.

Antonello, P.C., Varley, T.F., Beggs, J., Porcionatto, M., Sporns, O., and Faber, J. (2022). Self-organization of *in vitro* neuronal assemblies drives to complex network topology. eLife *11*, e74921.

Alam El Din, D.M., Moenkemoeller, L., Loeffler, A., Habibollahi, F., Schenkman, J., Mitra, A., van der Molen, T., Ding, L., Laird, J., Schenke, M., et al. (2025). Human neural organoid microphysiological systems show the building blocks necessary for basic learning and memory. Commun. Biol. *8*, 1237.

Bassett, D.S., and Bullmore, E.T. (2017). Small-World Brain Networks Revisited. Neuroscientist *23*, 499–516.

Bassett, D.S., Wymbs, N.F., Rombach, M.P., Porter, M.A., Mucha, P.J., and Grafton, S.T. (2013). Task-Based Core-Periphery Organization of Human Brain Dynamics. PLoS Comput. Biol. *9*, e1003171.

Bollmann, Y., Modol, L., Tressard, T., Vorobyev, A., Dard, R., Brustlein, S., Sims, R., Bendifallah, I., Leprince, E., de Sars, V., et al. (2023). Prominent *In Vivo* Influence of Single Interneurons in the Developing Barrel Cortex. Nat. Neurosci. *26*, 1555–1565.

Buzsáki, G., and Mizuseki, K. (2014). The Log-Dynamic Brain: How Skewed Distributions Affect Network Operations. Nat. Rev. Neurosci. *15*, 264–278.

Chini, M., Pfeffer, T., and Hanganu-Opatz, I. (2022). An Increase of Inhibition Drives the Developmental Decorrelation of Neural Activity. eLife *11*, e78811.

Chini, M., Hnida, M., Kostka, J.K., Chen, Y.N., and Hanganu-Opatz, I.L. (2024). Preconfigured Architecture of the Developing Mouse Brain. Cell Rep. *43*, 114267.

Contractor, A., Ethell, I.M., and Portera-Cailliau, C. (2021). Cortical interneurons in autism. Nat. Neurosci. *24*, 1648–1659.

Crocco, E., Iannello, L., Tonelli, F., Lagani, G., Pandolfini, L., Ferro, M., Amato, G., Di Garbo, A., and Cremisi, F. (2025). A Proper Excitatory/Inhibitory Ratio Is Required to Develop Synchronized Network Activity in Mouse Cortical Cultures. Stem Cell Rep. *20*, 102646.

Di Bella, D.J., Habibi, E., Stickels, R.R., Scalia, G., Brown, J., Yadollahpour, P., Yang, S.M., Abbate, C., Biancalani, T., Macosko, E.Z., et al. (2021). Molecular Logic of Cellular Diversification in the Mouse Cerebral Cortex. Nature *595*, 554–559.

Dorrn, A.L., Yuan, K., Barker, A.J., Schreiner, C.E., and Froemke, R.C. (2010). Developmental sensory experience balances cortical excitation and inhibition. Nature *465*, 932–936.

Downes, J.H., Hammond, M.W., Xydas, D., Spencer, M.C., Becerra, V.M., Warwick, K., Whalley, B.J., and Nasuto, S.J. (2012). Emergence of a small-world functional network in cultured neurons. PLoS Comput. Biol. *8*, e1002522.

Eiraku, M., Watanabe, K., Matsuo-Takasaki, M., Kawada, M., Yonemura, S., Matsumura, M., Wataya, T., Nishiyama, A., Muguruma, K., and Sasai, Y. (2008). Self-Organized Formation of Polarized Cortical Tissues from ESCs and Its Active Manipulation by Extrinsic Signals. Cell Stem Cell *3*, 519–532.

Elliott, M.A.T., Schweiger, H.E., Robbins, A., Vera-Choqqueccota, S., Ehrlich, D., Hernandez, S., Voitiuk, K., Geng, J., Sevetson, J.L., Core, C., et al. (2023). Internet-Connected Cortical Organoids for Project-Based Stem Cell and Neuroscience Education. eNeuro *10*, ENEURO.0308-23.2023.

Ferdaos, N., Lowell, S., and Mason, J.O. (2022). Pax6 mutant cerebral organoids partially recapitulate phenotypes of Pax6 mutant mouse strains. PLoS One *17*, e0278147.

Golshani, P., Gonçalves, J.T., Khoshkhoo, S., Mostany, R., Smirnakis, S., and Portera-Cailliau, C. (2009). Internally Mediated Developmental Desynchronization of Neocortical Network Activity. J. Neurosci. *29*, 10890–10899.

Hensch, T.K. (2005). Critical period plasticity in local cortical circuits. Nat. Rev. Neurosci. *6*, 877–888.

Hilgetag, C.C., and Kaiser, M. (2004). Clustered Organization of Cortical Connectivity. Neuroinformatics *2*, 353–360.

Holter, M.C., Hewitt, L.T., Nishimura, K.J., Knowles, S.J., Bjorklund, G.R., Shah, S., Fry, N.R., Rees, K.P., Gupta, T.A., Daniels, C.W., et al. (2021). Hyperactive MEK1 Signaling in Cortical GABAergic Neurons Promotes Embryonic Parvalbumin Neuron Loss and Defects in Behavioral Inhibition. Cereb. Cortex *31*, 3064–3081.

Krienen, F.M., Goldman, M., Zhang, Q., CH del Rosario, R., Florio, M., Machold, R., Saunders, A., Levandowski, K., Zaniewski, H., Schuman, B., et al. (2020). Innovations present in the primate interneuron repertoire. Nature *586*, 262–269.

Lahav, N., Ksherim, B., Ben-Simon, E., Maron-Katz, A., Cohen, R., and Havlin, S. (2016). K-shell decomposition reveals hierarchical cortical organization of the human brain. New J. Phys. *18*, 083013.

Li, Y., Mao, X., Zhou, X., Su, Y., Zhou, X., Shi, K., and Zhao, S. (2020). An Optimized Method for Neuronal Differentiation of Embryonic Stem Cells *in Vitro*. J. Neurosci. Methods *330*, 108486.

Liao, X., Vasilakos, A.V., and He, Y. (2017). Small-world human brain networks: perspectives and challenges. Neurosci. Biobehav. Rev. *77*, 286–300.

Lindhout, F.W., Szafranska, H.M., Imaz-Rosshandler, I., Guglielmi, L., Moarefian, M., Voitiuk, K., Zernicka-Glover, N.K., Boulanger, J., Schulze, U., Sánchez, D.J.L., et al. (2025). Calcium Dynamics Tune Developmental Tempo to Generate Evolutionarily Divergent Axon Tract Lengths. Preprint at bioRxiv. https://doi.org/10.1101/2024.12.28.630576.

Mall, E.M., Herrmann, D., and Niemann, H. (2017). Murine pluripotent stem cells with a homozygous knockout of Foxg1 show reduced differentiation towards cortical progenitors in vitro. Stem Cell Res. *25*, 50–60.

Mancinelli, S., Bariselli, S., and Lodato, S. (2025). The emergence of electrical activity in human brain organoids. Stem Cell Rep. *20*, 102632.

Medina-Cano, D., Corrigan, E.K., Glenn, R.A., Islam, M.T., Lin, Y., Kim, J., Cho, H., and Vierbuchen, T. (2022). Rapid and robust directed differentiation of mouse epiblast stem cells into definitive endoderm and forebrain organoids. Development *149*, dev200561.

Medina-Cano, D., Islam, M.T., Petrova, V., Dixit, S., Balic, Z., Yang, M.G., Stadtfeld, M., Wong, E.S., and Vierbuchen, T. (2025). A mouse organoid platform for modeling cerebral cortex development and cis-regulatory evolution *in vitro*. Dev. Cell *60*, 3544–3560.e8.

Mòdol, L., Sousa, V.H., Malvache, A., Tressard, T., Baude, A., and Cossart, R. (2017). Spatial Embryonic Origin Delineates GABAergic Hub Neurons Driving Network Dynamics in the Developing Entorhinal Cortex. Cereb. Cortex *27*, 4649–4661.

Mostajo-Radji, M.A., Leon, W.R.M., Breevoort, A., Gonzalez-Ferrer, J., Schweiger, H.E., Lehrer, J., Zhou, L., Schmitz, M.T., Perez, Y., Mukhtar, T., et al. (2025). Fate Plasticity of Interneuron Specification. iScience *28*, 112295.

Okun, M., Yger, P., Marguet, S.L., Gerard-Mercier, F., Benucci, A., Katzner, S., Busse, L., Carandini, M., and Harris, K.D. (2012). Population Rate Dynamics and Multineuron Firing Patterns in Sensory Cortex. J. Neurosci. *32*, 17108–17119.

Okun, M., Steinmetz, N., Cossell, L., Iacaruso, M.F., Ko, H., Barthó, P., Moore, T., Hofer, S.B., Mrsic-Flogel, T.D., Carandini, M., and Harris, K.D. (2015). Diverse Coupling of Neurons to Populations in Sensory Cortex. Nature *521*, 511–515.

Osaki, T., Duenki, T., Chow, S.Y.A., Ikegami, Y., Beaubois, R., Levi, T., Nakagawa-Tamagawa, N., Hirano, Y., and Ikeuchi, Y. (2024). Complex activity and short-term plasticity of human cerebral organoids reciprocally connected with axons. Nat. Commun. *15*, 2945.

Park, Y., Hernandez, S., Hernandez, C.O., Schweiger, H.E., Li, H., Voitiuk, K., Dechiraju, H., Hawthorne, N., Muzzy, E.M., Selberg, J.A., et al. (2024). Modulation of neuronal activity in cortical organoids with bioelectronic delivery of ions and neurotransmitters. Cell Rep. Methods *4*, 100686.

Samarasinghe, R.A., Miranda, O.A., Buth, J.E., Mitchell, S., Ferando, I., Watanabe, M., Allison, T.F., Kurdian, A., Fotion, N.N., Gandal, M.J., et al. (2021). Identification of neural oscillations and epileptiform changes in human brain organoids. Nat. Neurosci. *24*, 1488–1500.

Sánchez, D.J.L., Lindhout, F.W., Anderson, A.J., Pellegrini, L., and Lancaster, M.A. (2024). Mouse Brain Organoids Model *In Vivo* Neurodevelopment and Function and Capture Differences to Human. Preprint at bioRxiv. https://doi.org/10.1101/2024.12.21.629881.

Schroeter, M.S., Charlesworth, P., Kitzbichler, M.G., Paulsen, O., and Bullmore, E.T. (2015). Emergence of rich-club topology and coordinated dynamics in development of hippocampal functional networks *in vitro*. J. Neurosci. *35*, 5459–5470.

Schuurman, T., and Bruner, E. (2024). Modularity and community detection in human brain morphology. Anat. Rec. *307*, 345–355.

Sharf, T., van der Molen, T., Glasauer, S.M.K., Guzman, E., Buccino, A.P., Luna, G., Cheng, Z., Audouard, M., Ranasinghe, K.G., Kudo, K., et al. (2022). Functional Neuronal Circuitry and Oscillatory Dynamics in Human Brain Organoids. Nat. Commun. *13*, 4403.

Tanveer, M.S., Patel, D., Schweiger, H.E., Abu-Bonsrah, K.D., Watmuff, B., Azadi, A., Pryshchep, S., Narayanan, K., Puleo, C., Natarajan, K., et al. (2025). Starting a Synthetic Biological Intelligence Lab from Scratch. Patterns *6*, 101232.

Trujillo, C.A., Gao, R., Negraes, P.D., Gu, J., Buchanan, J., Preissl, S., Wang, A., Wu, W., Haddad, G.G., Chaim, I.A., et al. (2019). Complex Oscillatory Waves Emerging from Cortical Organoids Model Early Human Brain Network Development. Cell Stem Cell *25*, 558–569.e7.

van der Molen, T., Spaeth, A., Chini, M., Hernandez, S., Kaurala, G.A., Schweiger, H.E., Duncan, C., McKenna, S., Geng, J., Lim, M., et al. (2026). Preconfigured neuronal firing sequences in human brain organoids. Nat. Neurosci. *29*, 123–135.

Velasco, S., Kedaigle, A.J., Simmons, S.K., Nash, A., Rocha, M., Quadrato, G., Paulsen, B., Nguyen, L., Adiconis, X., Regev, A., et al. (2019). Individual Brain Organoids Reproducibly Form Cell Diversity of the Human Cerebral Cortex. Nature *570*, 523–527.

Walsh, R.M., Crabtree, G.W., Kalpana, K., Jubierre, L., Koo, S.Y., Ciceri, G., Gogos, J.A., Kruglikov, I., and Studer, L. (2025). Forebrain assembloids support the development of fast-spiking human PVALB$^+$ cortical interneurons and uncover schizophrenia-associated defects. Neuron *113*, 3185–3203.e7.

Watanabe, K., Kamiya, D., Nishiyama, A., Katayama, T., Nozaki, S., Kawasaki, H., Watanabe, Y., Mizuseki, K., and Sasai, Y. (2005). Directed Differentiation of Telencephalic Precursors from Embryonic Stem Cells. Nat. Neurosci. *8*, 288–296.

Watts, D.J., and Strogatz, S.H. (1998). Collective dynamics of 'small-world'networks. Nature *393*, 440–442.

Wu, M.W., Kourdougli, N., and Portera-Cailliau, C. (2024). Network state transitions during cortical development. Nat. Rev. Neurosci. *25*, 535–552.

Stem Cell Reports, Volume *21*

Supplemental Information

Establishing mouse forebrain organoids as models of intrinsic cortical network assembly

Sebastian Hernandez, Hunter E. Schweiger, Isabel Cline, Gregory A. Kaurala, Ash Robbins, Daniel Solis, Samira Vera-Choqqueccota, Jinghui Geng, Tjitse van der Molen, Francisco Reyes, Chinweike Norman Asogwa, Kateryna Voitiuk, Mattia Chini, Marco Rolandi, Sofie R. Salama, Bradley M. Colquitt, Tal Sharf, David Haussler, Mircea Teodorescu, and Mohammed A. Mostajo-Radji

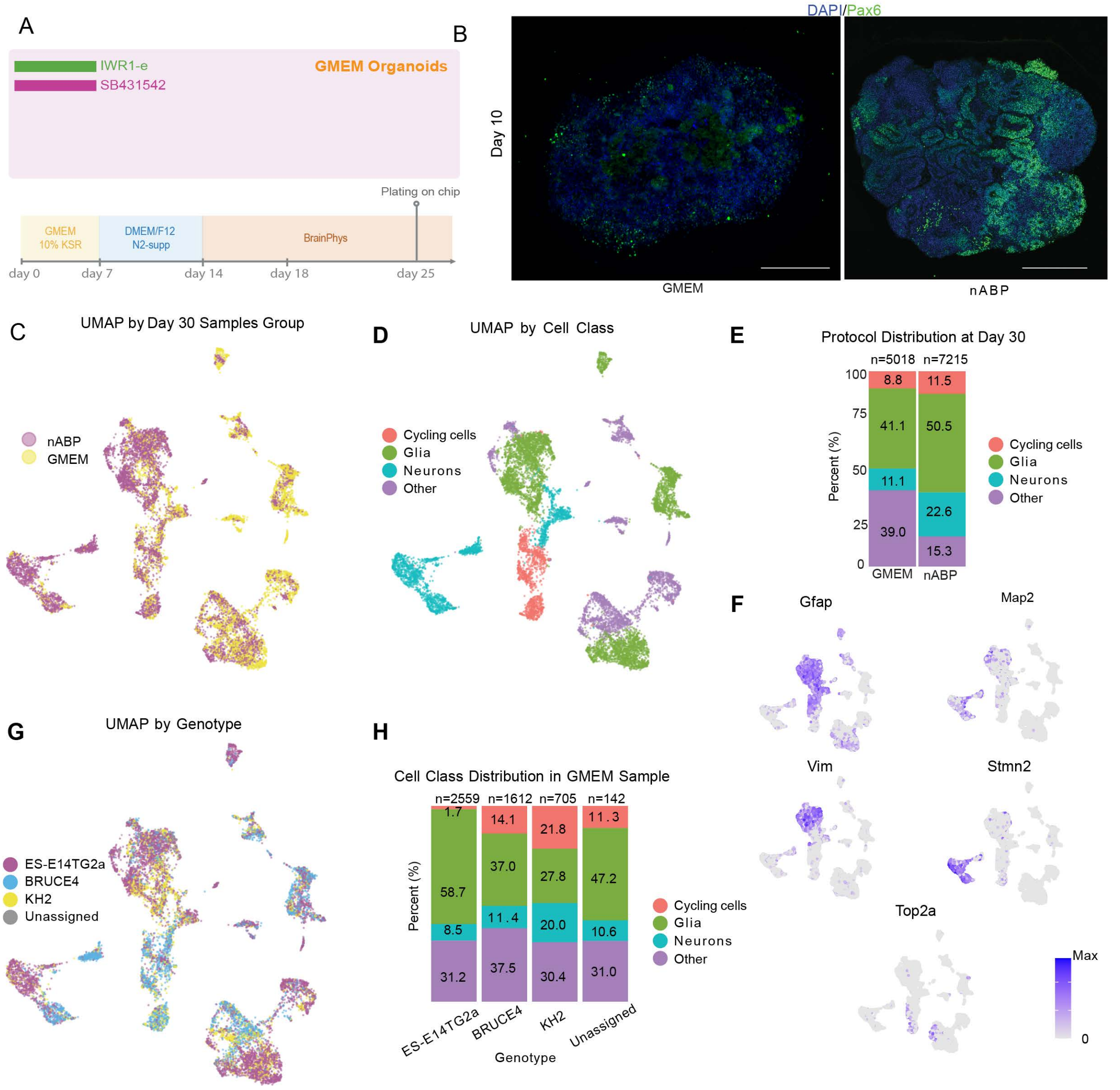

Figure S1: Comparison of GMEM-based and Neurobasal-A/BrainPhys (NABP) protocols in DF organoid development, related to Figure 1.

(A) Schematic of the GMEM-based protocol.

(B) Representative IHC images of day 10 organoids generated using the GMEM-based (left) and nABP (right) protocols, stained for DAPI (blue) and Pax6 (green). Scale bars: 250 µm.

(C) UMAP visualization of single-cell RNA sequencing data from day 30 samples, colored by protocol (NABP and GMEM-based).

(D) UMAP visualization showing cell class distribution (Cycling cells, Glia, Neurons, and Other).

(E) Stacked bar plot comparing cell class distributions between GMEM-based (n = 5,018 cells) and nABP (n = 7,215 cells) protocols at day 30. The NABP protocol shows an increase in neuronal populations and a decrease in off-target "Other" cells.

(F) UMAP plots displaying expression of key marker genes (Gfap, Map2, Vim, Stmn2, and Top2a).

(G) UMAP visualization colored by genotype (ES-E14TG2a, BRUCE4, KH2, and Unassigned).

(H) Detailed cell class distribution across different genotypes in GMEM-based samples. Other indicates off-target cells.

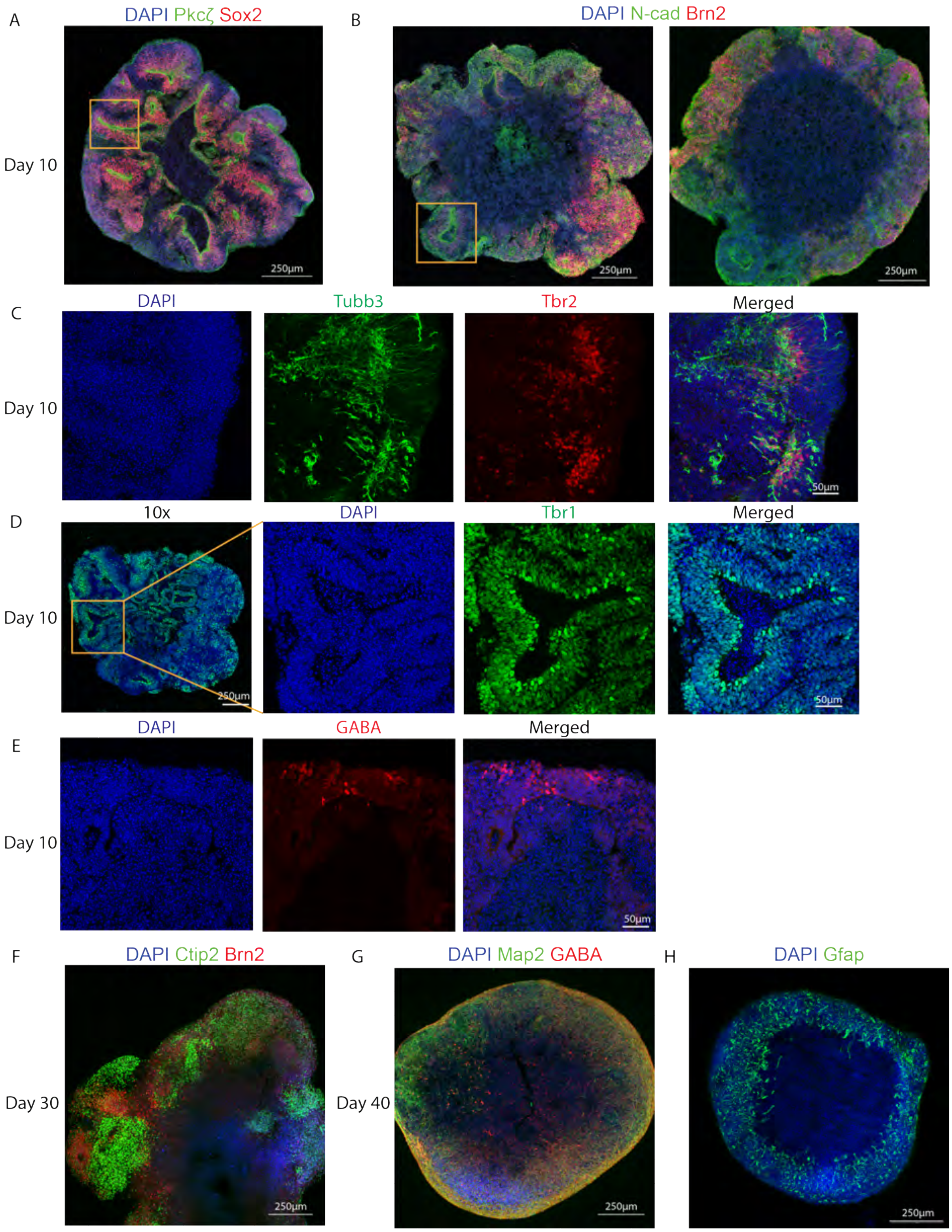
A
DAPI Pkcζ Sox2
Day 10
250µm
B
DAPI N-cad Brn2
250µm
250µm
C
DAPI
Tubb3
Tbr2
Merged
Day 10
50µm
D
10x
DAPI
Tbr1
Merged
Day 10
250µm
50µm
E
DAPI
GABA
Merged
Day 10
50µm
F
DAPI Ctip2 Brn2
Day 30
250µm
G
DAPI Map2 GABA
Day 40
250µm
H
DAPI Gfap
250µm

Figure S2: Immunohistochemical characterization of DF organoids, related to Figure 1.

(A) Low-magnification image of a day 10 organoid stained for Pkcζ (green) and Sox2 (red) (related to figure 1C).
(B) Low-magnification image of a day 10 organoid stained for N-cadherin (green) and Brn2 (red) (related to figure 1C).
(C) IHC of a day 10 DF organoid stained for Tubb3 (green) and Tbr2 (red), with merged image.
(D) IHC of a day 10 organoid. (left) low-magnification overview. (right) high-magnification view of Tbr1 (green).
(E) IHC of a day 10 organoid stained for GABA (red).
(F) Low-magnification image of a day 30 organoid stained for Ctip2 (green) and Brn2 (red) (corresponding to Figure 1D).
(G) IHC of a day 40 organoid stained for Map2 (green) and GABA (red).
(H) IHC of a day 40 organoid stained for Gfap (green).
Scale bars as indicated (50 or 250 μm). DAPI nuclear counterstain shown in blue.

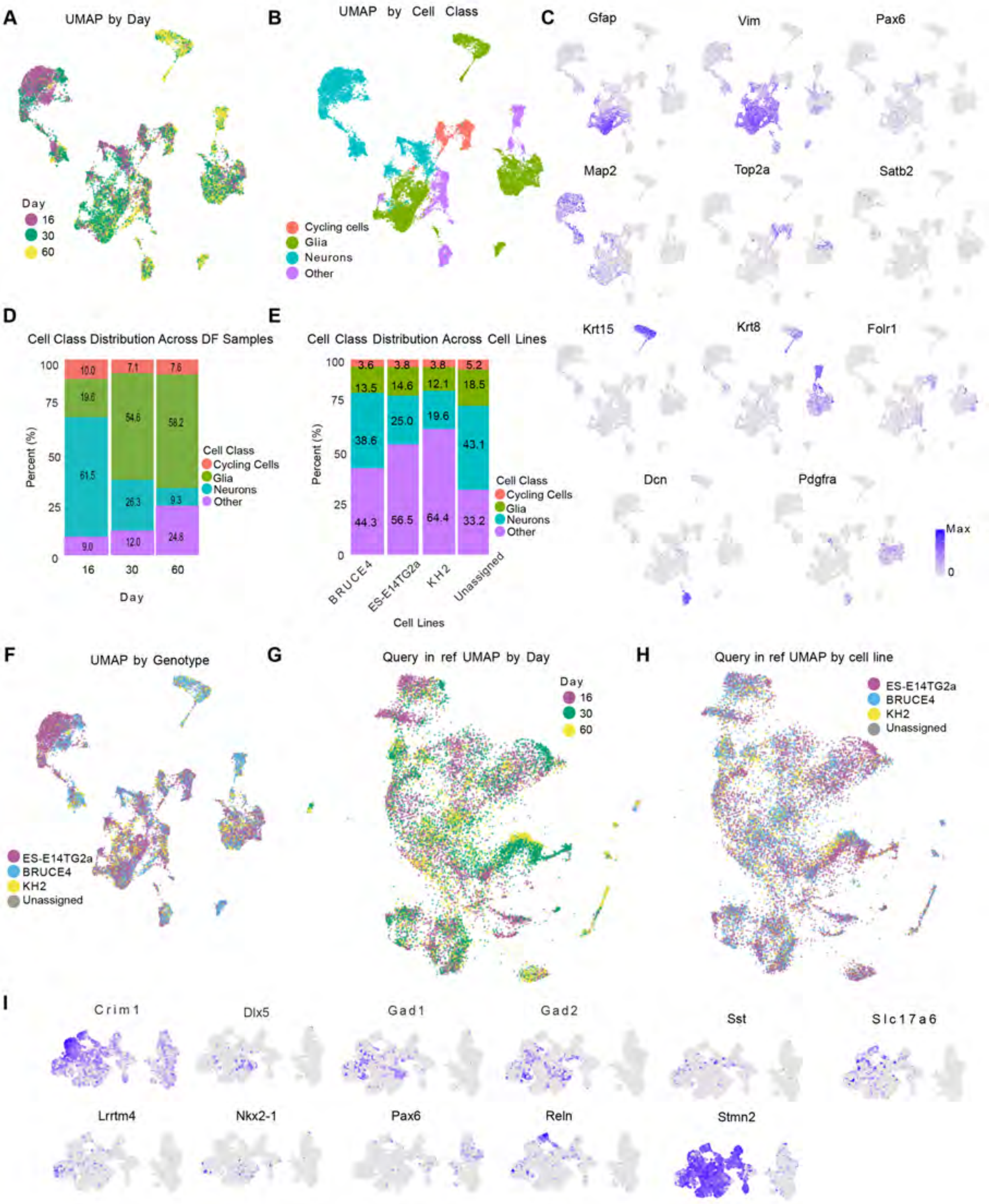

A
UMAP by Day
Day
16
30
60
B
UMAP by Cell Class
Cycling cells
Glia
Neurons
Other
C
Gfap
Vim
Pax6
Map2
Top2a
Satb2
Krt15
Krt8
Folr1
Dcn
Pdgfra
Max
0
D
Cell Class Distribution Across DF Samples
Percent (%)
Day
Cell Class
Cycling Cells
Glia
Neurons
Other
E
Cell Class Distribution Across Cell Lines
BRUCE4
ES-E14TG2a
KH2
Unassigned
Cell Lines
F
UMAP by Genotype
ES-E14TG2a
BRUCE4
KH2
Unassigned
G
Query in ref UMAP by Day
H
Query in ref UMAP by cell line
I
Crim1
Dlx5
Gad1
Gad2
Sst
Slc17a6
Lrrtm4
Nkx2-1
Pax6
Reln
Stmn2

Figure S3: Developmental progression and cellular composition of NABP protocol DF organoids, related to Figure 1.
(A) UMAP by time point visualization of dorsal forebrain cells (days 16, 30, and 60). n = 17,148 cells.
(B) UMAP highlighting major cell classes (Cycling cells, Glia, Neurons, and Other). Other denotes off-target cell types.
(C) Feature plot displaying expression levels of canonical marker genes used for cell type classification (Gfap, Vim, Pax6, Map2, Top2a, Satb2, Krt15, Krt8, Folr1, Dcn, and Pdgfra). Expression intensity is indicated by a color gradient from gray (low) to purple (high) in same UMAP space as A-B.
(D) Stacked bar plot showing the proportional distribution of cell classes at each time-point. Other indicates off-target cells.
(E) Stacked bar plot showing the proportional distribution of cell classes for cell line. Other indicates off-target cells.
(F) UMAP visualization of cells grouped by genotype (ES-E14TG2a, BRUCE4, KH2, and Unassigned).
(G) DF organoid cells mapped into primary reference space colored by timepoint.
(H) Same as G but colored by genotype.
(I) Feature plot displaying expression levels of canonical marker genes used for cell type classification (Crim1, Dlx5, Gad1, Gad2, Sst, Slc17a6, Lrrtm4, Nkx2-1, Pax6, Reln, Stmn2). Expression intensity is indicated by a color gradient from gray (low) to purple (high) in same UMAP space as Figure 1H.

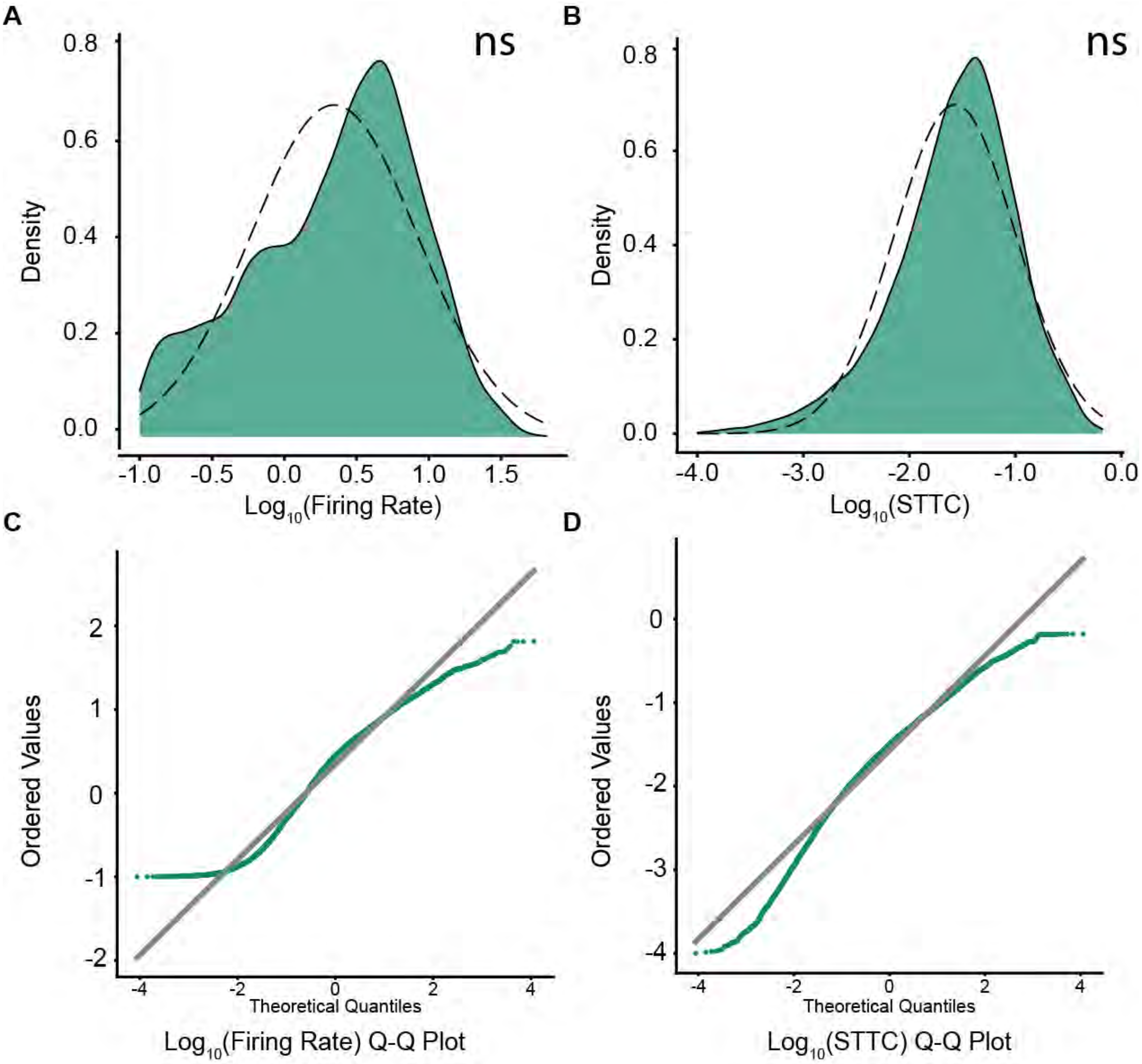

Figure S4: DF and VF organoids exhibit log-normal distributions, related to Figure 2. (A-B) Log-normal distribution of log transformed mean firing rate distribution (A) and log transformed mean STTC (B) (green) with theoretical normal distribution (dashed line). (C-D) Q-Q Plots showing that both log transformed mean firing rate distribution (A) and log transformed mean STTC follow a log-normal distribution.
ns = not significant. Kolmogorov–Smirnov test

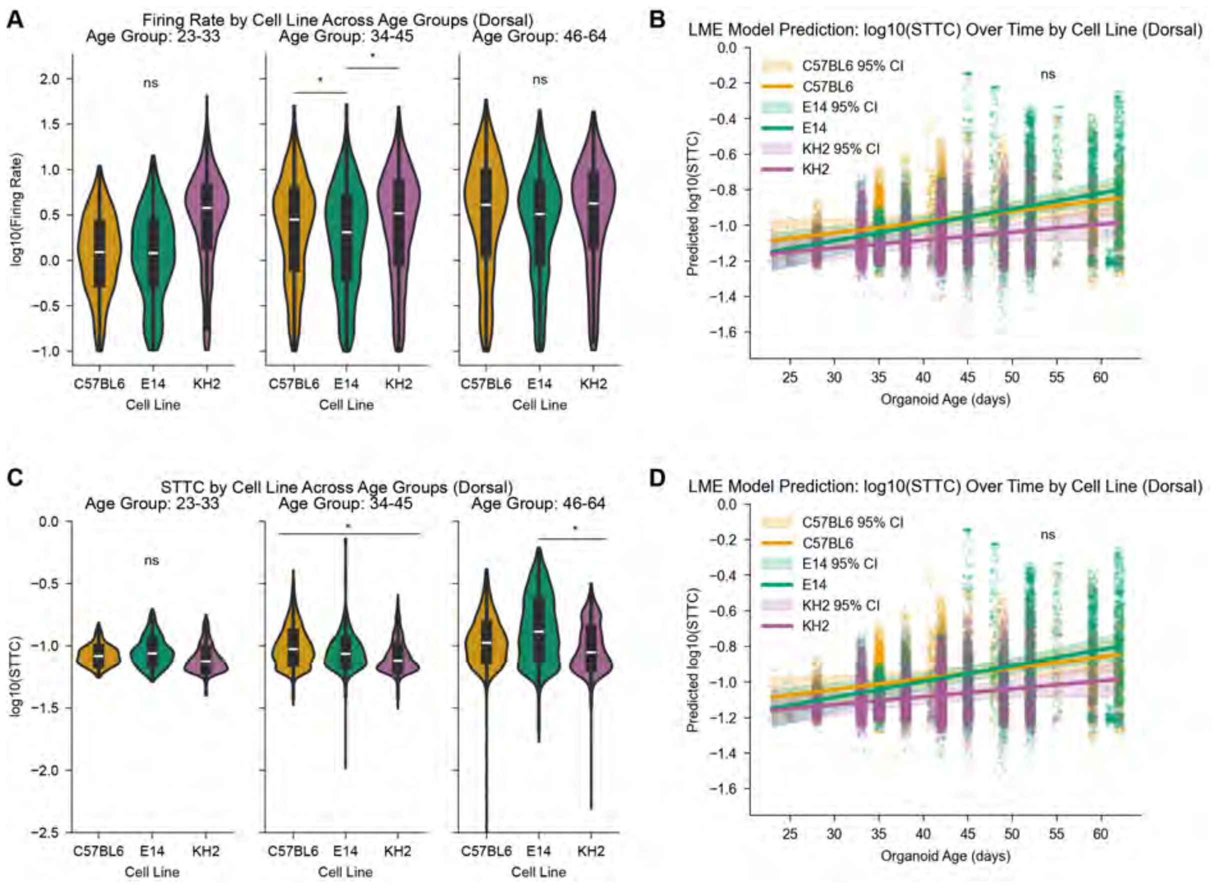

Figure S5: Similar DF electrophysiological distributions across cell lines, related to Figure 2.

(A) Violin plots showing log10(Firing Rate) distributions by cell line (C57BL6, ES-E14TG2a, and KH2) across three age groups (23-33, 34-45, and 46-64) in DF organoids. n = 16 organoids, 28,809 units.

(B) LME model predictions of log10(STTC) over development by cell line for DF organoids. (C) Violin plots showing log10(STTC) distributions by cell line across the same three age groups in DF organoids. (D) LME model prediction of log10(STTC) over development by cell line for DF organoids, similar to panel B but with potentially different parameter settings.

ns = not significant, $p < 0.017$ (Bonferroni corrected). Mixed-effects model

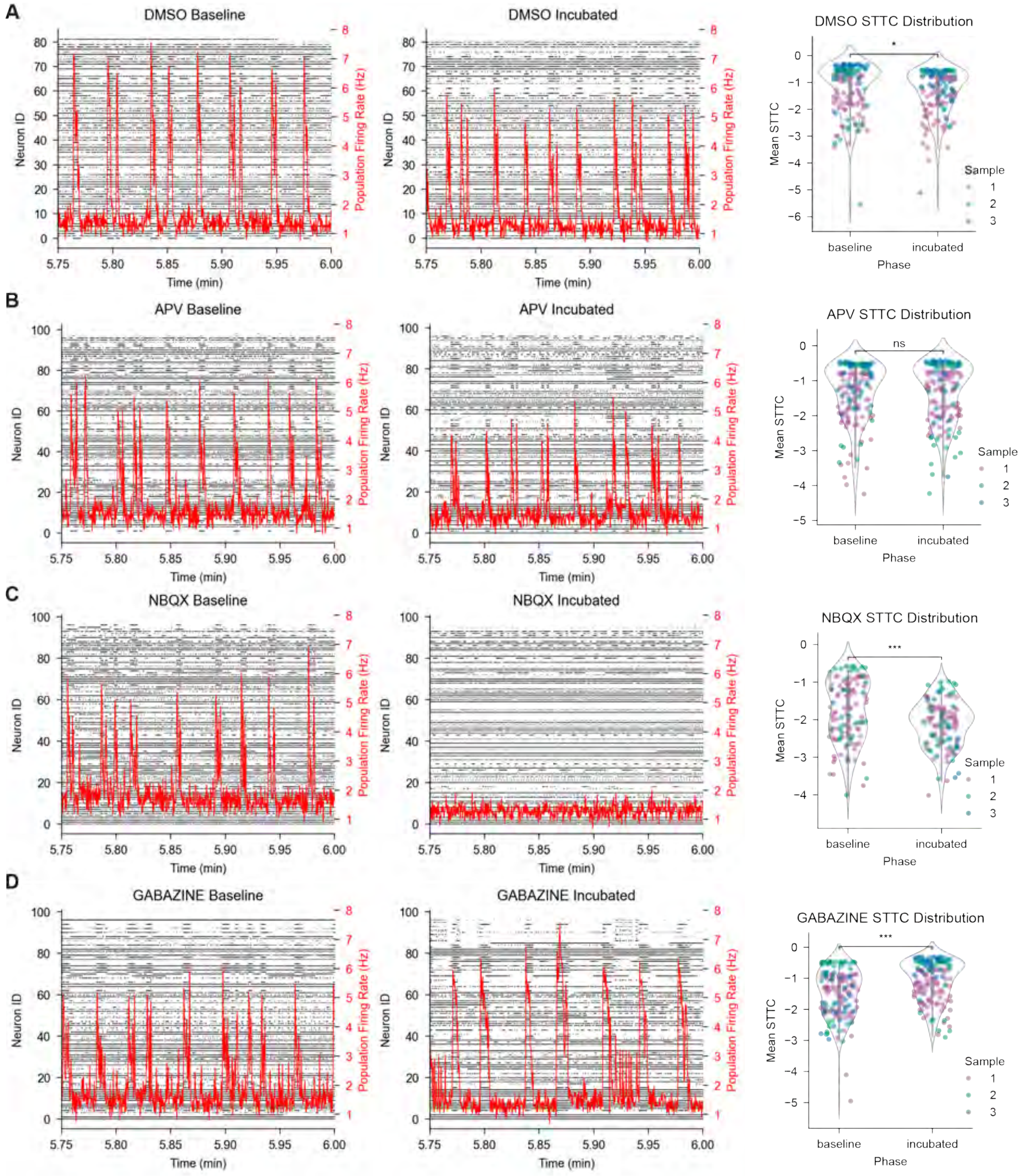

Figure S6: Impact of pharmacological perturbation on overall neural connectivity, related to Figure 2.

(A) Raster plots of neural activity (gray) and population firing rate (red) during baseline (left) and post-drug incubation (middle) for a 15s window in DMSO vehicle control. Each row represents individual unit spike trains. (Right) STTC distribution showing the mean log STTC values across all 3 organoid samples. Organoid 1 is shown in purple (n = 97), organoid 2 (n = 53) in green, and organoid 3 (n = 35) in yellow. Statistical significance indicated by asterisks: * $p < 0.05$, ** $p < 0.001$, *** $p < 0.0001$, ns: not significant.

(B) As in panel A but following treatment with NMDA antagonist APV.

(C) As in panel A but following treatment with AMPA/Kainate antagonist NBQX.

(D) As in panel A but following treatment with $GABA_A$ antagonist Gabazine.

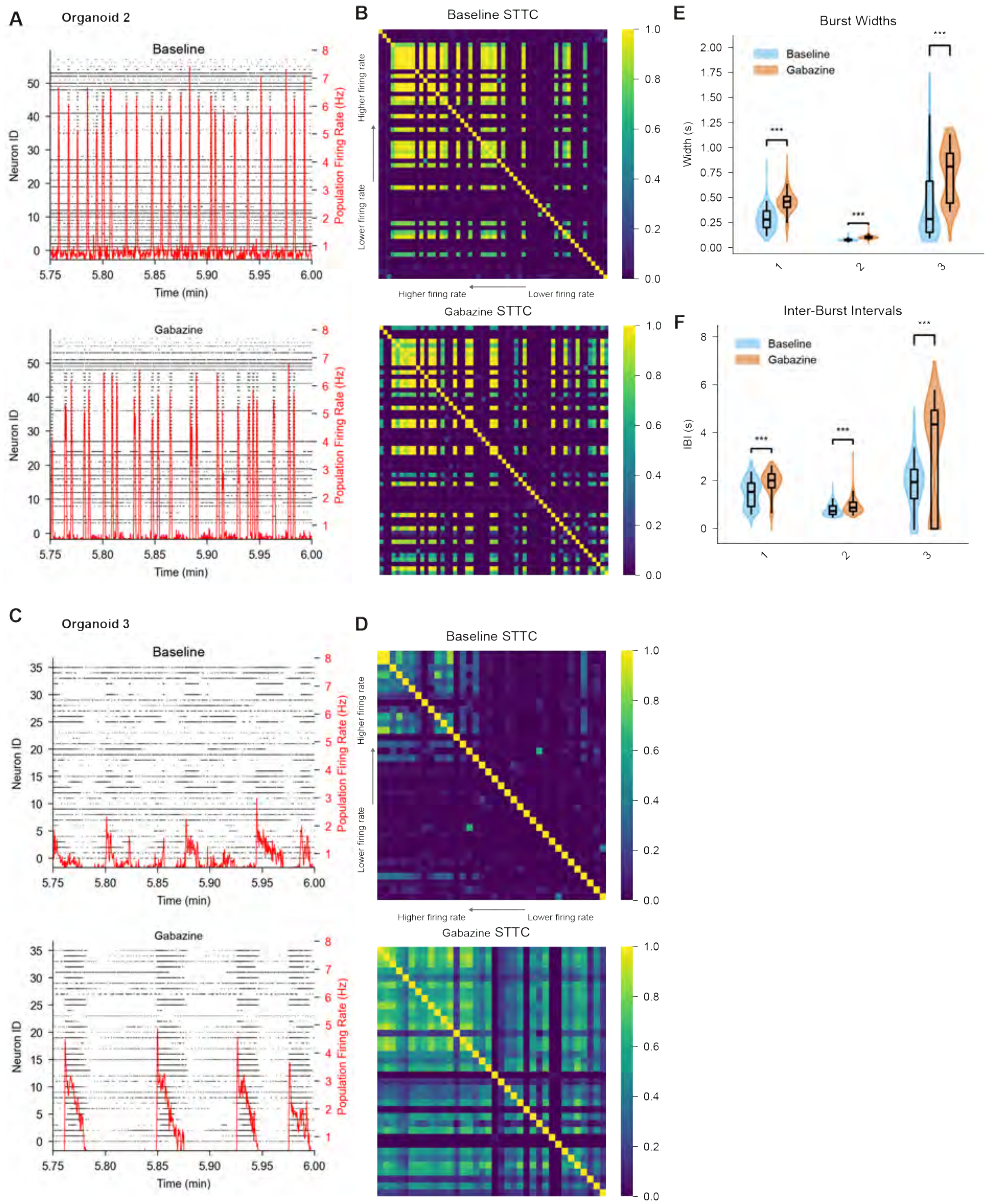

Figure S7: Effects of GABA receptor antagonism on bursting activity in dorsal forebrain organoids, related to Figure 2.

(A) Representative raster plots from Sample 2 showing neural activity (gray) and population firing rate (red) during baseline (left) and post-Gabazine incubation (middle) over a 15s window. Each row represents a single-unit spike train. n = 53

(B) STTC matrices sorted by firing rate (ascending to descending). (left) Baseline STTC matrix. Middle: STTC matrix post-Gabazine incubation. (right) Difference matrix showing STTC changes. n = 53

(C) Same as (A), but for Sample 3. n = 35

(D) Same as (B), but for Sample 3. n = 35

(E) Burst width across three organoids.

(F) Inter-burst interval across three organoids.

(E–F) Statistical comparison of baseline vs. Gabazine conditions. Statistical significance: * p < 0.05, ** p < 0.001, *** p < 0.0001; ns = not significant. Mann–Whitney U test.

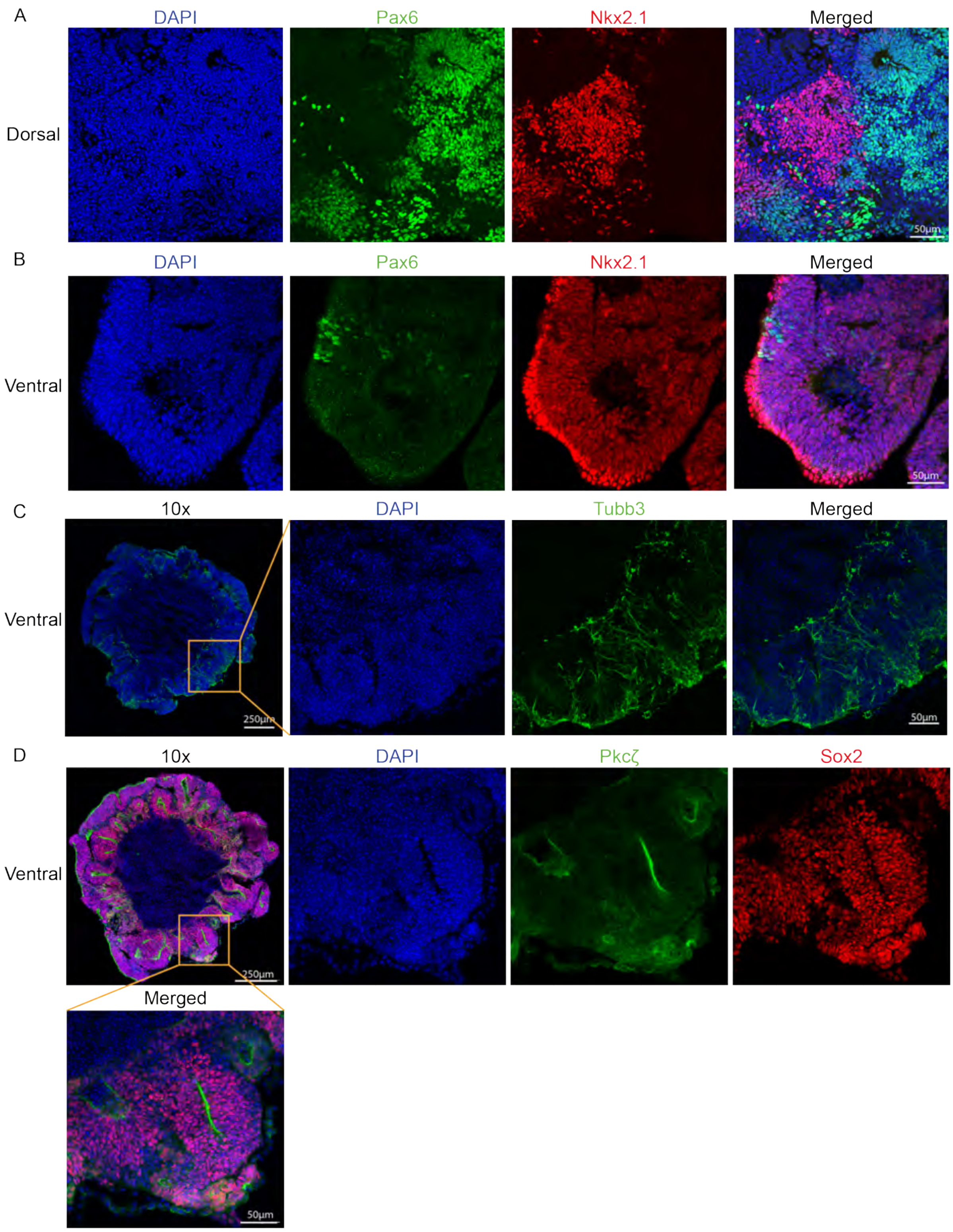

Figure S8: Patterning marker expression in developing DF and VF organoids, related to Figure 3.
(A) High-magnification view of the DF from Figure 3D showing Pax6 (green) and Nkx2.1 (red) staining with the merged image.
(B) High-magnification view of the VF from Figure 3D, showing Pax6 (green) and Nkx2.1 (red) staining with the merged image.
(C) Day 10 VF organoid. (left) Low-magnification overview. (right) High-magnification view of Tubb3 (green) staining with the merged image.
(D) Day 10 VF organoid. (left) Low-magnification overview. (right) High-magnification view of Pkcζ (green) and Sox2 (red).
All panels include DAPI nuclear counterstain (blue), with scale bars as indicated (50 or 250 μm).

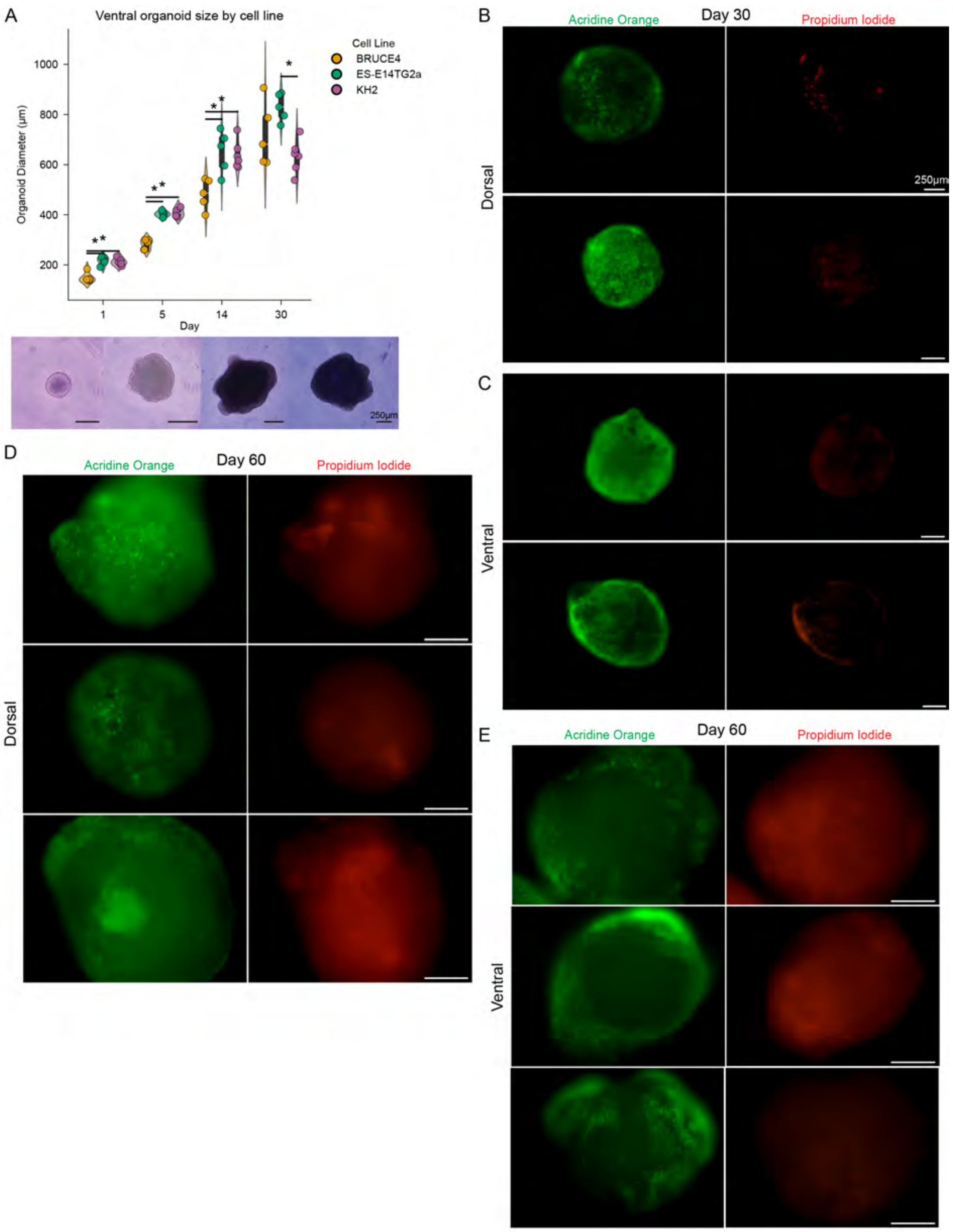

A
Ventral organoid size by cell line
Organoid Diameter (µm)
Day
Cell Line
BRUCE4
ES-E14TG2a
KH2
250µm
B
Acridine Orange
Day 30
Propidium Iodide
Dorsal
C
Ventral
D
Day 60
E

Figure S9: DF and VF organoids maintain viability and similar size over long term development, related to Figure 3.
(A) VF organoid size over time split by cell lines. n = 5 organoids per cell line
(B) Live/dead viability dye in day 30 DF organoids showing acridine orange (green) and propidium iodide (red).
(C) Same as B for day 30 VF organoids
(D) Same as B for day 60 DF organoids
(E) Same as B for day 60 VF organoids
Dunn's post hoc test, * $p < 0.05$.

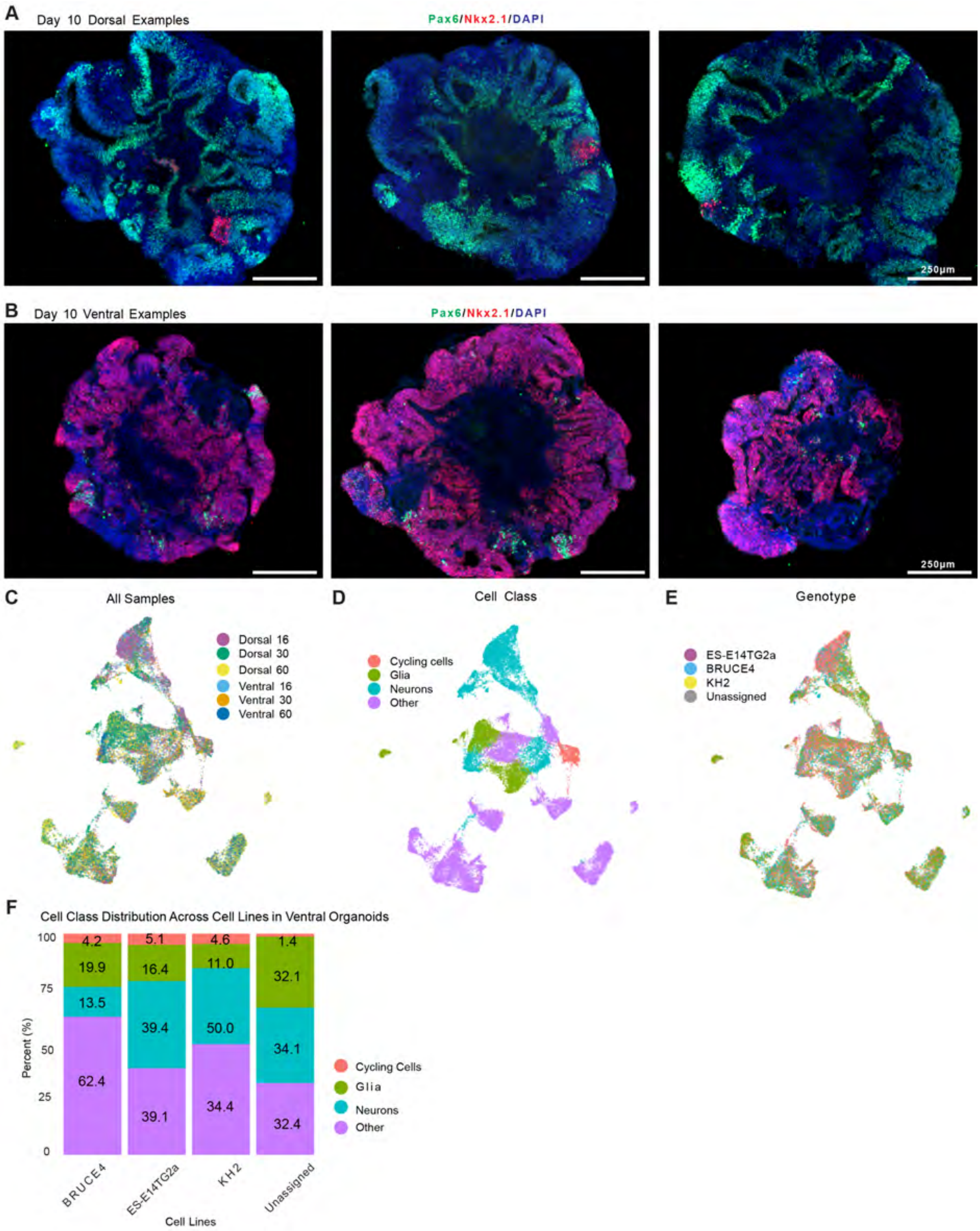
A
Day 10 Dorsal Examples
Pax6/Nkx2.1/DAPI
250μm
B
Day 10 Ventral Examples
Pax6/Nkx2.1/DAPI
250μm
C
All Samples
Dorsal 16
Dorsal 30
Dorsal 60
Ventral 16
Ventral 30
Ventral 60
D
Cell Class
Cycling cells
Glia
Neurons
Other
E
Genotype
ES-E14TG2a
BRUCE4
KH2
Unassigned
F
Cell Class Distribution Across Cell Lines in Ventral Organoids
Percent (%)
100
75
50
25
0
4.2
19.9
13.5
62.4
5.1
16.4
39.4
39.1
4.6
11.0
50.0
34.4
1.4
32.1
34.1
32.4
BRUCE4
ES-E14TG2a
KH2
Unassigned
Cell Lines
Cycling Cells
Glia
Neurons
Other

Figure S10: Characterization of DF and VF organoid development, related to Figure 3.

(A) IHC images of additional day 10 DF organoids stained for Pax6 (green) and Nkx2.1 (red). These organoids were used for quantifications in Figure 3D.
(B) IHC images of additional day 10 VF organoids stained for Pax6 (green) and Nkx2.1 (red). These organoids were used for quantifications in Figure 3D.
(C) UMAP visualization of scRNA-seq data colored by sample type (DF and VF). n = 10,495 cells.
(D) UMAP plot showing cell class (Cycling cells, Glia, Neurons, and Other). Other indicates off-target cells.
(E) UMAP visualization colored by genotype (ES-E14TG2a, BRUCE4, KH2, and Unassigned).
(F) Stacked bar plot showing cell class distribution across genotypes in VF samples. Other indicates off-target cells. Panels (A–B) include DAPI nuclear counterstain (blue) and scale bars as indicated (250 μm).

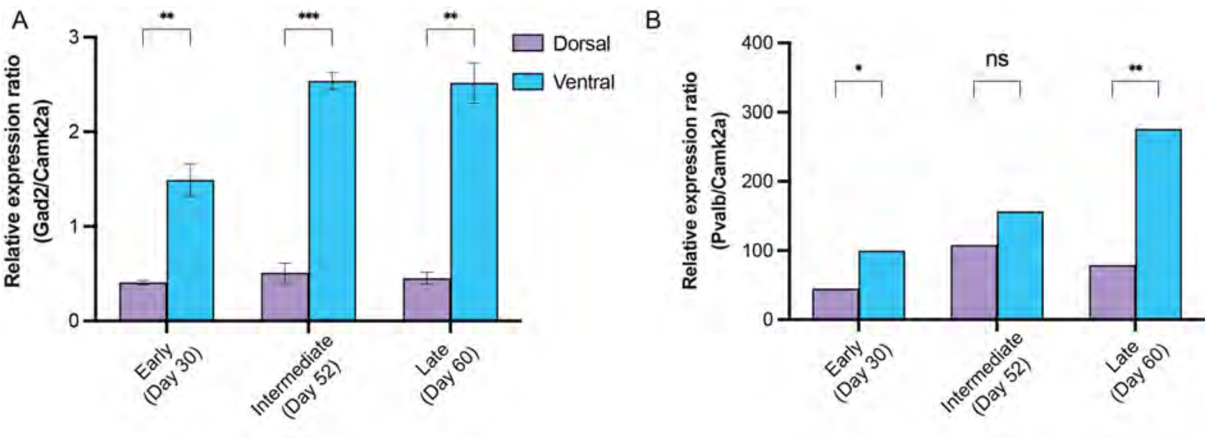

Figure S11: Relative expression of GABAergic and glutamatergic genes, related to Figure 4.

(A) Relative expression ratio of *Gad2* divided by *Camk2a* for timepoints: early (day 30), intermediate (day 52), and late (day 60) n = 3 organoids across 3 separate batches.

(B) Same as (A) but for relative expression ratio of *Pvalb* divided by *Camk2a*.

Core-Periphery, Hubness & development - DF Organoids
(Same STTC chip: through 23124)

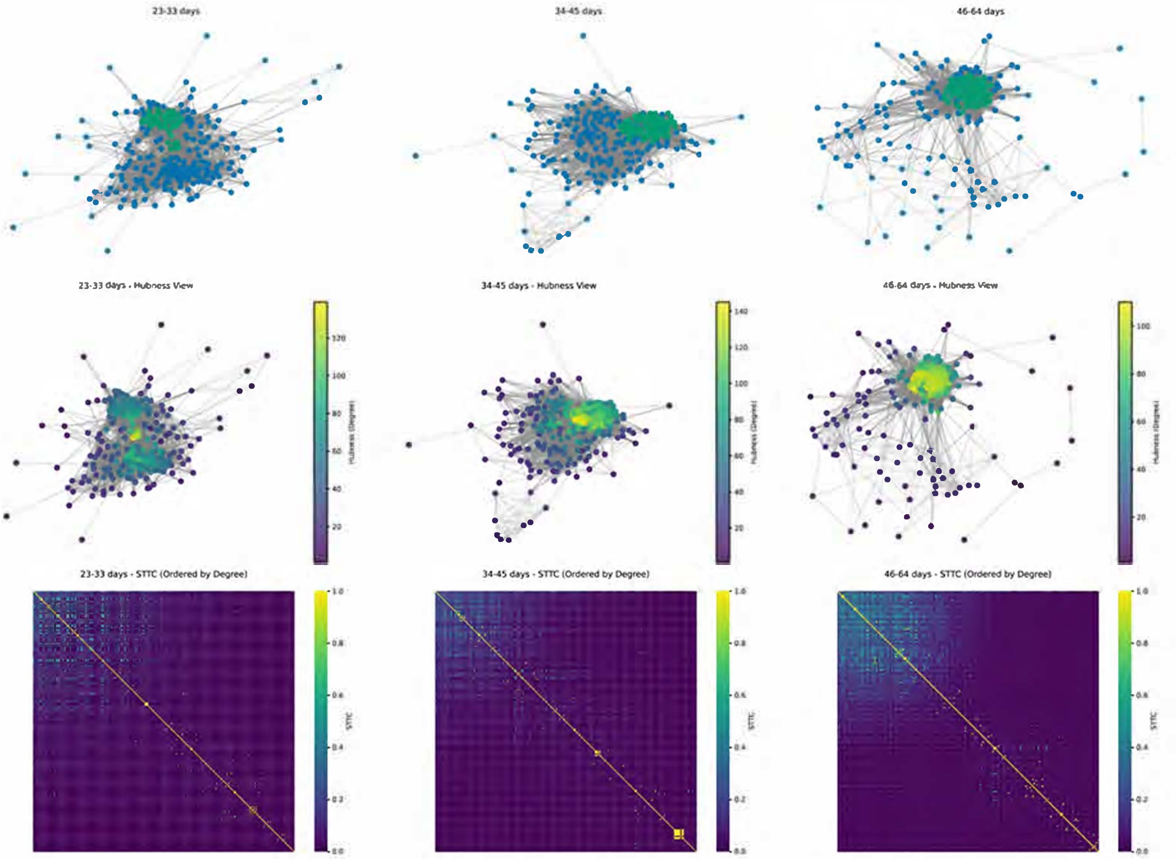

Core-Periphery, Hubness & development - DF Organoids
(Same STTC chip: through 22710)

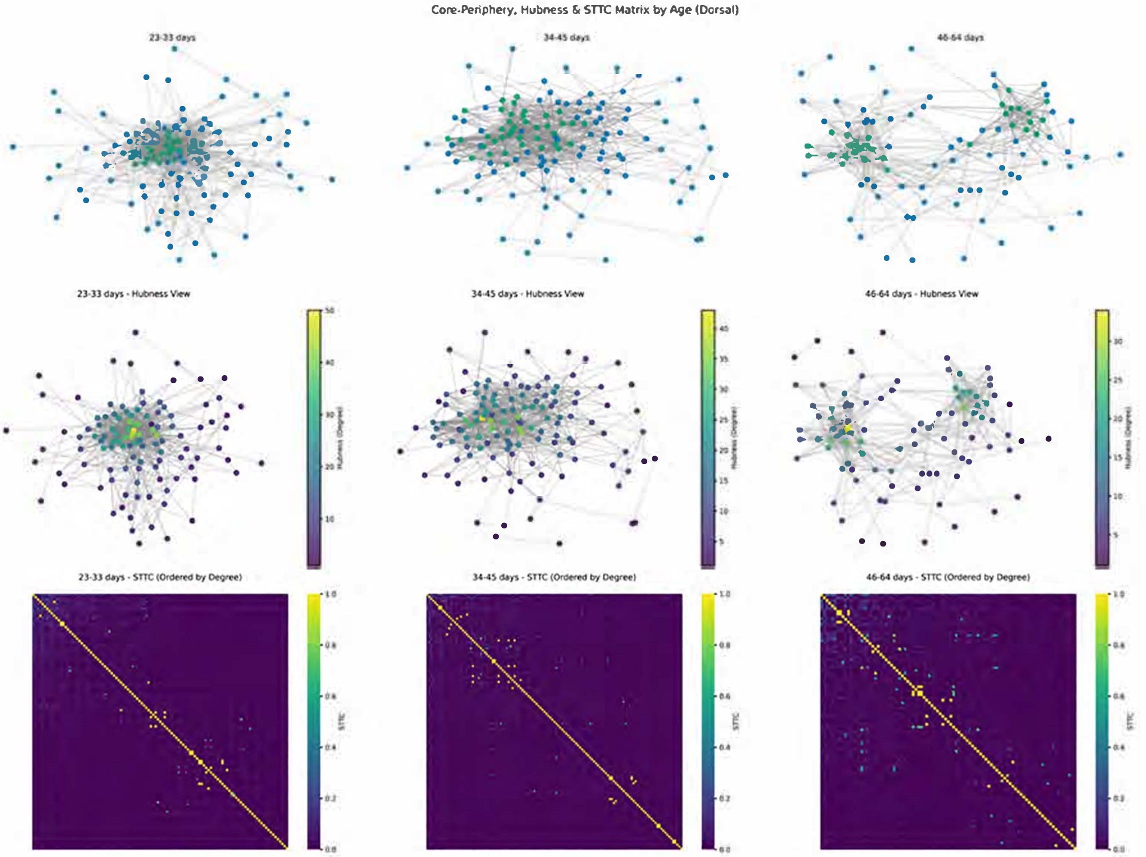

Figure S12: Developmental changes in network connectivity of DF organoids tracked longitudinally, related to Figure 6.
(A) Longitudinal analysis of organoid chip 23120 shown at three developmental timepoints (23-33 days, 34-45 days, and 46-64 days). Upper panels display hubness visualizations where node colors represent hubness score. Lower panels show corresponding STTC matrices ordered by connection degree. n = green: 46, 79, 73; blue: 195, 165, 116. units
(B) Longitudinal analysis of organoid chip 23120 shown at three developmental timepoints (23-33 days, 34-45 days, and 46-64 days). Upper panels display hubness visualizations where node colors represent hubness score. Lower panels show corresponding STTC matrices ordered by connection degree. green: 21, 39, 28; blue: 90, 90, 67.

Core-Periphery, Hubness & development - VF Organoids
(Same STTC chip: through 25136)

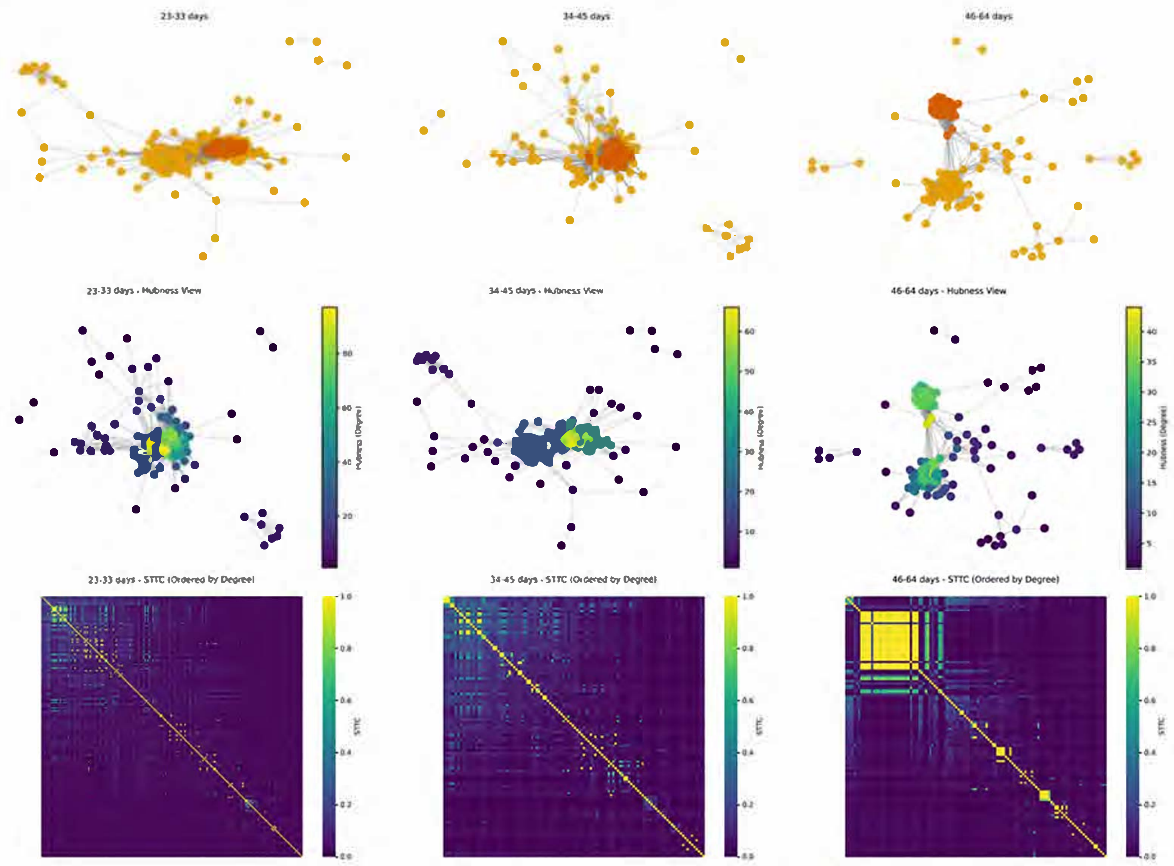

Core-Periphery, Hubness & development - VF Organoids
(Same STTC chip: through 22064b)

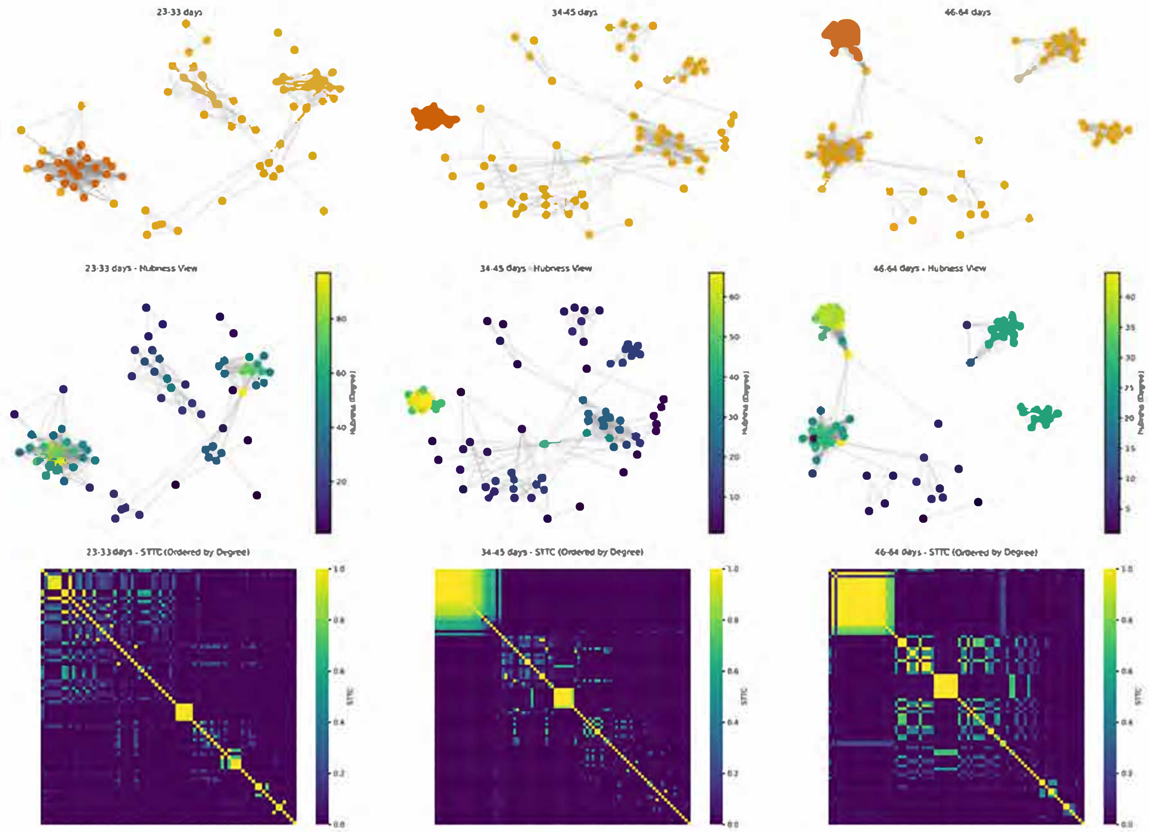

Figure S13: Developmental changes in network connectivity of VF organoids tracked longitudinally, related to Figure 6.
(A) Longitudinal analysis of organoid chip 25136 shown at three developmental time-points (23-33 days, 34-45 days, and 46-64 days). Upper panels display hubness visual-izations where node colors represent hubness score. Lower panels show corresponding STTC matrices ordered by connection degree. n = orange: 58, 39, 35; yellow: 132, 77, 86.
(B) Longitudinal analysis of organoid chip 22064b shown at three developmental time-points (23-33 days, 34-45 days, and 46-64 days). Upper panels display hubness visual-izations where node colors represent hubness score. Lower panels show corresponding STTC matrices ordered by connection degree. n = orange: 21, 22, 22; yellow: 53, 66, 63.

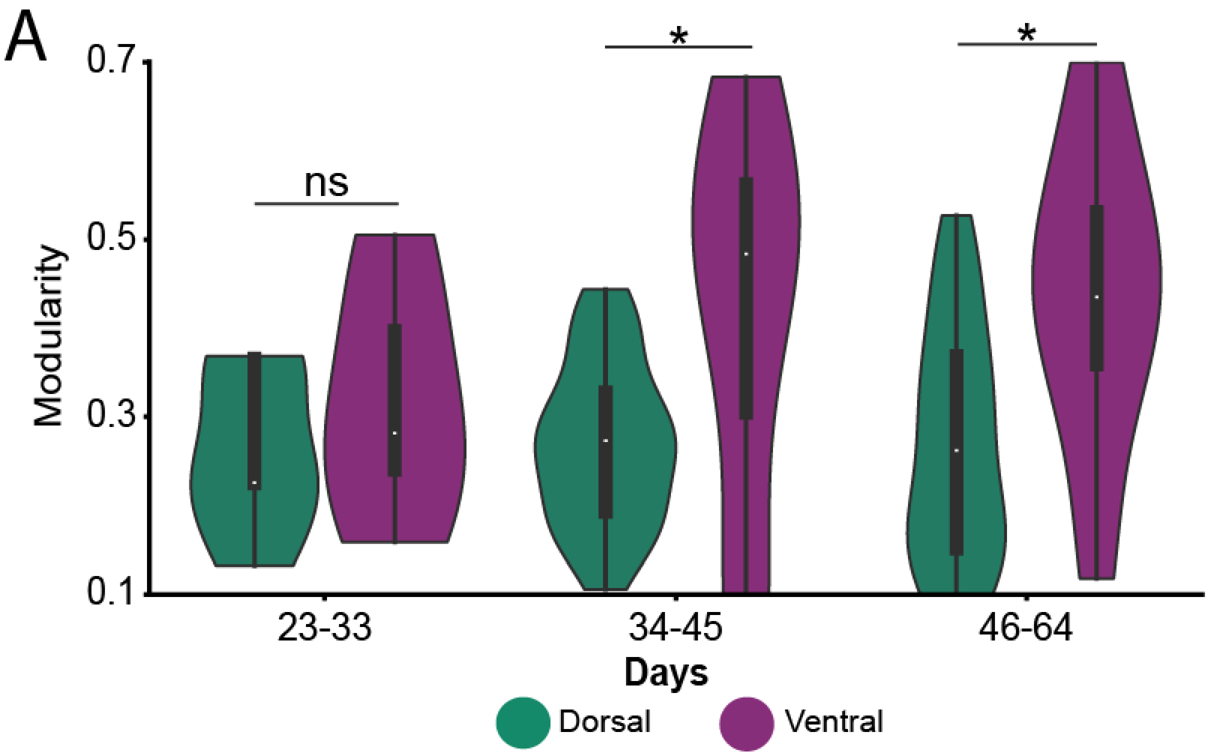

Figure S14: Developmental differences in modularity metric between DF and VF organoids, related to Figure 6.
(A) Violin plots showing network modularity values for DF (green; n = 16) and VF (purple; n = 18) organoids across three developmental time windows (23-33 days, 34-45 days, and 46-64 days). No significant difference in modularity is observed during early develop-ment (23-33 days, ns). However, VF organoids display significantly higher modu-larity compared to DF organoids during both mid (34-45 days) and late (46-64 days) developmental stages (* indicates p<0.05 Mann–Whitney U test).

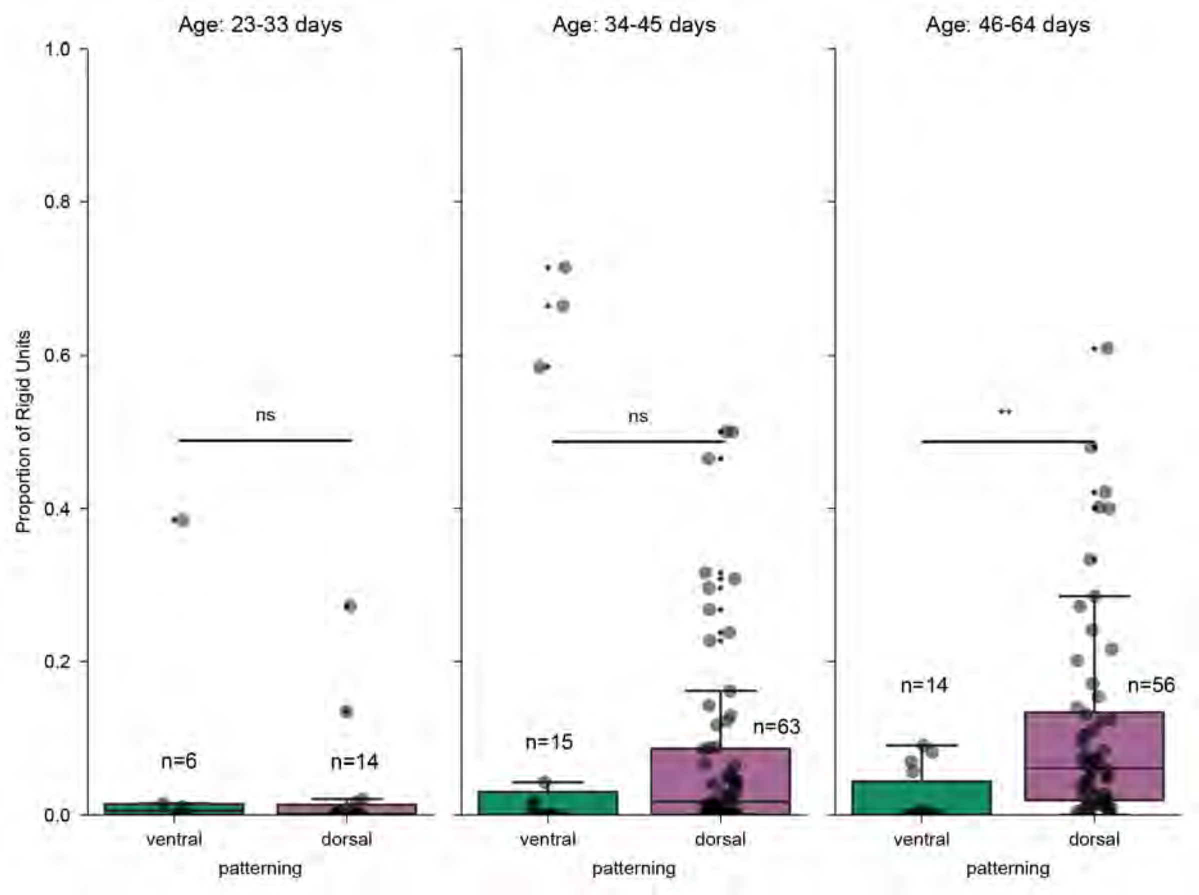

Figure S15: Proportion of rigid units over development for DF and VF, related to Figure 7.
(A) Proportion of rigid units over development for VF (Left; 23-33; n = 5; 34-45; n = 11; 46-64; n = 9)) and DF (Right; 23-33; n = 11; 34-45; n = 11; 46-64; n = 12).
ns = not significant, **p < 0.001, Mann-Whitney U test.

Supplemental Tables

Table S1: Statistical Comparison of Metrics Across Age Groups within Dorsal Organoids, related to Figure 2.
Firing rate (FR), Spike time tiling coefficient (STTC)
Mixed-effects model *p < 0.017, **p < 0.003, ***p < 0.001 (Bonferroni corrected)

Metric	Pairwise Age Group Comparisons				
	Age Groups	Diff	Std.Err.	z-value	p-value
$\log_{10}$ FR	23-33 vs 34-45	-0.202	0.053	-3.825	< 0.001**
	23-33 vs 46-64	-0.271	0.054	-5.063	< 0.001**
	34-45 vs 46-64	-0.069	0.035	-1.954	0.05
$\log_{10}$ STTC	23-33 vs 34-45	-0.087	0.040	-2.202	0.03*
	23-33 vs 46-64	-0.190	0.040	-4.728	< 0.001**
	34-45 vs 46-64	-0.103	0.027	-3.841	< 0.001**

Table S2: Statistical Comparison of Metrics Across Cell Lines within Dorsal Organoids, related to Figure 2.
Firing rate (FR), Spike time tiling coefficient (STTC)
Mixed-effects model *p < 0.017, **p < 0.003, ***p < 0.001 (Bonferroni corrected)

Metric	Age Group	Pairwise Cell Line Comparisons				
		Cell Lines	Diff	Std.Err.	z-value	p-value
$\log_{10}$ FR	23-33	C57BL6 vs E14	0.061	0.184	0.331	0.74
		C57BL6 vs KH2	-0.119	0.165	-0.722	0.47
		E14 vs KH2	-0.180	0.129	-1.399	0.16
	34-45	C57BL6 vs E14	0.144	0.055	2.614	0.009**
		C57BL6 vs KH2	-0.049	0.050	-0.973	0.33
		E14 vs KH2	-0.193	0.056	-3.464	0.001**
	46-64	C57BL6 vs E14	0.123	0.055	2.249	0.03*
		C57BL6 vs KH2	0.007	0.063	0.109	0.91
		E14 vs KH2	-0.116	0.061	-1.907	0.06
$\log_{10}$ STTC	23-33	C57BL6 vs E14	0.015	0.034	0.433	0.67
		C57BL6 vs KH2	0.046	0.031	1.509	0.13
		E14 vs KH2	0.031	0.024	1.315	0.19
	34-45	C57BL6 vs E14	0.019	0.040	0.467	0.64
		C57BL6 vs KH2	0.115	0.036	3.154	0.002**
		E14 vs KH2	0.096	0.041	2.370	0.02*
	46-64	C57BL6 vs E14	-0.040	0.051	-0.783	0.43
		C57BL6 vs KH2	0.106	0.058	1.819	0.07
		E14 vs KH2	0.146	0.057	2.586	0.01**

Table S3: Drug Effects on Firing Rate (FR), related to Figure 2.
Mixed-effects model, * Significant at p < 0.05. SEM = Standard Error of Mean

Drug	Baseline Mean (SEM)	Drug Mean (SEM)	p-value	Significant	Coefficient (SE)
APV	21.94 ± 1.04	18.78 ± 1.02	0.01	Yes*	-0.080 ± 0.108
DMSO	22.03 ± 1.19	19.63 ± 1.23	0.21	No	-0.064 ± 0.052
GABAZINE	20.81 ± 1.24	23.06 ± 1.46	0.79	No	$+0.017 \pm 0.063$
NBQX	16.64 ± 1.19	13.37 ± 1.12	0.05	No	-0.171 ± 0.088

* Significant at p < 0.05

Table S4: Drug Effects on Spike Time Tiling Coefficient (STTC), related to Figure 2.
Mixed-effects model, * Significant at p < 0.05. SEM = Standard Error of Mean

Drug	Baseline Mean (SEM)	Drug Mean (SEM)	p-value	Significant	Coefficient (SE)
APV	0.126 ± 0.010	0.132 ± 0.011	0.46	No	-0.080 ± 0.108
DMSO	0.168 ± 0.014	0.116 ± 0.010	0.52	No	-0.066 ± 0.104
GABAZINE	0.107 ± 0.011	0.188 ± 0.014	< 0.001	Yes*	$+0.352 \pm 0.084$
NBQX	0.063 ± 0.010	0.022 ± 0.004	0.003	Yes*	-0.324 ± 0.110

Table S5: Organoid size (diameter) comparisons across cell lines and patterning conditions over time, related to Figure 3.
Kruskal-Wallis tests with Dunn's post-hoc comparisons. *p < 0.05, **p < 0.01, ***p < 0.001, ns = not significant.

Comparison	Measure	Timepoints			
		Day 1	Day 5	Day 14	Day 30
Dorsal: Cell Line Comparisons					
**Overall KW Test**	p-value	**0.0088****	**0.0090****	**0.0076****	**0.0029****
	BRUCE4 vs E14	**0.0360***	**0.0131***	**0.0235***	0.0607 (ns)
	BRUCE4 vs KH2	**0.0155***	0.0573 (ns)	**0.0158***	**0.0027****
	E14 vs KH2	1.0000 (ns)	1.0000 (ns)	1.0000 (ns)	1.0000 (ns)
Ventral: Cell Line Comparisons					
**Overall KW Test**	p-value	**0.0087****	**0.0110***	**0.0105***	**0.0257***
	BRUCE4 vs E14	**0.0140***	**0.0305***	**0.0194***	0.4318 (ns)
	BRUCE4 vs KH2	**0.0400***	**0.0293***	**0.0340***	0.7148 (ns)
	E14 vs KH2	1.0000 (ns)	1.0000 (ns)	1.0000 (ns)	**0.0205***
Dorsal vs Ventral (pooled across cell lines)					
**t-test**	p-value	0.6737 (ns)	0.8490 (ns)	0.5194 (ns)	0.2925 (ns)
	n (D/V)	15/15	14/14	16/16	17/16

Table S6: Statistical Comparison of FR and STTC Across Age Groups within VF Organoids, related to Figure 4.
Firing rate (FR), Spike time tiling coefficient (STTC)
Mixed-effects model *p < 0.017, **p < 0.003, ***p < 0.001 (Bonferroni corrected)

Metric	Pairwise Age Group Comparisons				
	Age Groups	Diff	Std.Err.	z-value	p-value
$\log_{10}$ FR	23-33 vs 34-45	-0.292	0.090	-3.259	0.001**
	23-33 vs 46-64	-0.271	0.088	-3.089	0.002**
	34-45 vs 46-64	0.021	0.067	0.309	0.76
$\log_{10}$ STTC	23-33 vs 34-45	-0.100	0.048	-2.106	0.04*
	23-33 vs 46-64	-0.056	0.047	-1.193	0.23
	34-45 vs 46-64	0.045	0.036	1.251	0.21

Table S7: Statistical Comparison of FR and STTC Between DF and VF Organoids Across Age Groups, related to Figure 4.
Firing rate (FR), Spike time tiling coefficient (STTC)
Mixed-effects model, * Significant at $p < 0.05$.

Age Group	Metric	Coefficient		Significance		
		Estimate	Std.Err.	z-value	p-value	Significant
23-33 days	FR	-0.077	0.093	-0.828	0.41	No
	STTC	0.032	0.022	1.460	0.14	No
34-45 days	FR	0.011	0.051	0.222	0.82	No
	STTC	0.046	0.036	1.261	0.21	No
46-64 days	FR	-0.078	0.053	-1.459	0.15	No
	STTC	-0.102	0.043	-2.344	0.02*	Yes

Table S8: Age Group Comparisons for Small World Metrics in DF organoids, related to Figure 4.
Mixed-effects model *$p < 0.017$, **$p < 0.003$, ***$p < 0.001$ (Bonferroni corrected)

Metric	Measure	Age Group Comparisons		
		23-33 vs 34-45 days	34-45 vs 46-64 days	23-33 vs 46-64 days
**Small World (S)**	p-value	**< 0.001*****	**< 0.001*****	**< 0.001*****
	Effect Size	0.178	0.0208	0.199
**Clustering (C_norm)**	p-value	**< 0.001*****	**< 0.001*****	**< 0.001*****
	Effect Size	0.319	0.0701	0.389
**Path Length (L_norm)**	p-value	**< 0.001*****	**< 0.001*****	**< 0.001*****
	Effect Size	0.046	0.0101	0.056

Table S9: Age Group Comparisons for Small World Metrics in VF organoids, related to Figure 4.
Mixed-effects model, *$p < 0.017$, **$p < 0.003$, ***$p < 0.001$ (Bonferroni corrected)

Metric	Measure	Age Group Comparisons		
		23-33 vs 34-45 days	34-45 vs 46-64 days	23-33 vs 46-64 days
**Small World (S)**	p-value	**0.003****	**< 0.001*****	**< 0.001*****
	Effect Size	0.162	0.0727	0.235
**Clustering (C_norm)**	p-value	**< 0.001*****	**< 0.001*****	**< 0.001*****
	Effect Size	0.202	0.3615	0.563
**Path Length (L_norm)**	p-value	**< 0.001*****	**< 0.001*****	**< 0.001*****
	Effect Size	0.042	0.0377	0.080

Table S10: Patterning Comparisons (DF vs VF) by Age Group for Small World metrics, related to Figure 4.
Mixed-effects model *p < 0.05, **p < 0.01, ***p < 0.001.

Metric	Measure	Age Groups		
		23-33 days	34-45 days	46-64 days
**Small World (S)**	p-value	**< 0.001*** **	**< 0.001*** **	**< 0.001*** **
	Dorsal Mean	2.455	2.633	2.654
	Ventral Mean	3.137	3.300	3.372
**Clustering (C_norm)**	p-value	**< 0.001*** **	**< 0.001*** **	**< 0.001*** **
	Dorsal Mean	2.708	3.027	3.097
	Ventral Mean	3.723	3.925	4.286
**Path Length (L_norm)**	p-value	**< 0.001*** **	**< 0.001*** **	**< 0.001*** **
	Dorsal Mean	1.103	1.149	1.159
	Ventral Mean	1.206	1.248	1.285

Table S11: Statistical Comparison of modularity Across DF and VF age ranges, related to Figure 6.
Mixed-effects model *p < 0.017, **p < 0.003, ***p < 0.001 (Bonferroni corrected).

Comparison	p-value	Significant	Median	
			Group 1	Group 2
Dorsal vs Ventral (23-33 days)	0.30	No	0.2257	0.2814
Dorsal vs Ventral (34-45 days)	0.007	Yes**	0.2730	0.4836
Dorsal vs Ventral (46-64 days)	0.002	Yes**	0.2620	0.4349
Dorsal: 23-33 days vs 34-45 days	0.66	No	0.2257	0.2730
Dorsal: 34-45 days vs 46-64 days	0.74	No	0.2730	0.2620
Dorsal: 23-33 days vs 46-64 days	0.94	No	0.2257	0.2620
Ventral: 23-33 days vs 34-45 days	0.30	No	0.2814	0.4836
Ventral: 34-45 days vs 46-64 days	0.94	No	0.4836	0.4349
Ventral: 23-33 days vs 46-64 days	0.21	No	0.2814	0.4349

Table S12: Comparison of Rigid Unit Proportion Between DF and VF Organoids by Age Group, related to Figure 7
Mann-Whitney U test, * Significant at p < 0.05

Age Group (days)	DF Mean (± SD)	VF Mean (± SD)	U-statistic	p-value	Significant
23-33	0.033 ± 0.078	0.068 ± 0.155	39.5	0.86	No
34-45	0.076 ± 0.124	0.136 ± 0.270	582.5	0.16	No
46-64	0.112 ± 0.137	0.022 ± 0.035	640.0	< 0.001	Yes*
Overall	0.087 ± 0.131	0.079 ± 0.185	3164.0	0.001	Yes*

Table S13: Comparison of Bursting Dynamics Between DF and VF Organoids, related to Figure 7.
Kolmogorov-Smirnov test, * Significant at p < 0.05. SEM = Standard Error of Mean

Measure	DF Mean (SEM)	VF Mean (SEM)	p-value	Sig	Effect Size
Burst Correlation	0.239 ± 0.017	0.191 ± 0.014	0.001	Yes*	0.249
Timing Variability (ms)	95.2 ± 0.9	94.0 ± 1.4	0.02	Yes*	0.021

SUPPLEMENTAL METHODS

mESC Maintenance

mESCs were maintained on plates coated with 0.5 µg/mL recombinant human vitronectin (Thermo Fisher Scientific #A14700) in 1X PBS (pH~7.4; Thermo Fisher Scientific #70011044) for 15 min at room temperature. Cells were cultured in mESC maintenance medium consisting of Glasgow Minimum Essential Medium (GMEM; Thermo Fisher Scientific #11710035) supplemented with 10% embryonic stem cell-qualified fetal bovine serum (Thermo Fisher Scientific #10439001), 0.1 mM MEM Non-Essential Amino Acids (Thermo Fisher Scientific #11140050), 1 mM sodium pyruvate (Millipore Sigma #S8636), 2 mM GlutaMAX supplement (Thermo Fisher Scientific #35050061), 0.1 mM 2-mercaptoethanol (Millipore Sigma #M3148), 0.05 mg/mL Primocin (InvivoGen #ant-pm-05), and 1000 U/mL recombinant mouse leukemia inhibitory factor (Millipore Sigma #ESG1107), with daily medium changes.

Cells were passaged using ReLeSR (Stem Cell Technologies #05872) according to manufacturer instructions and cryopreserved in mFreSR medium (Stem Cell Technologies #05855). All lines were tested for mycoplasma, and tested negative, every 6-9 months (MycoAlert #Lonza LT07-318).

GMEM-Based DF Organoids Generation

mESCs were dissociated into single cells with TrypLE Express (Thermo Fisher Scientific #12604021) for 5 minutes at 37 °C and re-aggregated in Lipidure-coated 96-well V-bottom plates at 3,000 cells per well in 100 µL of differentiation medium. The medium consisted of Glasgow Minimum Essential Medium (GMEM Thermo Fisher Scientific #11710035) supplemented with 10% KnockOut Serum Replacement (Thermo Fisher Scientific #10828028), 0.1 mM MEM Non-Essential Amino Acids (Thermo Fisher Scientific #11140050), 1 mM Sodium Pyruvate (Millipore Sigma #S8636), 2 mM GlutaMAX (Thermo Fisher Scientific #35050061), 0.1 mM 2-Mercaptoethanol (Millipore Sigma #M3148), and 0.05 mg/mL Primocin (InvivoGen #ant-pm-05). Additional supplements included 20 µM Y27632 (Tocris Bioscience #1254), 3 µM IWR1-ε (Cayman Chemical #13659), and 5 µM SB431542 (Tocris Bioscience #1614). Medium was changed daily from days 0 to 7.

On day 7, organoids were transferred to ultra-low adhesion plates (Millipore Sigma #CLS3471) with N2 medium composed of DMEM/F12 with GlutaMAX (Thermo Fisher Scientific #10565018), 1X N2 Supplement (Thermo Fisher Scientific #17502048), and Primocin. Cultures were maintained on an orbital shaker at 60 rpm under 5% CO_2 with medium changes every 2-3 days.

From day 14 onward, organoids were cultured in neuronal maturation medium containing BrainPhys Neuronal Medium (Stem Cell Technologies #05790), supplemented with N2, Chemically Defined Lipid Concentrate (Thermo Fisher Scientific #11905031), B27 Supplement (Thermo Fisher Scientific #17504044), Primocin, and 0.5% (v/v) Matrigel GFR (Corning #354230).

Single-Cell Dissociation

Organoids (8-10 per genotype) were dissociated using the Worthington Papain Dissociation System (#LK003150). The dissociation solution consisted of 20 U/mL papain, 1 mM L-cysteine, and 0.5 mM EDTA in EBSS, activated for 30 min at 37 °C. DNase I (200 U/mL) was added post-activation. Tissue samples were incubated in this solution for 30 min at 37 °C with gentle agitation every ~10 min, then mechanically dissociated using flame-polished glass Pasteur pipettes (Fisher Scientific #13-678-6B). After centrifugation (300 RCF, 3 min), cells were resuspended in 1X PBS with 0.1% BSA (Millipore Sigma #A3311), filtered through a 40 μm strainer (Corning #431750), and manually counted.

scRNAseq and Computational Analysis

Sequencing was conducted on an AVITI PE75 Flowcell (Element Biosciences) at the UC Davis Technologies Core, generating approximately 900 million reads. Raw FASTQ files were processed using the PIPseeker pipeline (v3.3), with default parameters for alignment, transcript quantification, and cell calling against the GRCm39 mouse reference genome (GENCODE vM29 2022.04, Ensembl 106).

Downstream analysis was performed in Seurat (v5.1.0) (Hao et al., 2024) using sensitivity 5 matrices. Quality control steps included:

- Genotype demultiplexing with Souporcell (Heaton et al., 2020).
- Doublet removal using DoubletFinder v2.0.4 (McGinnis et al., 2019).
- Batch integration using Harmony (Korsunsky et al., 2019).

Cells were excluded if they had mitochondrial content > 20%, unique gene counts below the 5th percentile, or total RNA counts > 50,000. SCTransform was applied with mitochondrial regression,variable genes were selected (Lause et al., 2021; Choudhary and Satija, 2022). Dimensionality and 3,000 variable genes was performed using 40 principal components, followed by Leiden clustering at resolutions from 0.5 to 2. Clusters were visualized using UMAP (Becht et al., 2019), and resolution selection was guided by marker gene expression. Cell types were annotated using the Allen Brain Atlas (Yao et al., 2021}), UCSC Cell Browser (Speir et al., 2021), and the Arlotta developmental atlas (Di Bella et al., 2021). For reference mapping, dorsal forebrain datasets were integrated using Seurat's standard workflow: log-normalization (scale factor 10,000), variable gene selection, data scaling, and PCA (30 components). Harmony was used for integration prior to label transfer via canonical correlation analysis (CCA)-based anchor identification (Hao et al., 2024). Neuronal cells were subset and dimensionality reduction was redone after reperforming SCTransform to rescale neuronal genes.

Viability testing

Organoids were transferred to 1.5 mL microcentrifuge tubes containing 500 μL of culture medium. Acridine orange-propidium iodide (AOPI) dye (Revvity #CS2-0106-5ML) was added at a 1:1,000 dilution and incubated for 30 minutes at room temperature. Following incubation, organoids were washed with 1× PBS and imaged using an EVOS M7000 microscope.

Organoid size measurement

Organoids were imaged on a standard tissue culture microscope at 4× magnification using an iPhone 15 camera at 1× zoom. A calibration slide was used to establish a pixel-to-micrometer conversion. Organoid diameters were measured using the straight-line and Measure tools in Fiji (v1.54p). Measurements were exported as CSV files and analyzed using Python (v3.11).

qPCR

DF and VF organoids were collected at Day 30, Day 52, and Day 60 across three independent batches. For each condition and batch, three organoids derived from the same plate were pooled to form a single biological replicate (N=3 biological replicates per condition).

Total RNA was isolated from pooled organoid samples using TRIzol® reagent (Thermo Fisher Scientific #15596026) according to the manufacturer's instructions, involving chloroform extraction and isopropanol precipitation. The resulting RNA pellet was washed with 75% ethanol and resuspended. RNA concentration and purity were verified using a NanoDrop spectrophotometer. cDNA was synthesized from 1000 ng of total RNA using the SuperScript IV Reverse Transcriptase system (Thermo Fisher Scientific #18090010), employing oligo(dT) primers (Thermo Fisher Scientific #AM5730G). Key components included DTT (Thermo Fisher Scientific #707265ML) and RNaseOUT Recombinant RNase Inhibitor (Thermo Fisher Scientific #10777019). The cDNA was stored at −20 °C.

qPCR reactions were performed using TaqMan Gene Expression Assays (FAM; Thermo Fisher Scientific #4453320) on a QuantStudio Pro Real-Time PCR System (Thermo Fisher Scientific). We targeted Gapdh (housekeeping control), Camk2a (excitatory neuron marker), Pvalb (PV+ interneuron marker), and Gad2 (pan-GABAergic marker). Reactions were assembled using TaqMan Fast Advanced Master Mix (Thermo Fisher Scientific #4444556), cDNA template, and the corresponding TaqMan probe. Each batch included all six experimental conditions run with three technical replicates per gene. The standard cycling program was used: an initial hold (50°C for 2 min and 95°C for 10 min), followed by 40 cycles of denaturation (95°C for 15 s) and annealing/extension (60°C for 1 min). Fluorescence data was collected during each extension step.

Relative gene expression was determined by calculating ΔCt for each sample (Ct_target − Ct_Gapdh). These ΔCt values were converted to expression ratios using $2^{-\Delta Ct}$, providing a normalized measure proportional to transcript abundance. For both dorsal and ventral samples, expression ratios (Pvalb/Camk2a and Gad2/Camk2a) were calculated by dividing the corresponding normalized $2^{-\Delta Ct}$ values for each gene within the same sample. Statistical analyses were performed directly on these gene expression ratios.

Cryosection Immunohistochemistry

Organoids were fixed in 4% paraformaldehyde (Thermo Fisher Scientific #28908), cryoprotected in 30% sucrose (Millipore Sigma #S8501), and embedded in a 1:1 mix of Tissue-Tek O.C.T. Compound (Sakura #4583) and 30% sucrose. Cryosections (20 µm) were cut

using a Leica CM3050 and collected directly onto slides. After PBS washes, sections were blocked (5% donkey serum, 0.1% Triton X-100) for 1 h, incubated with primary antibodies overnight at 4 °C, washed, then incubated with secondary antibodies for 90 min at room temperature. After final washes, sections were mounted with Fluoromount-G (Thermo Fisher Scientific #00-4958-02).

Vibratome Section Immunohistochemistry

For whole-mount analysis, organoids were fixed in 4% PFA (4 °C, overnight), embedded in 4% low-melt agarose (Invitrogen #16520-050), and sectioned at 50 μm using a Leica VT1000s vibratome. Sections underwent sequential blocking:

Initial block: 5% donkey serum, 1% BSA, 0.5% Triton X-100 (4 °C, 1 h)
Antibody block: 2% donkey serum, 0.1% Triton X-100 with primary antibodies (overnight, 4 °C)

Following PBS washes, sections were incubated with secondary antibodies (30 min, RT), counterstained with Hoechst 33342 or DAPI, and mounted with Fluoromount-G (Fisher Scientific #OB100-01).

Antibody Panel and Imaging

The following primary antibodies were used for immunohistochemistry, listed alphabetically by target antigen:

- Anti-Brn2 (rabbit; Thermo Fisher Scientific #PA530124, RRID:AB_2547598; 1:400)
- Anti-Cox6A2 (rabbit; Novus Biologicals #NBP1-31112; RRID:AB_2085447; 1:100)
- Anti-Ctip2 (rat; Abcam #ab18465, RRID:AB_2064130; 1:250)
- Anti-GABA (rabbit; Thermo Fisher Scientific #PA5-32241, RRID:AB_2549714; 1:375)
- Anti-Gfap (mouse; Thermo Fisher Scientific #G6171, RRID:AB_1840893; 1:100)
- Anti-Map2 (chicken; Thermo Fisher Scientific #PA1-10005 ; RRID: AB_1076848; 1:1000)
- Anti-Map2 (rabbit; Proteintech #17490-1-AP, RRID:AB_2137880; 1:2000)
- Anti-N-cadherin (mouse; Abcam #ab98952, RRID:AB_10696943; 1:250)
- Anti-Nkx2.1 (rabbit; Abcam #ab76013, RRID:AB_1310784; 1:400)
- Anti-Parvalbumin (rabbit; Swant #PV27, RRID:AB_2631173; 1:375)
- Anti-Pax6 (mouse; BD Biosciences #561462, RRID:AB_10715442; 1:100)
- Anti-Pkcζ (mouse; Santa Cruz Biotechnology #sc17781, RRID:AB_628148; 1:500)
- Anti-Satb2 (mouse; Abcam #ab51502, RRID:AB_882455; 1:100)
- Anti-Sox2 (mouse; Santa Cruz Biotechnology #sc365823, RRID:AB_10842165; 1:500)
- Anti-Sst (mouse; Santa Cruz Biotechnology #sc55565, RRID:AB_831726; 1:100)

Secondary detection used Alexa Fluor-conjugated antibodies (1:750). Nuclear counterstaining was performed with 300 nM DAPI (Thermo Fisher Scientific #D1306).

For perineuronal nets detection we used and biotinylated WFA (Vector Laboratories #B-1355-2, RRID:AB_2336874; 1:200) followed by Alexa 488-streptavidin (Thermo Fisher Scientific #S11223; 1:500).

Analysis of Immunohistochemistry

Organoid imaging was performed on a Zeiss AxioImager Z2 microscope with 10x magnification using Zen Blue software. Z-stacks were acquired at 1.53 µm spacing from three non-adjacent cryosections per organoid, with tile scanning for larger samples. The analysis included 4-5 organoid replicates per cell line and condition (DF or VF) across two independent mESC lines (ES-E14TG2a and KH2).

Raw .czi files were converted to .ims format using the Imaris file converter, deconvolved in AutoQuant X3 3.1, and analyzed in Imaris (v10.2). Nuclear segmentation was performed on the DAPI channel. Spot detection used an XY diameter of 4.5 µm, PSF model elongation of 15 µm, background subtraction, quality filter threshold >1747, and nearest-neighbor distance between 4.83 and 12.0 µm.

For marker quantification, Pax6+ and Nkx2.1+ cells were identified using the same spot detection parameters, with additional colocalization filters requiring a maximum distance of 14 µm from DAPI+ nuclei. The pipeline was automated using Imaris Arena with consistent parameters across all patterning conditions.

Exported metrics included DAPI+ cell counts and percentages of Pax6+/DAPI+ and Nkx2.1+/DAPI+ double-positive cells. Statistical analysis was performed on 162 dorsal and 113 ventral images using a Mann-Whitney U test to compare proportions between conditions. Significance was defined as $p < 0.05$. Quality control measures included blinded analysis (experimenter masked to conditions).

For 3D image reconstruction, .czi files were converted and deconvolved as described above. Images and videos were exported using Imaris software (Oxford Instruments) and saved as PNG or MP4 files, respectively.

Electrophysiology Preparation

MEAs were coated with 0.01% polyethylenimine (Millipore Sigma #408727) in 1X PBS for 1 h at 37 °C, then washed three times with deionized water and air-dried for 10 min. They were subsequently coated with 20 µg/mL mouse laminin (Fisher Scientific #CB40232) and 5 µg/mL human fibronectin (Fisher Scientific #CB40008) in 1X PBS for 1 h at 37 °C. Organoids were placed onto the coated MEAs, excess medium was removed, and samples were incubated at 37 °C for 5-8 min to promote adhesion before adding pre-warmed neuronal differentiation medium.

Electrophysiological Data Processing

Electrophysiological activity was monitored every 2-3 days using Maxwell Biosystems acquisition software, sampling signals from 1024 of the ~26,000 electrodes in a sweeping checkerboard pattern (30 s per configuration). The 1,020 most active electrodes, spaced at least 50 µm apart, were selected for recording to ensure single-unit resolution. All recordings were performed in a humidified incubator (5% CO_2, 37 °C) at a sampling rate of 20 kHz and saved in HDF5 format.

Raw extracellular recordings were band-pass filtered between 300-6,000 Hz and spike-sorted using Kilosort2 (Pachitariu et al., 2016; Hill et al., 2011) within a custom Python pipeline. Units were excluded if they exhibited interspike interval (ISI) violation rates > 0.5, mean firing rates < 0.1 Hz, or signal-to-noise ratios (SNR) < 3.

Pharmacological Modulation of Neuronal Activity

DF organoids aged 60 to 65 days were scanned for spontaneous activity, and electrodes were selected based on the highest activity levels as described in the Electrophysiological Data Processing section.

Drug concentrations were selected based on established effective doses from previous studies (Mayer et al., 2019; van der Molen et al., 2025). Following a 10-minute baseline recording, the following pharmacological agents were applied:

- Gabazine (SR95531; Abcam #ab120042) at 1 μM.
- NBQX (Abcam #ab120045) at 20 μM.
- APV at 100 μM.

Stock solutions were prepared for 1:1000 dilution in culture medium. Gabazine and NBQX were dissolved in DMSO, and APV in water. After drug administration, organoids were incubated for 30 minutes before acquiring a 10-minute recording of drug-modulated activity.

Recordings were processed using the following pipeline:

- Concatenation using SpikeInterface (Hill et al., 2011).
- Spike sorting as described in the Electrophysiological Data Processing section.
- Manual curation using Phy visualization software (Rossant et al., 2013).

STTC Analysis

We quantified pairwise neuronal synchronization using the STTC with a $\Delta t = 10$ ms timescale (Chini et al., 2022; Chini et al., 2024; Cutts and Eglen, 2014) . The STTC is defined as:

$$STTC = \frac{1}{2}\left(\frac{P_A - T_B}{1 - P_A T_B} + \frac{P_B - T_A}{1 - P_B T_A}\right)$$

where:

- P_A is the proportion of spikes in train A that fall within $\pm\Delta t$ of any spike in train B.
- T_A is the proportion of the total recording duration that lies within $\pm\Delta t$ of spikes in train A.
- P_B and T_B are the corresponding quantities for spike train B.

This symmetric measure ranges from -1 (perfect anti-correlation) to +1 (perfect synchrony), with 0 indicating independence.

Functional Network Analysis

Network Construction

Functional connectivity matrices were derived from thresholded, binarized STTC values. To establish significance thresholds while preserving population rate dynamics, we:

1. Generated 1,000 surrogate datasets by spike identity shuffling.
2. Computed STTC distributions from shuffled data.
3. Set thresholds at the 90th percentile of null distributions.
4. Binarized matrices using these subject-specific thresholds.

Global Network Metrics

Using NetworkX (Hagberg et al., 2008) and custom Numba-accelerated functions, we computed:

- **Clustering coefficient**: Local density of connections using a Numba-accelerated parallel implementation (compute_clustering_coeff_parallel).
- **Characteristic path length**: Mean shortest path distance using NetworkX's (average_shortest_path_length) on the largest connected component.

All metrics were normalized by dividing by corresponding values from 100 synthetic random networks via generated (generate_random_graph) with identical node and edge counts.

Small-worldness was calculated as:

$$\text{Small-worldness} = \frac{C/C_{rand}}{L/L_{rand}}$$

where C and L are the clustering coefficient and path length, respectively. Binary functional networks were created using STTC values thresholded at the 90th percentile of surrogate values obtained by shuffling neuron identities across 1,000 randomized networks while preserving firing rate distributions.

Hub Identification

We computed a composite hubness score by integrating four nodal metrics:

- **Degree**: Number of connections (degrees_und).
- **Strength**: Sum of connection weights (strengths_und, using weighted matrices).
- **Betweenness centrality**: Fraction of shortest paths passing through nodes (betweenness_bin).
- **Closeness centrality**: Inverse average shortest path length (distance_bin derived).

Each metric was z-scored across nodes before summation to create the composite score.

Analysis computed:

- Firing rate distributions (mean ± SEM across replicates).
- Coefficient of variation (CV) of interspike intervals.
- Population synchrony (pairwise spike train correlations).
- E/I balance ratios (excitatory vs. inhibitory input currents).
- Weight distribution evolution (Kolmogorov-Smirnov tests).

SUPPLEMENTAL REFERENCES

Becht, E., McInnes, L., Healy, J., Dutertre, C., Kwok, I.W.H., Ng, L.G., Ginhoux, F. and Newell, E.W. (2019). Dimensionality Reduction for Visualizing Single-Cell Data Using UMAP. Nat. Biotechnol. *37*, 38-44.

Choudhary, S. and Satija, R. (2022). Comparison and Evaluation of Statistical Error Models for scRNA-seq. Genome Biol. *23*, 27.

Cutts, C.S. and Eglen, S.J. (2014). Detecting Pairwise Correlations in Spike Trains: An Objective Comparison of Methods and Application to the Study of Retinal Waves. J. Neurosci. *34*, 14288-14303.

Hagberg, A.A., Schult, D.A. and Swart, P.J. (2008). Exploring network structure, dynamics, and function using NetworkX. Proceedings of the 7th Python in Science Conference (SciPy2008) , 11-16.

Hao, Y., Stuart, T., Kowalski, M.H., Choudhary, S., Hoffman, P., Hartman, A., Srivastava, A., Molla, G., Madad, S., Fernandez-Granda, C., et al. (2024). Dictionary Learning for Integrative, Multimodal and Scalable Single-Cell Analysis. Nat. Biotechnol. *42*, 293-304.

Heaton, H., Talman, A.M., Knights, A., Imaz, M., Gaffney, D.J., Durbin, R., Hemberg, M. and Lawniczak, M.K.N. (2020). Souporcell: Robust Clustering of Single-Cell RNA-seq Data by Genotype Without Reference Genotypes. Nat. Methods *17*, 615-620.

Hill, D.N., Mehta, S.B. and Kleinfeld, D. (2011). Quality Metrics to Accompany Spike Sorting of Extracellular Signals. J. Neurosci. *31*, 8699-8705.

Korsunsky, I., Millard, N., Fan, J., Slowikowski, K., Zhang, F., Wei, K., Baglaenko, Y., Brenner, M., Loh, P. and Raychaudhuri, S. (2019). Fast, Sensitive and Accurate Integration of Single-Cell Data with Harmony. Nat. Methods *16*, 1289-1296.

Lause, J., Berens, P. and Kobak, D. (2021). Analytic Pearson Residuals for Normalization of Single-Cell RNA-seq UMI Data. Genome Biol. *22*, 258.

Mayer, S., Chen, J., Velmeshev, D., Mayer, A., Eze, U.C., Bhaduri, A., Cunha, C.E., Jung, D., Arjun, A., Li, E., et al. (2019). Multimodal Single-Cell Analysis Reveals Physiological Maturation in the Developing Human Neocortex. Neuron *102*, 143-158.e7.

McGinnis, C.S., Murrow, L.M. and Gartner, Z.J. (2019). DoubletFinder: Doublet Detection in Single-Cell RNA Sequencing Data Using Artificial Nearest Neighbors. Cell Syst. *8*, 329-337.e4.

Pachitariu, M., Steinmetz, N.A., Kadir, S.N., Carandini, M. and Harris, K.D. (2016). Fast and accurate spike sorting of high-channel count probes with KiloSort. 30th Conference on Neural Information Processing Systems (NIPS 2016), pp. 4455-4463. Barcelona, Spain.

Rossant, C., Hunter, M., Steinmetz, N., Wallace, M., Spacek, M., Gestes, C., McKenzie, Z., Nolan, C., Buccino, A., Zapp, S., et al. (2023). phy: Interactive Visualization and Manual Spike Sorting of Large-Scale Ephys Data [Python]. The Cortical Processing Laboratory at UCL.

Speir, M.L., Bhaduri, A., Markov, N.S., Moreno, P., Nowakowski, T.J., Papatheodorou, I., Pollen, A.A., Raney, B.J., Seninge, L., Kent, W.J., et al. (2021). UCSC Cell Browser: visualize your single-cell data. Bioinformatics *37*, 4578-4580.

Yao, Z., van Velthoven, C.T.J., Kunst, M., Zhang, M., McMillen, D., Lee, C., Jung, W., Goldy, J., Abdelhak, A., Aitken, M., et al. (2023). A High-Resolution Transcriptomic and Spatial Atlas of Cell Types in the Whole Mouse Brain. Nature *624*, 317-332.
